# Development of 2,1,3-Benzothiadiazole-Based Room-Temperature Fluorescent Nematic Liquid Crystals

**DOI:** 10.3390/molecules30112438

**Published:** 2025-06-02

**Authors:** Muhammad Suhail bin Uzair, Yoshimichi Shimomura, Takuya Tanaka, Takashi Kajitani, Gen-ichi Konishi

**Affiliations:** 1 Department of Chemical Science and Engineering, Institute of Science Tokyo, Tokyo 152-8552, Japan; uzair.m.aa@m.titech.ac.jp (M.S.b.U.); shimomura.y.ac@m.titech.ac.jp (Y.S.); tanaka.t.by@m.titech.ac.jp (T.T.); 2 Core Facility Center, Research Infrastructure Management Center, Institute of Science Tokyo, Yokohama 226-8501, Japan; kajitani.t.a5dc@m.isct.ac.jp

**Keywords:** nematic liquid crystal, fluorescent LC, room temperature LC, 2,1,3-benzothiadiazole

## Abstract

Fluorescent liquid crystals (LCs) have attracted considerable interest owing to their unique combination of fluidity, anisotropy, and intrinsic emission. However, most reported fluorescent LCs exhibit high phase transition temperatures and/or smectic phases, limiting their practical applications. To address this, we designed and synthesized a series of 2,1,3-benzothiadiazole (BTD)-based fluorescent nematic liquid crystals incorporating donor (D) or acceptor (A) groups to form D–A–D or D–A–A structures. Most of the synthesized derivatives exhibited supercooled nematic phases at room temperature. They composed various functional groups, such as secondary alkylamine, branched alkyl chain, and trifluoroacetyl groups, which are rarely used in calamitic nematic LCs. Notably, dimethylamine- and carbonyl-substituted derivatives exhibited relatively high fluorescence quantum yields (Φ_fl_) in both solid and mesophase states, demonstrating their potential as efficient fluorescent materials. Our findings underscore the versatility of BTD-based mesogenic skeletons for designing room-temperature fluorescent nematic LCs with various functional groups. These materials offer promising opportunities for next-generation display technologies, optical sensors, and photonic applications.

## 1. Introduction

Liquid crystals (LCs) have attracted considerable attention owing to their unique combination of fluidity and anisotropy. This dual nature makes them widely applicable in self-assembled materials [1,2,3,4,5], optoelectronics [6,7,8,9,10,11,12,13,14,15], and stimuli-responsive devices [16,17,18,19,20,21,22,23,24]. The molecular design and synthesis of new liquid crystalline molecules [25,26,27,28,29,30,31,32,33,34,35,36,37,38,39,40] and their materials [41,42,43,44,45,46,47,48,49,50,51,52] is still an important topic today. Among the various liquid-crystalline materials, fluorescent LCs have garnered significant interest because of their tunable optical properties and intrinsic anisotropy. These properties allow fluorescent LCs to exhibit both efficient fluorescence and favorable charge-transport characteristics, making them attractive for applications in organic light-emitting diodes (OLED) [53,54], sensors [55], and photonic devices [56].

A common strategy for designing fluorescent LCs is to introduce mesogenic moieties onto a rigid fluorescence core. One widely adopted approach involves incorporating π-conjugated fluorophores (e.g., pyrene [57,58,59], anthracene [60,61,62,63]) as mesogenic cores while employing alkyl/alkoxy chains as flexible tails. Additionally, the use of molecules substituted with electron-donor and/or acceptor functional groups has been investigated [64]. However, the majority of previously reported fluorescent LCs exhibit high phase transition temperatures and/or smectic phases, which are characterized by high viscosity and numerous structural defects. These limitations create challenges in the fabrication of LC devices and hinder the performance of self-assembled materials.

Nematic LCs present a promising solution for addressing these issues [65,66,67,68]. In contrast to smectic LCs, where molecules align unidirectionally and form layered structures, nematic LCs exhibit long-range orientational order without positional order, resulting in lower viscosity and fewer structural defects. Room-temperature nematic LCs of organic π-conjugated molecules lead to applications in stimulus-responsive optoelectronic devices. Recently, our group developed fluorescent calamitic nematic LCs at room temperature based on phenyl stilbene and phenyltolane skeletons [69,70,71]. Additionally, we synthesized biphenyl- and tolane-based fluorescent nematic LCs with secondary alkylamine and cyano groups [72], all of which exhibited blue emission with good fluorescence quantum yields in their nematic phases at room temperature. However, achieving room-temperature fluorescent nematic LCs with emission colors beyond blue, such as green, yellow, and red, remains a significant challenge.

We focus on 2,1,3-benzothiadiazole (BTD), which has been widely recognized as a versatile mesogenic core due to its strong electron-withdrawing nature, high fluorescence efficiency, and ability to modulate electronic properties through functionalization. Studies have shown that symmetrically functionalized BTD derivatives exhibit nematic and smectic phases [73,74,75]. Recently, Gόmez-Lor et al. reported low temperature asymmetrical 2,1,3-benzothiadiazole (BTD)-based fluorescent nematic LCs [76]. Their molecular structures incorporated 4,7-diphenyl-BTD as a mesogen, a nonyl chain at one of the ends, and dimethylamine (NMe_2_), methoxy (OMe), cyano (CN), or nitro (NO_2_) at the other. We considered that room-temperature nematic LCs could be realized by devising substituents. Although there was no data about their fluorescence quantum yields in the report, the 4,7-diphenyl-BTD mesogen appeared suitable for developing multicolor fluorescence nematic LCs.

Next, we used secondary amines in the tail to lower the phase transition temperature and achieve longer fluorescence wavelengths through push-pull architecture. Generally, secondary alkylamine groups and branched alkyl chains are not used as tails in calamitic nematic LCs, because they excessively suppress molecular alignment [72]. Moreover, functional groups whose dipole moments are tilted with respect to the molecular long axis, such as aldehyde and acetyl, are rarely incorporated, for the same reason. However, as mentioned previously, our group revealed that secondary alkylamine groups are an effective molecular design for room-temperature nematic LCs. 4,7-Diphenyl-BTD mesogen has a larger π-skeleton than biphenyl and tolane; thus, these substituent groups are useful for the development of room-temperature fluorescent nematic LCs in BTD-based mesogen.

In this study, we systematically synthesized new BTD-based LCs incorporating various terminal groups (cyano, aldehyde, acetyl, trifluoroacetyl, and trifluoromethyl) and flexible tails (alkyl, alkoxy, and alkylamine). Although the trifluoroacetyl group is not a common functional group for LCs, we used it because our research group recently developed ratiometric thermometer dyes by incorporating it [77]. By investigating their phase transition behaviors and photophysical properties, we achieved supercooled fluorescent nematic LCs at room temperature with green, orange, and red emission, incorporating unusual functional groups and tails for calamitic nematic LCs.

## 2. Results and Discussion

### 2.1. Synthesis of BTD Derivatives

**Scheme 1:**
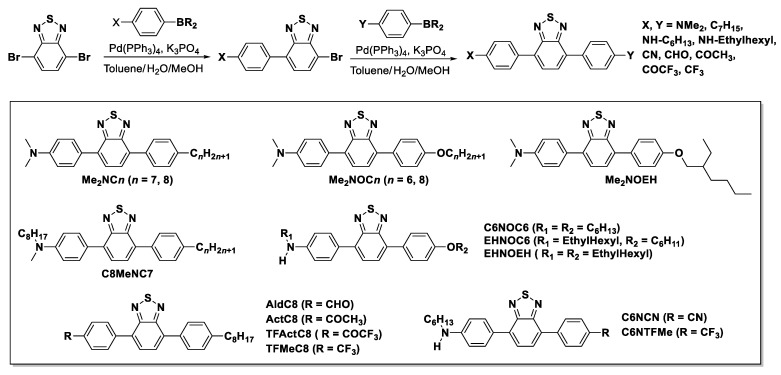
Synthetic procedures for BTD LCs.

**Figure 1:**
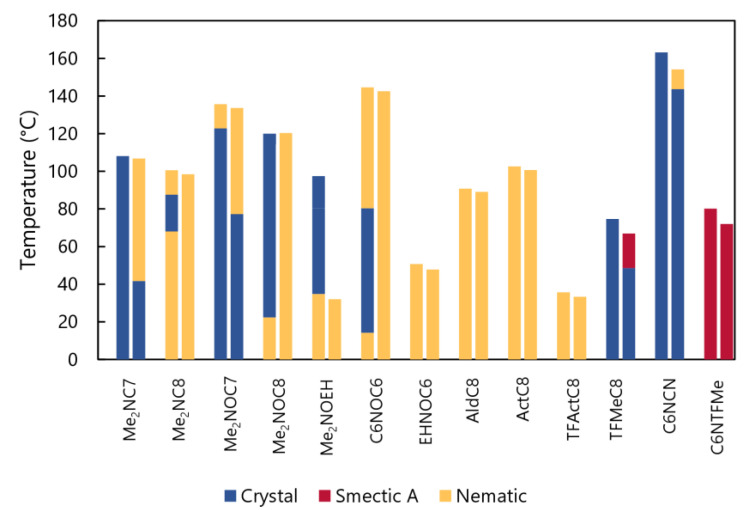
Schematic diagram showing the phase transition temperatures and range of BTD liquid crystals, excluding C8MeNC7 and EHNOEH; left bar: upon heating, right bar: upon cooling.

All BTD derivatives were synthesized via a palladium-catalyzed Suzuki–Miyaura cross-coupling reaction [78,79] (Scheme 1). Aryl boronic acids were either purchased or synthesized (see the Supplementary Materials). In the first step, 4,7-dibromo-2,1,3-benzothiadiazole was used as the starting material in excess (1.5–2.0 eq) to obtain mono-coupling compounds. These intermediates then underwent a second Suzuki–Miyaura cross-coupling reaction with an excess of aryl boronic acids to afford the target compounds listed in Figure 1. The structures of target compounds were characterized via ^1^H-NMR, ^13^C-NMR, Fourier transform infrared spectroscopy, and high-resolution electron ionization mass spectrometry. Spectral data and synthesis of boronic acids were compiled in Supplementary Materials.

### 2.2. Phase Transition Behaviors of BTD Derivatives

The phase transition behaviors of the synthesized compounds were evaluated using differential scanning calorimetry (DSC) and polarized optical microscopy (POM). POM observations were conducted to examine the mesogenic behaviors upon cooling and heating at a rate of 10 °C min^−1^ for molten samples sandwiched between untreated glass plates. The phase transition temperatures and enthalpies obtained from the third heating and cooling cycles of DSC measurements at a rate of 10 °C min^−1^ are presented in Table 1 and Figure 1.

#### 2.2.1. Me_2_NCn, Me_2_NOCn (*n* = 7 or 8), and Me_2_NOEH

Me_2_NC7 formed a monotropic nematic phase, displaying dark region and a schlieren texture under POM only during cooling (Figure S14a). In the DSC thermograms, an endothermic peak was observed at 108.1 °C upon heating, corresponding to the crystalline–isotropic phase transition (Figure S1). Upon cooling, two exothermic peaks were observed at 106.8 and 41.7 °C, attributed to the isotropic–nematic and nematic–crystalline phase transitions, respectively.

Me_2_NC8 exhibited enantiotropic nematic liquid-crystalline behavior. Dark region and a schlieren texture were observed under POM upon both heating and cooling (Figure S15a,b). DSC thermograms showed an exothermic peak at 68.0 °C and two endothermic peaks at 87.6 and 100.6 °C during heating, and an exothermic peak at 98.4 °C during cooling (Figure S2). The exothermic peak observed during heating suggests cold crystallization at 87.6 °C, likely due to the low crystallization rate. This phenomenon has also been reported for Me_2_NC9 [76]. Although there was no crystallization peak upon cooling, crystallization occurred at room temperature after 1 day under POM (Figure S15c).

Me_2_NC*n* had an odd–even effect; when *n* was 7 or 9 (odd), Me_2_NC*n* exhibited monotropic nematic phases and crystallized above room temperature in cooling, and when *n* was 8 (even), Me_2_NC*n* exhibited the enantiotropic nematic phase and did not crystalize in cooling. This odd–even effect was due to differences in the average shape of the alkyl chain; for odd *n*, the CH_2_-CH_3_ bond lies parallel to the molecular long axis, whereas for even *n*, it lies at an angle to the axis. This behavior is common in calamitic nematic LCs, such as *n*CB.

Me_2_NOC7 exhibited an enantiotropic behavior and displayed a schlieren texture under POM (Figure S16a,b). The DSC thermograms of Me_2_NOC7 exhibited two endothermic peaks during heating at 122.8 and 135.7 °C, corresponding to the crystalline-nematic and nematic-isotropic phase transitions, respectively (Figure S3). During cooling, two exothermic peaks were observed at 133.7 and 77.3 °C, corresponding to the isotropic–nematic and nematic–crystalline phase transitions, respectively.

In contrast, Me_2_NOC8 showed a monotropic nematic phase, and dark region and a schlieren texture under POM (Figure S17c). The DSC thermogram of Me_2_NOC8 displayed two exothermic peaks at 22.4 and 36.3 °C, along with two endothermic peaks at 114.2 and 120.0 °C, during heating (Figure S4). Additionally, a single exothermic peak was observed at 120.3 °C during cooling. POM observations revealed no change in the optical texture at 36 °C upon heating, indicating that this peak corresponded to a crystalline–crystalline phase transition. While, partial isotropization was observed at 114.2 °C and the residue was melted perfectly at 120 °C under POM (Figure S17a,b). The DSC measurements and POM observations suggest that Me_2_NOC8 undergoes a slower crystallization process than Me_2_NOC7.

Me_2_NOEH, which contains a 2-ethylhexyl chain as a tail, exhibited a monotropic nematic phase. A schlieren texture was observed upon cooling under POM (Figure S18b), and after 1 day at room temperature, crystallization was also observed (Figure S18c). DSC thermograms displayed one exothermic peak at 34.8 °C and two endothermic peaks at 80.4 and 97.3 °C during heating, along with an exothermic peak at 32.0 °C during cooling (Figure S5). However, the appearance of the two endothermic peaks upon heating was not consistent with that of the POM observation; the crystalline-isotropic phase transition was observed at 80 °C under POM. Then, we repeated the POM observation and found that Me_2_NOEH crystallized at 90 °C when applied with stress (Figure S18a). Moreover, it looks like that an exothermic peak is overlapping with the exothermic peak at 80.4 °C upon heating in the DSC thermogram. Therefore, the melting of the crystalline phase and crystallization occurred simultaneously at this temperature; Me_2_NOEH did not form a nematic phase upon heating. The DSC data indicated that the crystallization and isotropic–nematic phase transition occurred at a lower rate because of the branched structure of the tail.

#### 2.2.2. C8NMeC7, C6NOC6, EHNOC6, and EHNOEH

C8NMeC7 did not exhibit any mesophase (Figure S6), likely owing to the steric hindrance caused by the methyl group in the amine. The same phenomenon has been observed in biphenyl- and tolane-based fluorescent LCs with alkylamine and cyano terminal groups [72].

C6NOC6 exhibited enantiotropic nematic liquid-crystalline behavior. A schlieren texture was observed upon both heating and cooling under POM (Figure S19a,b). DSC thermograms showed two exothermic peaks at 14.3 and 48.4 °C and two endothermic peaks at 80.4 and 144.6 °C during heating, along with a single exothermic peak at 142.6 °C (Figure S7). No phase transition was observed near 48.4 °C under POM. Therefore, we assigned the exothermic peak at 14.3 °C to nematic–crystalline phase transition, the exothermic peak at 48.4 °C to a crystalline–crystalline phase transition, and the exothermic peaks at 80.4 and 144.6 °C to the crystalline–nematic and nematic–isotropic phase transitions during heating, respectively. The exothermic peak observed upon cooling corresponded to the isotropic–nematic phase transition.

**Figure 2:**
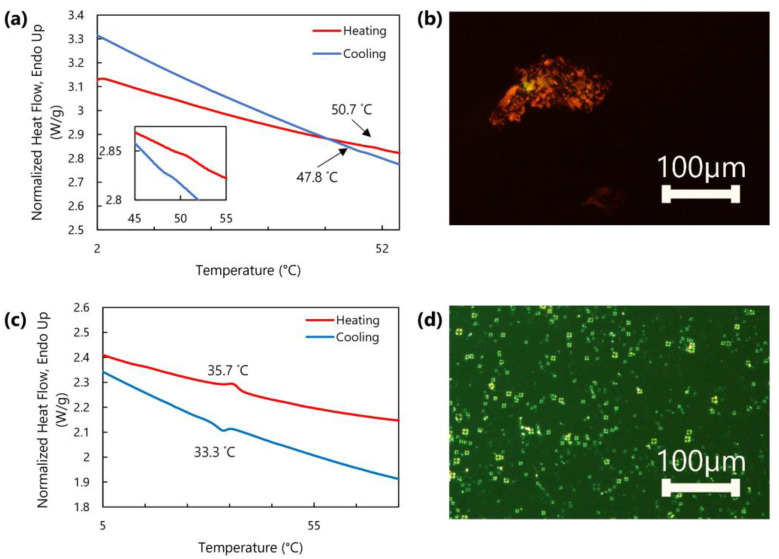
DSC thermograms of (**a**) EHNOC6 and (**c**) TFActC8 recorded at a heating rate of 10 °C min^−1^, and POM images of (**b**) EHNOC6 and (**d**) TFActC8 at 30 °C.

EHNOC6 exhibited a nematic phase and displayed dark region and a schlieren texture at room temperature under POM (Figure 2b). After one week at room temperature, crystallization occurred (Figure S20). The DSC thermograms recorded at a heating and cooling rate of 10 °C min^−1^ showed only a single endothermic peak during heating and a single exothermic peak during cooling (Figure 2a). These observations suggest that the crystallization was very slow owing to the steric hindrance of the branched tail structure. The previously reported 4,7-diphenyl-BTD LCs have a linear alkyl tail structure [76]. For such long mesogens, achieving a liquid crystalline phase with a branched alkyl tail improves solubility and decreases crystallinity, which is a significant advantage in the molecular design of the advanced materials.

However, EHNOEH did not exhibit any mesophase both under POM and in the DSC thermograms (Figure S8). The results suggest that incorporating 2-ethylhexyl tails at both ends is unfavorable for mesophase formation in this mesogenic structure.

#### 2.2.3. AldC8, ActC8, TFActC8, and TFMeC8

AldC8, ActC8, and TFActC8, which are carbonyl derivatives, exhibited nematic liquid-crystalline behavior, displaying dark region with bright boundary lines under POM (Figures S21a, S22a–e and Figure 2d). The DSC thermograms of AldC8 showed a single endothermic peak at 90.8 °C during heating and a single exothermic peak at 89.1 °C during cooling, both corresponding to the nematic–isotropic phase transition (Figure S9). The absence of a crystallization peak during cooling suggests that slow crystallization occurred after only 2 days at room temperature (Figure S21b).

The DSC thermogram of ActC8 exhibited four endothermic peaks at 58.2, 66.4, 75.6, and 102.6 °C during heating and a single exothermic peak at 100.7 °C during cooling (Figure S10). However, no phase transitions were observed under POM during heating below 102.6 °C (Figure S22a–d). Therefore, the three endothermic peaks at 58.2, 66.4, and 75.6 °C may correspond to nematic–nematic phase transitions. ActC8 also showed slow crystallization after overnight at room temperature (Figure S22f).

TFActC8 exhibited an endothermic peak at 35.7 °C during heating and an exothermic peak at 33.3 °C during cooling, along with a glass transition at 13 °C, in the DSC thermograms (Figure 2c). The endothermic and exothermic peaks correspond to the nematic–isotropic phase transitions. The crystallization of TFActC8 was very slow, with crystallization occurring only after a week at room temperature, as observed under POM (Figure S23).

In contrast, TFMeC8, which is not a carbonyl derivative, exhibited monotropic smectic liquid-crystalline behavior. A focal conic fan texture was observed under POM (Figure S24a), and the DSC thermograms showed one endothermic peak at 74.7 °C upon heating and two endothermic peaks at 66.9 and 48.6 °C upon cooling (Figure S11). The enthalpy change (Δ*H*) of the endothermic peak at 66.9 °C during cooling was −3.2 kJ mol^−1^, which is relatively high compared with typical nematic–isotropic phase transitions, suggesting that this phase was smectic A.

#### 2.2.4. C6NCN and C6NTFMe

C6NCN exhibited monotropic nematic liquid-crystalline behavior. A schlieren texture was observed under POM only during cooling (Figure S25a). The DSC thermograms showed one endothermic peak at 163.2 °C during heating and two exothermic peaks at 154.2 and 143.6 °C during cooling (Figure S12). The endothermic peak corresponds to the crystalline–isotropic phase transition, while the exothermic peaks at 153.2 and 143.6 °C correspond to the isotropic–nematic and nematic–crystalline phase transitions, respectively.

C6NTFMe exhibited a monotropic smectic A phase. Although no phase transition peaks were detected in the DSC thermograms (Figure S13), a focal conic fan texture was observed under POM (Figure S26a). The isotropic–smectic and smectic–crystalline phase transitions upon cooling proceeded slowly, with the former occurring between 72 and 55 °C and the latter occurring only after one week at room temperature. Upon heating, the crystalline–isotropic phase transition was observed at 80.2 °C.

In summary, BTD-based terphenyl-like molecules substituted with various functional groups, such as dimethylamine, secondary alkylamine, branched alkyl chain, aldehyde, acetyl, and trifluoroacetyl, exhibited room-temperature nematic liquid-crystalline behavior and were likely to align homeotropically. The results suggest that this mesogenic skeleton offers high molecular design flexibility for the development of room-temperature nematic LCs.

#### 2.2.5. A Comparison of Liquid Crystallinity with Previous Studies

**Figure 3:**
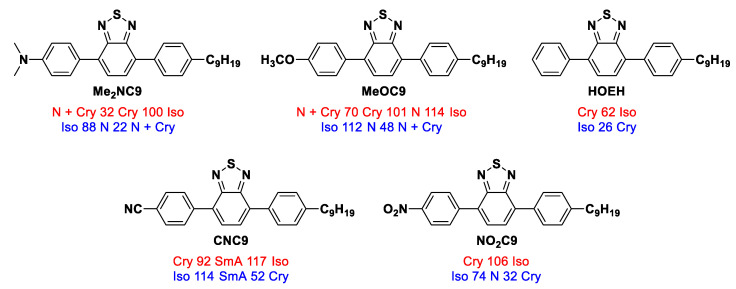
The structures of previously reported BTD derivatives [76] for comparison of their liquid crystallinity with the compounds in this study. Red: heating, Blue: cooling.

The novel liquid crystalline behavior of the BTD derivatives in this study will be compared with previous studies. ActC8 and TFActC8 exhibited nematic phases at lower temperatures than the previously reported BTD derivatives shown in Figure 3. Furthermore, ActC8 and TFActC8 did not crystallize and remained in a stable nematic phase at room temperature.

CNC9 [76] with tertiary amines in the tail exhibited only a smectic A phase, while C6NCN with secondary amines achieved a nematic phase in this study.

Me_2_C8 (this work) and Me_2_C9 (previous work) exhibited an enantiotropic and monotropic nematic phase, respectively. Furthermore, Me_2_C8 did not crystallize. Consequently, BTD derivatives have an odd-even effect, with even-numbered alkyl tails showing superior properties.

### 2.3. Photophysical Properties of BTD Derivatives

**Figure 4:**
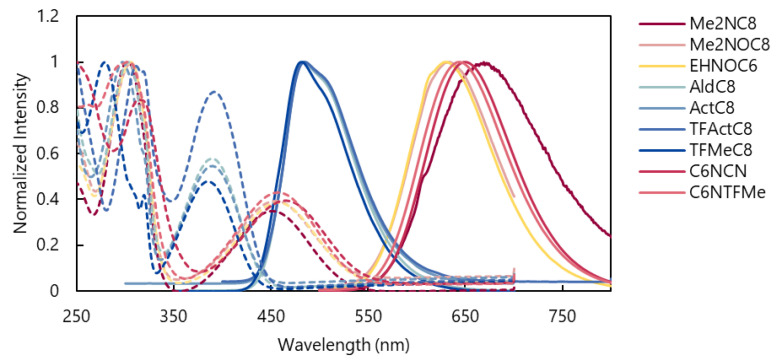
Absorption (dashed line) and fluorescence (bold line) spectra of BTD derivatives in a 10^−5^ M THF solution.

To investigate the photophysical properties of the representative compounds (Me_2_NC8, Me_2_NOC8, EHNOC6, AldC8, ActC8, TFActC8, TFMeC8, C6NCN, and C6NTFMe), in solution, the mesophase, and the solid state, we measured their absorption spectra, fluorescence spectra, and fluorescence quantum yields (Φ_fl_) (Table 2 and Figure 4). TFMeC8 and C6NTFMe exhibited a smectic A phase, whereas all other compounds were nematic. The representative derivatives were selected owing to their similarities in phase transition behavior and chemical structure. The photophysical properties of individual derivatives are compiled in the Supplementary Materials (Figures S27–S35). Measurements in the mesophase were conducted by melting samples in a polyimide-coated aligned cell with a thickness of 3 μm. The fluorescence spectra in the mesophase were recorded using a temperature controller. The Φ_fl_ in the mesophase was measured by heating the sample to 70 °C with the temperature controller, followed by rapid measurement in an integrating sphere.

#### 2.3.1. Absorption Properties in THF Solution

The absorption spectra were measured in a 1.0 × 10^−5^ M THF solution. Regardless of the terminal groups, all derivatives exhibited two absorption bands: one in the range of 279–306 nm and another in the range of 384–465 nm. The higher-energy band is associated with the π-π* transition, while the lower-energy band corresponds to the charge-transfer (CT) transition, which is influenced by the terminal groups [27,28,29,30,31,32,33,34,35,36,37,38,39]. The molar absorption coefficients (*ε*) ranged from 11,000 to 37,000 M cm^−1^, with the π-π* transition consistently exhibiting a higher *ε* than the CT transition. Me_2_NC8, Me_2_NOC8, C6NOC6, and EHNOC6 exhibited similar characteristics, with their CT bands (*λ*_abs_ = 451–459 nm), redshifted compared with those of AldC8, ActC8, TFActC8, and TFMeC8 (*λ*_abs_ = 384–391 nm). This redshift is attributed to electron-donating properties of amine groups.

C6NCN exhibited the longest CT band (*λ*_abs_ at 465 nm), which was redshifted compared with derivatives containing amine and alkyl or alkoxy groups. The *λ*_abs_ was redshifted by 77 nm relative to that previously reported for CNC9, which has cyano and nonyl alkyl terminal groups [76]. Meanwhile, the CT band of C6NTFMe appeared at 456 nm, which was redshifted by 65 nm compared with that of TFMeC8. This phenomenon is due to the strong electron-donating effect of the amine group.

Interestingly, Me_2_NOC8, C6NOC6, and EHNOC6 exhibited a donor–acceptor–donor (D-A-D) configuration, whereas C6NCN and C6NTFMe exhibited a D-A-A (i.e., push–pull) configuration. Despite these structural differences, both configurations showed only minor shifts in absorption wavelength. A similar trend in absorption-band shifts was observed in a previous study [80]. Moreover, C6NTFMe and other D-A-D derivatives exhibited similar absorption wavelengths, whereas C6NCN was redshifted by 9 nm compared with C6NTFMe, owing to its stronger electron-withdrawing properties.

#### 2.3.2. Fluorescence Properties in THF Solution

For each BTD derivative, the fluorescence spectra obtained upon excitation at the π-π* transition bands and CT bands were almost identical, indicating that their fluorescence originates from CT states.

In a THF solution, Me_2_NC8, Me_2_NOC8, and EHNOC6 exhibited red fluorescence. The maximum emission wavelength (*λ*_em_) of Me_2_NC8 was 661 nm, with a Φ_fl_ exceeding 0.86. In contrast, the *λ*_em_ values of Me_2_NOC8 and EHNOC6 were 657 and 650 nm, respectively, with Φ_fl_ values ranging from 0.55 to 0.77—slightly shorter and lower compared with Me_2_NC8. Notably, the *λ*_em_ of Me_2_NOC8 and EHNOC6 in the THF solution remained relatively unchanged despite variations in alkoxy chain length and the substitution of a secondary amine with a tertiary amine. This result suggests that changes in alkoxy chain length and structure have a negligible impact and that the *λ*_em_ is primarily affected by the nature of the substitution group.

The derivatives containing an alkyl chain and an acceptor group (AldC8, ActC8, TFActC8, and TFMeC8) exhibited green fluorescence (*λ*_em_ = 493–495 nm), which was blue-shifted relative to derivatives with amine groups. The *λ*_em_ was hardly affected by the electron-withdrawing group. The Φ_fl_ of these derivatives varied between 0.30 and 0.70 upon excitation at a high energy (π-π* band) but approached unity (0.99) upon excitation at a low energy (CT band), indicating an efficient CT transition. C6NCN and TFMeNC6 exhibited fluorescence in the red region, with *λ*_em_ at 674 and 667 nm, respectively. Their Φ_fl_ values were relatively low, ranging from 0.21 to 0.52, compared with other derivatives.

#### 2.3.3. Fluorescence Properties in Polycrystalline State

**Figure 5:**
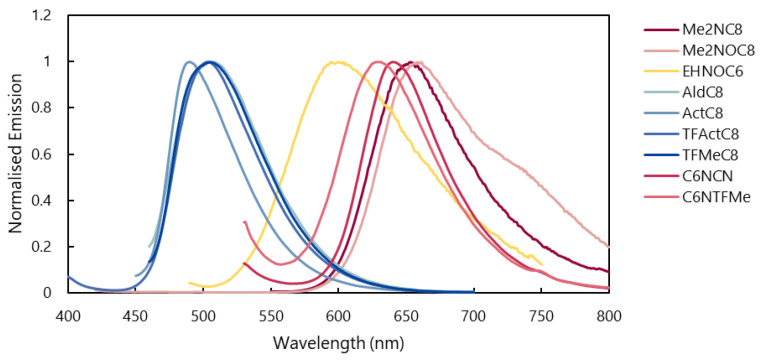
Emission spectra of BTD derivatives in polycrystalline state.

In the polycrystalline state, the derivatives exhibited fluorescence ranging from cyan (490 nm) to red (658 nm) (Figure 5).

Me_2_NC8 and EHNOC6 showed orange fluorescence, with *λ*_em_ at 592 and 591 nm, respectively, which were shorter than those in the THF solution. Me_2_NOC8 exhibited red fluorescence with *λ*_em_ at 658 nm, which remained relatively consistent with its *λ*_em_ in the THF solution. Overall, Me_2_NC8, Me_2_NOC8, and EHNOC6 had consistent Φ_fl_ values in the THF solution and solid state (Φ_fl_ in solid state: 0.80, 0.56, and 0.50, respectively), suggesting efficient emission in both media.

AldC8, ActC8, TFActC8, and TFMeC8, which contain electron-withdrawing acceptor groups, exhibited blue-green fluorescence, with Φ_flu_ values of 0.50, 0.66, 0.28, and 0.59, respectively. In contrast to amine and alkyl/alkoxy derivatives, the *λ*_fl_ values of these acceptor derivatives in their solid states were redshifted by 20–27 nm compared with their values in the THF solution. Previous reports [76,77,78,79,80,81,82,83] mentioned that the BTD core participates in intermolecular interactions with terminal substituents in single crystals, influencing self-assembly tendencies. Therefore, differences in the nature of the substituents (donor vs. acceptor) may account for the variations in their emission behaviors.

C6NCN and TFMeNC6 exhibited red fluorescence (*λ*_fl_ = 642 and 644 nm, respectively), but with low Φ_flu_ values of 0.08. In addition to containing secondary amine groups, they both possess strong electron-withdrawing groups (cyano and trifluoromethyl). This may contribute to strong intermolecular interactions in aggregates [84], leading to fluorescence quenching in the solid state. Nevertheless, it is important to note that fluorescence in the solid state is affected by aggregate formations [85,86], intermolecular interactions, and molecular packing structures [87]. Therefore, it is difficult to definitively determine the cause of the observed behavior for all the derivatives.

#### 2.3.4. Fluorescence Properties in Mesophase

The Φ_fl_ of C6NCN in the nematic phase could not be measured, owing to its high transition temperatures and narrow temperature range (143.6–154.2 °C).

**Figure 6:**
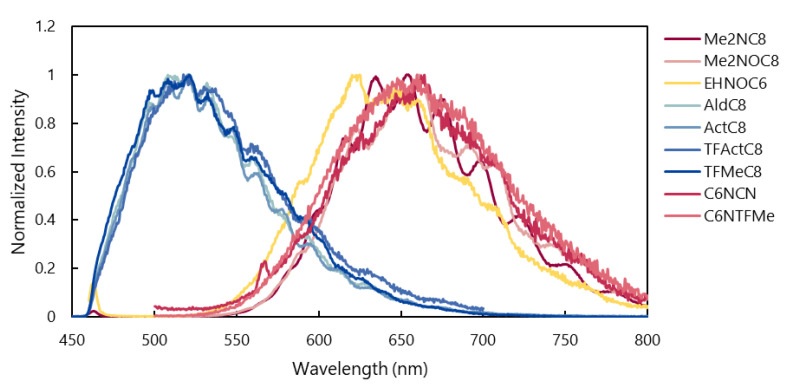
Emission spectra of BTD derivatives in mesophase using polyimide-aligned cells (3 μm gap). The data shown correspond to the same samples used for the fluorescence quantum yield measurements listed in Table 2.

Amine derivatives (Me_2_NC8, Me_2_NOC8, EHNOC6, C6NCN, C6NTFMe) exhibited red fluorescence (*λ*_fl_ = 640–652 nm), whereas the other derivatives (AldC8, ActC8, TFActC8, TFMeC8) exhibited green fluorescence (*λ*_fl_ = 513–529 nm). The *λ*_fl_ values in the mesophase were generally redshifted compared with those in the solid states, which is consistent with previous reports [76,79]. While detailed studies on fluorescence behavior in mesophases are limited, the fluorescence wavelength shift relative to the solid states suggests that aggregation structures and luminescent species differ between the mesophase and solid states (Figure 6).

Although the Φ_fl_ values of most of the derivatives were approximately 0.10, which is lower than those in the solid states, Me_2_NC8, Me_2_NOC8, and TFMeC8 exhibited relatively high Φ_fl_ values (0.36, 0.28, and 0.56, respectively). In mesophases, molecules have lower viscosity than in the solid state, and the measurements of Φ_fl_ were conducted at close to 70 °C. These factors promote molecular rotations and vibrations, contributing to the reduction in Φ_flu_ for nematic BTD-based LCs [88,89]. In the case of the smectic LC TFMeC8, molecules formed a layered structure in the mesophase, which is structurally closer to the crystalline phase than nematic LCs. This may explain why TFMeC8 maintained a high Φ_flu_.

In summary, BTD-based terphenyl-like molecules substituted with various functional groups, including dimethylamine, aldehyde, acetyl, trifluoroacetyl, trifluoromethyl, and cyano, exhibited different emission colors and relatively high Φ_fl_ values in both the solid state and solution. Moreover, derivatives containing dimethylamine (Me_2_NC8 and Me_2_NOC8), carbonyl groups (AldC8 and ActC8), and trifluoromethyl (TFMeC8) exhibited relatively high Φ_fl_ values in the mesophase. This molecular structure provides a robust design strategy for achieving tunable emission colors in the mesophase at low temperatures.

## 3. Experimental Method

### 3.1. Materials

Unless otherwise noted, all reagents and chemicals were received and used without further purification. All purchased materials are compiled in Supplementary Materials.

### 3.2. Instruments

^1^H-NMR and ^13^C-NMR spectra were recorded on BRUKER 500 (Yokohama, Japan) (500 MHz) and JEOL 400 (Tokyo, Japan) (100 MHz) spectrometers, respectively, for CDCl_3_ solution using tetramethylsilane (TMS) as an internal standard. ^1^H-NMR spectra were reported as follows: chemical shift (δ ppm), multiplicity (s = singlet, d = doublet, t = triplet, q = quartet, m = multiplet), integration, and coupling constants in units of Hz. ^13^C-NMR spectra were reported as chemical shifts in ppm. The FT-IR spectra were recorded on a JASCO FT-IR 4600 spectrometer (Tokyo, Japan). HRMS. Polarized optical microscopy (POM) was performed using Olympus BX51 optical microscope (Tokyo, Japan) with a Mettler FP90 hot stage (Greifensee, Switzerland) at a rate of 10 °C min^−1^. Differential scanning calorimetry (DSC) was performed using PerkinElmer DSC 8500 equipment (Waltham, MA, USA) at a scanning rate of 10 °C min^−1^ under a flow of dry nitrogen. UV-Vis spectra were recorded on a JASCO V-670 UV-vis spectrophotometer (Tokyo, Japan). Fluorescence spectra and absolute quantum yields were measured by Edinburgh Instruments fluorescence spectrometer FS5 (Edinburgh, UK). All photophysical measurements were performed using dilute solutions with optical densities (ODs) around 0.1 at the maximum absorption wavelength in 1 cm path length quartz cells at room temperature (298 K). In addition, all sample solutions were de-aerated by bubbling with argon gas for 15 min prior to the quantum yield.

### 3.3. Synthesis

Synthetic intermediates were used without further purification if there were no problems in the following reactions.

**4-(7-bromobenzo[c][1,2,5]thiadiazol-4-yl)-*N,N*-dimethylaniline**

4,7-dibromobenzo[c][1,2,5]thiadiazole (1.18 g, 4.0 mmol), (4-(dimethylamino)phenyl)boronic acid (0.38 g, 2.3 mmol), K_3_PO_4_ (1.27 g, 6.0 mmol) and Pd(PPh_3_)_4_ (3.0 mol%) were dissolved in 5/2/1 (*v*/*v*) toluene/water/methanol (10 mL) under argon atmosphere. The mixture was refluxed at 100 °C overnight. The reaction was quenched with water. The organic phase was extracted with ethyl acetate. The combined organic layers were dried over MgSO_4_, filtered and evaporated under reduced pressure. The residue was chromatographed with silica gel by eluting with 3/1 (*v*/*v*) hexane/dichloromethane. Orange solid. Yield: 0.42 g (1.26 mmol), 55%; ^1^H-NMR (500 MHz, CDCl_3_) δ 7.88–7.85 (m, 3H, Ar**H**), 7.51 (d, *J* = 7.6 Hz, 1H, Ar**H**), 6.86 (td, *J* = 2.5, 9.9 Hz, 2H, Ar**H**), 3.05 (s, 6H, NC**H**_3_) ppm (Figure S46).

**4-(7-(4-heptylphenyl)benzo[c][1,2,5]thiadiazol-4-yl)-*N,N*-dimethylaniline (Me_2_NC7)**

4-(7-bromobenzo[c][1,2,5]thiadiazol-4-yl)-N,N-dimethylaniline (0.11 g, 0.28 mmol), (4-heptylphenyl)boronic acid (0.14 g, 0.42 mmol), K_3_PO_4_ (0.18 g, 0.84 mmol), and Pd(PPh_3_)_4_ (0.01 g, 0.01 mmol) were dissolved in 5/2/1 (*v*/*v*) toluene/water/methanol (10 mL). The reaction was quenched with water. The organic phase was extracted with ethyl acetate. The combined organic layers were dried over MgSO_4_, filtered and evaporated under reduced pressure. The residue was chromatographed with silica gel by eluting 3/2 (*v*/*v*) hexane/dichloromethane. Orange solid. Yield: 0.06 g (0.14 mmol), 50%; ^1^H-NMR (500 MHz, CDCl_3_) δ 7.93 (td, *J* = 2.6, 9.9 Hz, 2H, Ar**H**), 7.87 (td, *J* = 2.9, 8.4 Hz, 2H, Ar**H**), 7.74 (d, *J* = 7.3 Hz, 1H, Ar**H**), 7.71 (d, *J* = 7.3 Hz, 1H, Ar**H**), 7.35 (d, *J* = 8.2 Hz, 2H, Ar**H**), 6.90 (td, *J* = 2.4, 9.9 Hz, 2H, Ar**H**), 3.05 (s, 6H, NC**H**_3_), 2.69 (t, *J* = 7.6 Hz, 2H, C**H**_2_), 1.72–1.66 (m, 2H, C**H**_2_), 1.41–1.25 (m, 8H, C**H**_2_), 0.90 (t, *J* = 7.0 Hz, 3H, C**H**_3_) ppm (Figure S55). ^13^C-NMR (126 MHz, CDCl_3_) δ 154.5, 150.7, 143.2, 135.2, 133.4, 131.9, 130.2, 129.1, 128.8, 128.2, 126.5, 125.5, 112.5, 40.6, 36.0, 32.0, 31.6, 29.5, 29.4, 22.8, 14.3 ppm (Figure S56), FT-IR (Figure S85); HRMS (EI) Calcd for C_27_H_31_N_3_S: 429.2239, Found 429.2246 (Figure S100).

## 4. Conclusions

We synthesized and characterized a series of BTD-based terphenyl-like fluorescent LCs functionalized with various donor and acceptor groups. Most of the synthesized compounds exhibited supercooled nematic phases at room temperature. In contrast, TFMeC8 and C6NTFMe, which contain trifluoromethyl groups, exhibited smectic phases. All the derivatives showed fluorescence ranging from green to red, depending on the functional groups. The amine-functionalized derivatives (Me_2_NC8, Me_2_NOC8, and EHNOC6) exhibited red fluorescence in THF and red or orange fluorescence in the solid state, with relatively high Φ_fl_ values. Meanwhile, C6NCN and C6NTFMe also exhibited red fluorescence but had significantly lower Φ_fl_ values, likely owing to strong intermolecular interactions causing fluorescence quenching. The acceptor-substituted derivatives (AldC8, ActC8, TFActC8, and TFMeC8) exhibited green fluorescence with varying Φ_fl_ values (0.28–0.99).

The fluorescence properties in the mesophase were also examined, which revealed that the *λ*_fl_ values were generally redshifted compared with the solid state, suggesting differences in aggregation structures and luminescent species. Although most derivatives exhibited lower Φ_fl_ values (~0.10) in the mesophase due to enhanced molecular motion at elevated temperatures, the smectic LC TFMeC8 maintained a high Φ_fl_, likely because of its layered structure, which more closely resembles the crystalline phase than the nematic phase.

Overall, these findings highlight the versatility of BTD-based mesogenic skeletons for designing room-temperature fluorescent nematic LCs with various functional groups, including unfavorable groups for calamitic nematic LCs. While the relationship between molecular structure and fluorescence behavior in the solid and solution states has been relatively well characterized, the photophysical properties in the mesophase require further investigation. In particular, understanding how molecular packing, aggregation structures, and dynamic effects influence fluorescence efficiency in the mesophase remains a key challenge. Our future research will focus on these factors, as well as the optimization of molecular design strategies to achieve enhanced fluorescence efficiency and stability in mesophases. The findings will provide valuable guidance for the design of advanced materials for fluorescent LCs.

## Figures and Tables

**Table 1:** Phase transition behavior of BTD derivatives, determined by DSC measurement at a rate of 10 °C min^−1^ upon 3rd heating and cooling.

Entry	Phase Transition Behavior [°C] (Δ*H* [kJ mol^−1^])	
	Heating	Cooling
Me_2_NC7	Cry 108.1 (29.0) Iso	Iso 106.8 (−0.5) N 41.7 (−17.7) Cry
Me_2_NC8	N 68.0 (−2.7) Cry 87.6 (3.8) N 100.6 (0.5) Iso	Iso 98.4 (−0.6) N
Me_2_NOC7	Cry 122.8 (42.2) N 135.7 (0.5) Iso	Iso 133.7 (−0.6) N 77.3 (−32.5) Cry
Me_2_NOC8	N 22.4 (−7.7) Cry_1_ 36.3 (−9.4) Cry_2_ 114.2, 120.0 (35.2) Iso	Iso 120.3 (−0.70) N
Me_2_NOEH	N 34.8 (−26.3) Cry_1_ 80.4 (33.0) Cry_2_ 97.3 (3.4) Iso	Iso 32.0 (−1.0) N
C8MeNC7	Iso	Iso
C6NOC6	N 14.3 (−5.60) Cr_1_ 48.4 (−5.80) Cr_2_ 80.4 (26.0) N 144.6 (0.97) Iso	Iso 142.6 (−0.97) N
EHNOC6	N 50.7 (0.02) Iso	Iso 47.8 (−0.04) N
EHNOEH	Iso	Iso
AldC8	N 90.8 (0.48) Iso	Iso 89.1 (−0.76) N
ActC8	N 58.2 (0.19) N_1_ 66.4 (0.03) N_2_ 75.6 (0.16) N_3_ 102.6 (0.48) Iso	Iso 100.7 (−0.54) N
TFActC8	N 35.7 (0.2) Iso	Iso 33.3 (−0.2) N
TFMeC8	Cry 74.7 (19.1) Iso	Iso 66.9 (−3.2) SmA 48.6 (−13.1) Cry
C6NCN	Cry 163.2 (37.7) Iso	Iso 154.2 (−0.37) N 143.6 (−36.4) Cry
C6NTFMe ^[a]^	SmA 80 Iso	Iso 72-55 SmA

^[a]^ Phase transition behavior was determined through POM.

**Table 2:** Photophysical properties of representative derivatives in THF solution, mesophase, solid state.

Entry	THF				Mesophase		Solid	
	*ε*^a^ (L mol^−1^ cm^−1^)	*λ*_abs_^b^ (nm)	*λ*_em_^c^ (nm)	Φ_flu_ (-)	*λ*_em_ (nm)	Φ_flu_ (-)	*λ*_em_ (nm)	Φ_flu_ (-)
Me_2_NC8	35,000 12,000	302 451	661	>0.99 0.86	647	0.36	592	0.80
Me_2_NOC8	31,000 12,000	305 454	657	0.67 0.57	652	0.28	658	0.56
EHNOC6	38,000 14,000	305 459	650	0.55 0.77	640	0.06	591	0.50
AldC8	27,000 15,000	304 389	493	0.56 >0.99	513	0.12	502	0.50
ActC8	27,000 15,000	296 389	494	0.70 >0.99	519	0.13	490	0.66
TFActC8	20,000 18,000	310 391	495	0.53 >0.99	522	0.09	505	0.28
TFMeC8	24,000 11,000	279 384	493	0.30 >0.99	519	0.56	502	0.59
C6NCN	28,000 13,000	313 465	674	0.44 0.52	631	--	648	0.08
C6NTFMe	28,000 12,000	301 456	667	0.21 0.41	652	0.03	643	0.08

^a^ molar absorption coefficient. ^b^ maximum absorption wavelength. ^c^ maximum fluorescence wavelength.

## Data Availability

The original contributions presented in the study are included in the article/Supplementary Materials, further inquiries can be directed to the corresponding author.

## Supplementary Materials

The following supporting information can be downloaded at: https://www.mdpi.com/article/10.3390/molecules30112438/s1, Figures S1–S13: DSC thermogram; Figures S14–S26: POM images; Figures S27–S35: Absorption and fluorescence spectra; Figures S36–S84: NMR spectra; Figures S85–S99: FT-IR spectra; Figures S100–S112: HRMS spectra; Figure S113 and S114: WAXD images; Scheme S1–S5: Synthesis of BTD-based LCs.

## References

[1] Hoeben F.J.M., Jonkheijm P., Meijer E.W., Schenning A.P.H.J. (2005). About Supramolecular Assemblies of π-Conjugated Systems. Chem. Rev..

[2] Fleischmann E.-K., Zentel R. (2013). Liquid-Crystalline Ordering as a Concept in Materials Science: From Semiconductors to Stimuli-Responsive Devices. Angew. Chem. Int. Ed..

[3] Niori T., Sekine T., Watanabe J., Furukawa T., Takezoe H. (1996). Distinct ferroelectric smectic liquid crystals consisting of banana shaped achiral molecules. J. Mater. Chem..

[4] Imrie C.T., Henderson P.A. (2007). Liquid crystal dimers and higher oligomers: Between monomers and polymers, Chem. Soc. Rev..

[5] Swager T.M. (2022). Molecular Shape and Polar Order in Columnar Liquid Crystals. Acc. Chem. Res..

[6] O’Neill M., Kelly S.M. (2010). Ordered Materials for Organic Electronics and Photonics. Adv. Mater..

[7] O’Neill M., Kelly S.M. (2003). Liquid crystals for charge transport, luminescence, and photonics. Adv. Mater..

[8] Kumar M., Kumar S. (2017). Liquid Crystals in Photovoltaics: A New Generation of Organic Photovoltaics. Polym. J..

[9] Hwang J., Song M.H., Park B., Nishimura S., Toyooka T., Wu J.W., Takanishi Y., Ishikawa K., Takezoe H. (2005). Electro-tunable optical diode based on photonic bandgap liquid-crystal heterojunctions. Nat. Mater..

[10] Kobashi J., Yoshida H., Ozaki M. (2016). Planar optics with patterned chiral liquid crystals. Nat. Photonics.

[11] Padalkar V.S., Tsutsui Y., Sakurai T., Sakamaki D., Tohnai N., Kato K., Takata M., Akutagawa T., Sakai K., Seki S. (2017). Optical and structural properties of ESIPT inspired HBT–fluorene molecular aggregates and liquid crystals. J. Phys. Chem. B.

[12] Takahashi K., Taguchi D., Kajitani T., Fukushima T., Kubo S., Shishido A. (2023). Synthesis and Characterization of Side-Chain Liquid-Crystalline Block Copolymers Containing Cyano-Terminated Phenyl Benzoate Moieties. Molecules.

[13] Zhang W., Sakurai T., Aotani M., Watanabe G., Yoshida H., Padalkar V.S., Tsutsui Y., Sakamaki D., Ozaki M., Seki S. (2019). Highly fluorescent liquid crystals from excited-state intramolecular proton transfer molecules. Adv. Opti. Mater..

[14] Shigeyama T., Matsumoto K., Hisano K., Tsutsumi O. (2023). Tunable Reflection through Size Polydispersity of Chiral-Nematic Liquid Crystal Polymer Particles. Molecules.

[15] Uchimura M., Watanabe Y., Araoka F., Watanabe J., Takezoe H., Konishi G. (2010). Development of Laser Dyes to Realize Low Threshold in Dye-Doped Cholesteric Liquid Crystal Lasers. Adv. Mater..

[16] Oladepo S.A. (2022). Development and Application of Liquid Crystals as Stimuli-Responsive Sensors. Molecules.

[17] Wang L., Urbas L.M., Li Q. (2020). Nature-Inspired Emerging Chiral Liquid Crystal Nanostructures: From Molecular Self-Assembly to DNA Mesophase and Nanocolloids. Adv. Mater..

[18] Gao J., Tang Y., Martella D., Guo J., Wiersma D.S., Li Q. (2023). Stimuli-responsive photonic actuators for integrated biomimetic and intelligent systems. Responsive Mater..

[19] Kang W., Tang Y., Meng X., Lin S., Zhang X., Guo J., Li Q. (2023). A Photo- and Thermo-Driven Azoarene-Based Circularly Polarized Luminescence Molecular Switch in a Liquid Crystal Host. Angew. Chem..

[20] Bisoyi H.K., Li Q. (2022). Liquid Crystals: Versatile Self-Organized Smart Soft Materials. Chem. Rev..

[21] De Castro L.D.C., Lub J., Oliveira O.N., Schenning A.P.H.J. (2025). Mechanochromic Displays Based on Photoswitchable Cholesteric Liquid Crystal Elastomers. Angew. Chem. Int. Ed..

[22] Fellert M., Hein R., Ryabchun A., Gisbert Y., Stindt C.N., Feringa B.L. (2025). A Multiresponsive Ferrocene-Based Chiral Overcrowded Alkene Twisting Liquid Crystals. Angew. Chem. Int. Ed..

[23] Shoji Y., Komiyama R., Kobayashi M., Kosaka A., Kajitani T., Haruki R., Kumai R., Adachi S.I., Tada T., Karasawa N. (2023). Collective bending motion of a two-dimensionally correlated bowl-stacked columnar liquid crystalline assembly under a shear force. Sci. Adv..

[24] Yagai S., Okamura S., Nakano Y., Yamauchi M., Kishikawa K., Karatsu T., Kitamura A., Ueno A., Kuzuhara D., Yamada H. (2014). Design amphiphilic dipolar π-systems for stimuli-responsive luminescent materials using metastable states. Nat. Commun..

[25] Strachan G.J., Górecka E., Hobbs J., Pociecha D. (2025). Fluorination: Simple Change but Complex Impact on Ferroelectric Nematic and Smectic Liquid Crystal Phases. J. Am. Chem. Soc..

[26] Qiao S., Liao R., Xie M., Song X., Zhang A., Fang Y., Zhang C., Yu H. (2025). Synthesis and Optoelectronic Properties of Perylene Diimide-Based Liquid Crystals. Molecules.

[27] Li B., Zhang J., Wang J., Chen X. (2024). Aggregation-Induced Emission-Active Cyanostilbene-Based Liquid Crystals: Self-Assembly, Photophysical Property, and Multiresponsive Behavior. Molecules.

[28] Hang Z., Jiang S., Wu Z., Gong J., Zhang L. (2024). A Novel Near-Infrared Tricyanofuran-Based Fluorophore Probe for Polarity Detection and LD Imaging. Molecules.

[29] Bui T.-T., Péralta S., Dumur F. (2023). Synthesis and Optical Properties of a Series of Push-Pull Dyes Based on Pyrene as the Electron Donor. Molecules.

[30] Singh A.P., Gupta S.K., Mohiuddin G., Khan R.K., Karunakar M. (2024). Synthesis, structure and mesogenic properties of benzoxazole derivatives. Liq. Cryst..

[31] Da Silva F.N., Silva E.O., dos Santos G., Postacchini B.B., Cazati T., Bechtold I.H., Vieira A.A. (2023). Unlocking the potential of 2,1,3-benzoxadiazole-based luminescent liquid crystals. Liq. Cryst..

[32] Cruickshank E. (2024). The Emergence of a Polar Nematic Phase: A Chemist’s Insight into the Ferroelectric Nematic Phase. ChemPlusChem.

[33] Mandle R.J., Sebastián N., Martinez-Perdiguero J., Mertelj A. (2021). On the molecular origins of the ferroelectric splay nematic phase. Nat. Commun..

[34] Chen X., Korblova E., Dong D., Wei X., Shao R., Radzihovsky L., Glaser M.A., Maclennan J.E., Bedrov D., Walba D.M. (2020). First-principles experimental demonstration of ferroelectricity in a thermotropic nematic liquid crystal: Polar domains and striking electro-optics. Proc. Natl. Acad. Sci. USA.

[35] Nishikawa H., Okada D., Kwaria D., Nihonyanagi A., Kuwayama M., Hoshino M., Araoka F. (2024). Emergent Ferroelectric Nematic and Heliconical Ferroelectric Nematic States in an Achiral “Straight” Polar Rod Mesogen. Adv. Mater..

[36] Czerwiński M., Dmochowska E., Herman J., Filipow M., Biełaga G., Lubiński N., Perkowski P., Kula P. (2024). Chiral liquid crystal dimers with smectic phases for stabilization of anticlinic state in surface-stabilised geometry. J. Mol. Liq..

[37] Arakawa Y., Sasaki Y., Haraguchi N., Itsuno S., Tsuji H. (2017). Synthesis, phase transitions and birefringence of novel liquid crystalline 1,4-phenylene bis(4-alkylthio benzoates) and insights into the cybotactic nematic behaviour. Liq. Cryst..

[38] Arakawa Y., Kang S., Tsuji H., Watanabe J., Konishi G. (2016). The design of liquid crystalline bistolane-based materials with extremely high birefringence. RSC Adv..

[39] Arakawa Y., Kang S., Tsuji H., Watanabe J., Konishi G. (2016). Development of novel bistolane-based liquid crystalline molecules with an alkylsulfanyl group for highly birefringent materials. RSC Adv..

[40] Arakawa Y., Sasaki S., Igawa K., Tokita M., Konishi G., Tsuji H. (2020). Birefringence and photoluminescence properties of diphenylacetylene-based liquid crystal dimers. New J. Chem..

[41] Long G.Y., Deng Y.P., Zhao W., Zhou G.F., Broer D.J., Feringa B., Chen J.W. (2024). Photoresponsive Biomimetic Functions by Light-Driven Molecular Motors in Three Dimensionally Printed Liquid Crystal Elastomers. J. Am. Chem. Soc..

[42] Jones B.E., Greenfield J.L., Cowieson N., Fuchter M.J., Evans R.C. (2024). Light-Driven Hexagonal-to-Cubic Phase Switching in Arylazopyrazole Lyotropic Liquid Crystals. J. Am. Chem. Soc..

[43] Sahoo D., Peterca M., Percec V. (2024). Designing Highly Ordered Helical and Nonhelical Porous Crystalline and Disordered Nonhelical Columnar Liquid Crystalline Self-Organizations. J. Am. Chem. Soc..

[44] Zhang Y., Wang X., Yang W., Yan H., Zhang X., Han D., He Y., Li C., Sun L. (2023). Programmable Complex Shape Changing of Polysiloxane Main-Chain Liquid Crystalline Elastomers. Molecules.

[45] Shaya J., Ribierre J.-C., Correia G., Dappe Y.J., Mathevet F., Mager L., Heinrich B., Méry S. (2023). Control of the Organization of 4,4′-bis(carbazole)-1,1′-biphenyl (CBP) Molecular Materials through Siloxane Functionalization. Molecules.

[46] Zhou L., Zheng L.L., Gao M.Y., Xu C.J., Ge Y.F., Bai T.X., Wen J., Cheng Y.H., Zhu M.F. (2023). Confinement fluorescence effect of an aggregation-induced emission luminogen in crystalline polymer. Aggregate.

[47] Anders C., Tan T., Fischer V.M., Ruoyu W., Alaasar M., Waldecker R., Cao Y., Liu F., Tschierske C. (2025). Engineering “Meso-Atom” bonding: Honeycomb-network transitions in reticular liquid crystals. Aggregate.

[48] Sakurai T., Kato K., Shimizu M. (2023). Side-Chain Labeling Strategy for Forming Self-Sorted Columnar Liquid Crystals from Binary Discotic Systems. Crystals.

[49] Wu X., Niu X., Zhu S., Tian M., Liu W. (2023). A novel multi-stimuli responsive fluorescence liquid crystal material with aggregationinduced emission effect. Liq. Cryst..

[50] Sawatari Y., Shimomura Y., Takeuchi M., Iwai R., Tanaka T., Tsurumaki E., Tokita M., Watanabe J., Konishi G. (2024). Supramolecular liquid crystals from the dimer of L-shaped molecules with tertiary amide end groups. Aggregate.

[51] Shimomura Y., Kawamura A., Tokita M., Watanabe J., Konishi G. (2023). Fluorinated Poly(pentylene 4,4′-bibenzoate)s with Low Isotropization Temperatures and Unique Phase Transition Behavior. Macromolecules.

[52] Kang S., Nakajima S., Arakawa Y., Tokita M., Watanabe J., Konishi G. (2014). Highly birefringent side-chain LC polymethacrylate with a dinaphthyl-acetylene mesogenic unit. Polym. Chem..

[53] Voskuhl J., Giese M. (2022). Mesogens with aggregation-induced emission properties: Materials with a bright future. Aggregate.

[54] Zhu Y., Zeng S., Li B., McEllin A.J., Liao J., Fang Z., Xiao C., Bruce D.W., Zhu W., Wang Y. (2022). Liquid-Crystalline Thermally Activated Delayed Fluorescence: Design, Synthesis, and Application in Solution-Processed Organic Light-Emitting Diodes. ACS Appl. Mater. Interfaces.

[55] Chen S., Li Y., Guo Z., Ma T., Duan Y., Yang Y., Cheng X. (2024). Fluorescent Columnar Liquid Crystals Based on BTD Hexacatenars with Salicylaldimine and Benzaldimine Core: Self-Assembly and Multifunctional Properties. J. Mol. Liq..

[56] Ye Q., Zhu D., Zhang H., Lu X., Lu Q. (2015). Thermally Tunable Circular Dichroism and Circularly Polarized Luminescence of Tetraphenylethene with Two Cholesterol Pendants. J. Mater. Chem. C.

[57] Chen Y., Li Y., Liang Y., Liu X., Xiao Y. (2023). The Effect of the Number of Cyanostilbene Building Blocks on the Self-Assembly and Photophysical Property of Pyrene-Based Liquid Crystals. Tetrahedron.

[58] Dhingra S., Gupta S.P., Shah A., Singh D.P., Pal S.K. (2024). Temperature-Dependent Hole Mobility in Pyrene–Thiophene-Based Room-Temperature Discotic Liquid Crystals. Chem. Commun..

[59] Irla S., Pruthvi M., Raghunathan V.A., Kumar S. (2021). Design and Synthesis of Extended Pyrene Based Discotic Liquid Crystalline Dyes. Dyes Pigment..

[60] Sagara Y., Yamane S., Mutai T., Araki K., Kato T. (2009). A Stimuli-Responsive, Photoluminescent, Anthracene-Based Liquid Crystal: Emission Color Determined by Thermal and Mechanical Processes. Adv. Func. Mater..

[61] Venkatesh S., Yadav P.K., Hasija D.C., Patil V.R., Ramana M.M.V. (2020). Novel Blue-Light Emitting Fluorescent Liquid Crystals Based on 4, 4′-(2-(Tert-Butyl)- Anthracene-9, 10-Diyl)Diphenol and Their Optical Behavior. J. Mol. Liq..

[62] Wang Y., Fang D., Fu T., Ali M.U., Shi Y., He Y., Hu Z., Yan C., Mei Z., Meng H. (2020). Anthracene Derivative Based Multifunctional Liquid Crystal Materials for Optoelectronic Devices. Mater. Chem. Front..

[63] Giménez R., Piñol M., Serrano J.L. (2004). Luminescent liquid crystals derived from 9,10-bis(phenylethynyl)anthracene. Chem. Mater..

[64] Zhang M., Bu X., Liu T., Guo W., Wang H., Zeng Z. (2018). Adjustable 2-Cyano-3,4-Difluoro-1H-Pyrrole-Based Luminescent Liquid Crystals: Synthesis, Properties and Substituent Effect. J. Mol. Liq..

[65] Demus D., Goodby J., Gray G.W., Spiess H.W., Vill V. (1999). Physical Properties of Liquid Crystals.

[66] Heilmeier G.H., Zanoni L.A., Barton L.A. (1968). Guest-host interactions in nematic liquid crystals. A new electro-optic effect. Appl. Phys. Lett..

[67] Heilmeier G.H. (1976). Liquid crystal displays: An experiment in interdisciplinary research that worked. IEEE Trans Electron Devices.

[68] Demus D. (2006). One Century Liquid Crystal Chemistry: From Vorländer’s Rods to Disks, Stars and Dendrites. Mol. Cryst. Liq. Cryst..

[69] Iwai R., Yoshida H., Arakawa Y., Sasaki S., Iida Y., Igawa K., Sakurai T., Suzuki S., Tokita M., Watanabe J. (2024). Near-room-temperature π-conjugated nematic liquid crystals in molecules with a flexible seven-membered ring structure. Aggregate.

[70] Shimomura Y., Iida Y., Tsurumaki E., Konishi G. (2025). Innovative Molecular Design of Bridged Biphenyls for Calamitic Nematic Liquid Crystals with Extensive π-Conjugated Mesogens. Mater. Chem. Front..

[71] Konishi G., Sawatari Y., Iwai R., Tanaka T., Shimomura Y., Tokita M. (2024). Synthesis of Side-Chain Liquid Crystalline Polyacrylates with Bridged Stilbene Mesogens. Molecules.

[72] Iida Y., Shimomura Y., Tokita M., Konishi G. (2024). Push-Pull Biphenyl and Tolane Derivatives as Novel Luminescent Liquid Crystals: Synthesis and Properties. Liq. Cryst..

[73] Vieira A.A., Cristiano R., Bortoluzzi A.J., Gallardo H. (2008). Luminescent 2,1,3-Benzothiadiazole-Based Liquid Crystalline Compounds. J. Mol. Struct..

[74] Aguiar L.d.O., Regis E., Tuzimoto P., Girotto E., Bechtold I.H., Gallardo H., Vieira A.A. (2018). Investigation of Thermal and Luminescent Properties in 4,7-Diphenylethynyl-2,1,3-Benzothiadiazole Systems. Liq. Cryst..

[75] Sonar P., Singh S.P., Leclère P., Surin M., Lazzaroni R., Lin T.T., Dodabalapur A., Sellinger A. (2009). Synthesis, Characterization and Comparative Study of Thiophene–Benzothiadiazole Based Donor–Acceptor–Donor (D–A–D) Materials. J. Mater. Chem..

[76] Echeverri M., Martín I., Concellón A., Ruiz C., Anselmo M.S., Gutiérrez-Puebla E., Serrano J.L., Gómez-Lor B. (2018). Fluorescent and Electroactive Monoalkyl BTD-Based Liquid Crystals with Tunable Self-Assembling and Electronic Properties. ACS Omega.

[77] Hori A., Matsumoto A., Ikenouchi J., Konishi G. (2025). D−π–A Fluorophores with Strong Solvatochromism for Single-Molecule Ratiometric Thermometers. J. Am. Chem. Soc..

[78] Miyaura N., Suzuki A. (1995). Palladium-Catalyzed Cross-Coupling Reactions of Organoboron Compounds. Chem. Rev..

[79] Tanaka T., Matsumoto A., Klymchenko A.S., Tsurumaki E., Ikenouchi J., Konishi G. (2024). Fluorescent Solvatochromic Probes for Long-Term Imaging of Lipid Order in Living Cells. Adv. Sci..

[80] Rybczyński P., Bousquet M.H.E., Kaczmarek-Kędziera A., Jędrzejewska B., Jacquemin D., Ośmiałowski B. (2022). Controlling the Fluorescence Quantum Yields of Benzothiazole-Difluoroborates by Optimal Substitution. Chem. Sci..

[81] Benevides T.O., Regis E., Nicoleti C.R., Bechtold I.H., Vieira A.A. (2019). Phase-Dependent Photoluminescence of Non-Symmetric 2,1,3-Benzothiadiazole Liquid Crystals. Dye. Pigment..

[82] Wang J.-L., Xiao Q., Pei J. (2010). Benzothiadiazole-Based D−π-A−π-D Organic Dyes with Tunable Band Gap: Synthesis and Photophysical Properties. Org. Lett..

[83] Ishi-i T., Tanaka H., Youfu R., Aizawa N., Yasuda T., Kato S., Matsumoto T. (2019). Mechanochromic Fluorescence Based on a Combination of Acceptor and Bulky Donor Moieties: Tuning Emission Color and Regulating Emission Change Direction. New J. Chem..

[84] Gierschner J., Shi J., Milián-Medina B., Roca-Sanjuán D., Varghese S., Park S. (2021). Luminescence in Crystalline Organic Materials: From Molecules to Molecular Solids. Adv. Opt. Mater..

[85] Shimomura Y., Konishi G. (2023). Push-Pull Bridged Distyrylbenzene with Highly Bright Solid-State Red-Orange Aggregation-Induced Emission. Chem. Eur. J..

[86] Shimomura Y., Konishi G. (2022). Flexible Alkylene Bridges as a Tool To Engineer Crystal Distyrylbenzene Structures Enabling Highly Fluorescent Monomeric Emission. Chem. Eur. J..

[87] Iwasaki T., Murakami S., Takeda Y., Fukuhara G., Tohnai N., Yakiyama Y., Sakurai H., Kambe N. (2019). Molecular Packing and Solid-State Photophysical Properties of 1,3,6,8-Tetraalkylpyrenes. Chem. Eur. J..

[88] Mandle R.J., Bevis E., Goodby J.W. (2014). Phase Structures of Nematic Liquid Crystals. Handbook of Liquid Crystals.

[89] Belmonte-Vázquez J.L., Amador-Sánchez Y.A., Rodríguez-Cortés L.A., Rodríguez-Molina B. (2021). Dual-State Emission (DSE) in Organic Fluorophores: Design and Applications. Chem. Mater..

