# Supplementary material for: Development of 2,1,3-Benzothiadiazole-Based Room-Temperature Fluorescent Nematic Liquid Crystals
Source: Molecules. 2025 Jun 2;30(11):2438. doi: 10.3390/molecules30112438 (PMC12155834; doi:10.3390/molecules30112438)
Supplement: Supplementary file 1 [file molecules-30-02438-s001.zip › molecules-3588960-supplementary.pdf]

Development of 2,1,3-Benzothiadiazole-based Room-temperature Fluorescent Nematic Liquid Crystals

Muhammad Suhail bin Uzair[1], Yoshimichi Shimomura[1], Takuya Tanaka[1], Takashi Kajitani[2], Gen-ichi Konishi[1*]

[1]Department of Chemical Science and Engineering, Institute of Science Tokyo, Tokyo 152-8552, Japan

[2]Core Facility Center, Research Infrastructure Management Center, Institute of Science Tokyo, Yokohama 226-0026, Japan

*Correspondence: konishi.g.aa@m.titech.ac.jp

Table of contents

General method and materials 2

Differential scanning calorimetry (DSC) thermograms 3

Polarized Optical Microscopy (POM) images 10

Fluorescence properties 16

Experimental section 21

Spectra chart 35

NMR chart 35

FT-IR spectra 52

High-resolution mass spectrometry (HRMS) chart 60

General method and materials

Characterization

^{1}H-NMR and ^{13}C-NMR spectra were recorded on BRUKER Ascend 500 (125 MHz) spectrometers for $CDCl_3$ solution using tetramethylsilane (TMS) as an internal standard. ^{1}H-NMR spectra were reported as follows: chemical shift (δ ppm), multiplicity (s = singlet, d = doublet, t = triplet, q = quartet, m = multiplet), integration, and coupling constants in units of Hz. ^{13}C-NMR spectra were reported as chemical shifts in ppm. The FT-IR spectra were recorded on a JASCO FT-IR 4600 spectrometer. HRMS. Polarized optical microscopy (POM) was performed using Olympus BX51 optical microscope with a Mettler FP90 hot stage at a rate of 10 ºC min^{-1}. Differential scanning calorimetry (DSC) was performed using PerkinElmer DSC 8500 equipment at a scanning rate of 10 °C min^{-1} under a flow of dry nitrogen. Wide-angle X-ray diffraction (WAXD) patterns were obtained using a Rigaku Nano-Viewer equipped with a HyPix-3000 detector and Cu Kα radiation. Column chromatography was carried out with Kanto Chemical silica gel 60 N (63–210 mesh). TLC was carried out with Merck Silica Gel 60 F254 (0.2 mm) plate.

Optical measurements

UV-Vis spectra were recorded on a JASCO V-670 UV-vis spectrophotometer. Fluorescence spectra and absolute quantum yields were measured by Edinburgh fluorescence spectrometer FS5. All photophysical measurements were performed using dilute solutions with optical densities (ODs) around 0.1 at the maximum absorption wavelength in 1 cm path length quartz cells at room temperature (298 K). In addition, all sample solutions were de-aerated by bubbling with argon gas for 15 min prior to the quantum yield.

Materials

Unless noted otherwise, all reagents and chemical were received without further purification. 4-bromophenol, 4-bromoaniline, 4-bromo-*N*-methylaniline, 4-bromo-*N,N*-dimethylaniline, 1-bromo-4-*n*-heptylbenzene, 1-bromo-4-*n*-octylbenzene, 4,7-dibromobenzo[c][1,2,5]thiadiazole, 1-iodooctane, 1-bromo-2-ethylhexane, 4'-bromo-2,2,2-trifluoroacetophenone, 4-(trifluoromethyl)phenylboronic acid, 4-cyanophenylboronic acid, potassium carbonate (K_2CO_3), Tetrakis(triphenylphosphine)palladium (0) ($Pd(PPh_3)_4$, $Pd(dppf)Cl_2{\cdot}CH_2Cl_2$, bis(pinacolato)diboron and 2.3M *n*-butyllithium were obtained from TCI (Tokyo, Japan). 1-bromohexane, 1-bromoheptane, 1-bromooctane, 4-acetylphenylboronic acid, 4-formylphenylboronic acid, potassium iodide (KI), potassium acetate (AcOK), hydrochloric acid, 2-butanone, *N,N*-dimethylformamide (DMF) and tetrahydrofuran for spectrochemical analysis were obtained from Wako Pure Chem (Tokyo, Japan). Potassium phosphate (K_3PO_4), tetrahydrofuran, 1,4-dioxane, and toluene were obtained from Kanto Chemical (Tokyo, Japan). Methanol, ethyl acetate and hexane were obtained from GODO (Tokyo, Japan). Dichloromethane was obtained from AGC (Tokyo, Japan).

Phase transition behavior

Differential scanning calorimetry (DSC) thermograms

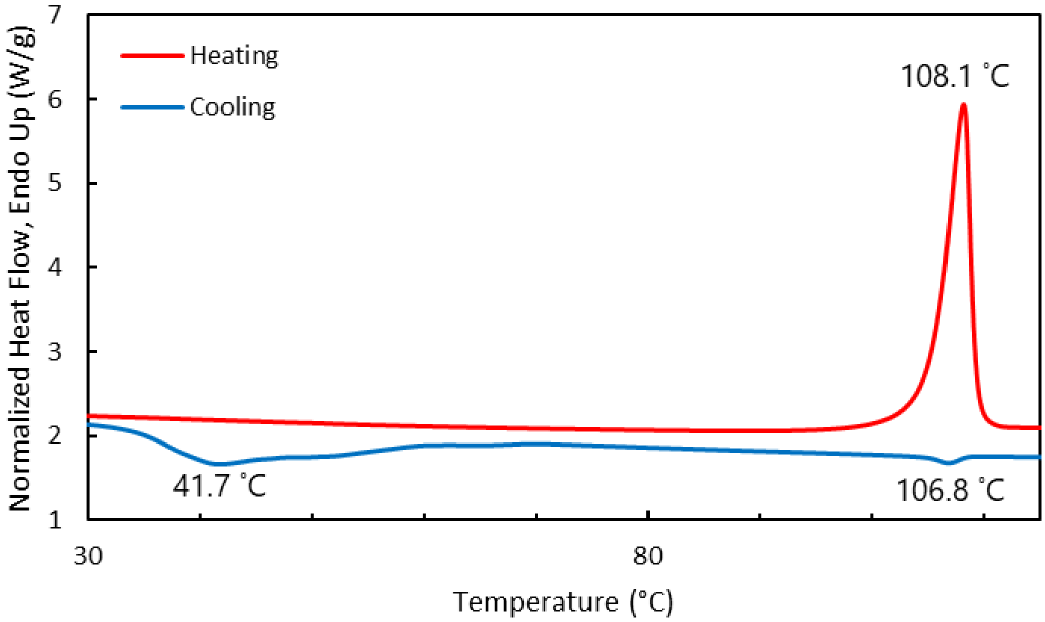

Figure S1. DSC thermogram of **Me$_2$NC7** at a heating or cooling rate of 10 °C min^{-1} (2nd cycle).

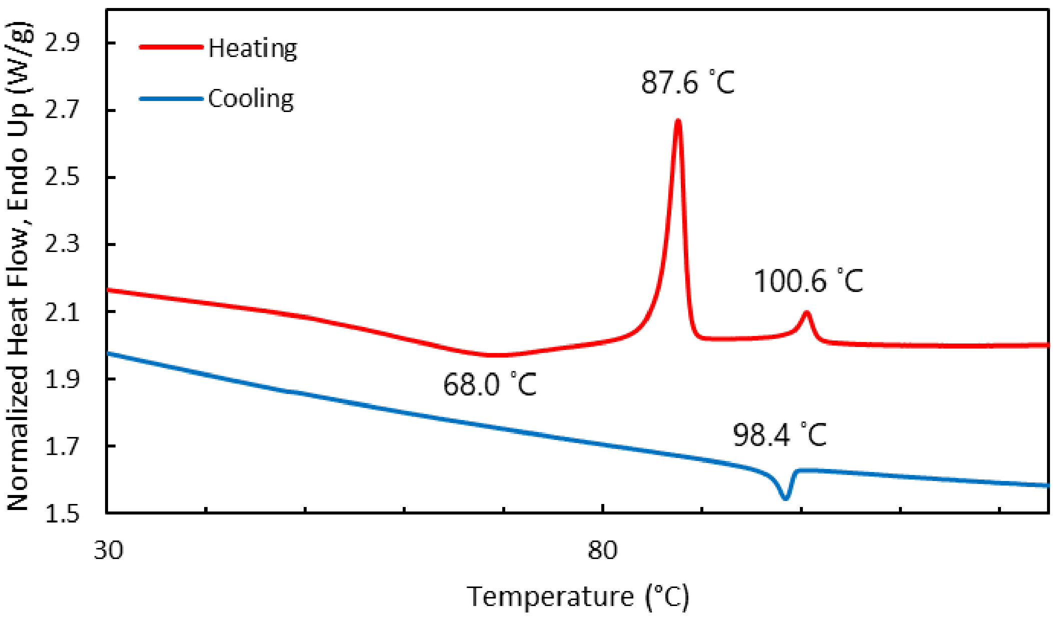

Figure S2. DSC thermogram of **Me$_2$NC8** at a heating or cooling rate of 10 °C min^{-1} (2nd cycle).

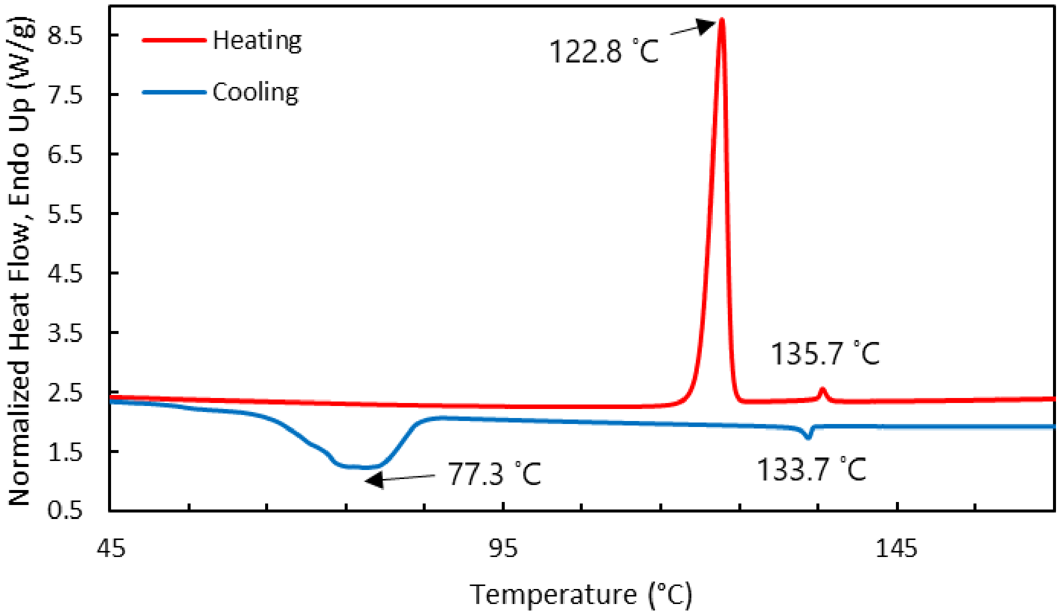

Figure S3. DSC thermogram of **Me$_2$NOC7** at a heating or cooling rate of 10 °C min^{-1} (2nd cycle).

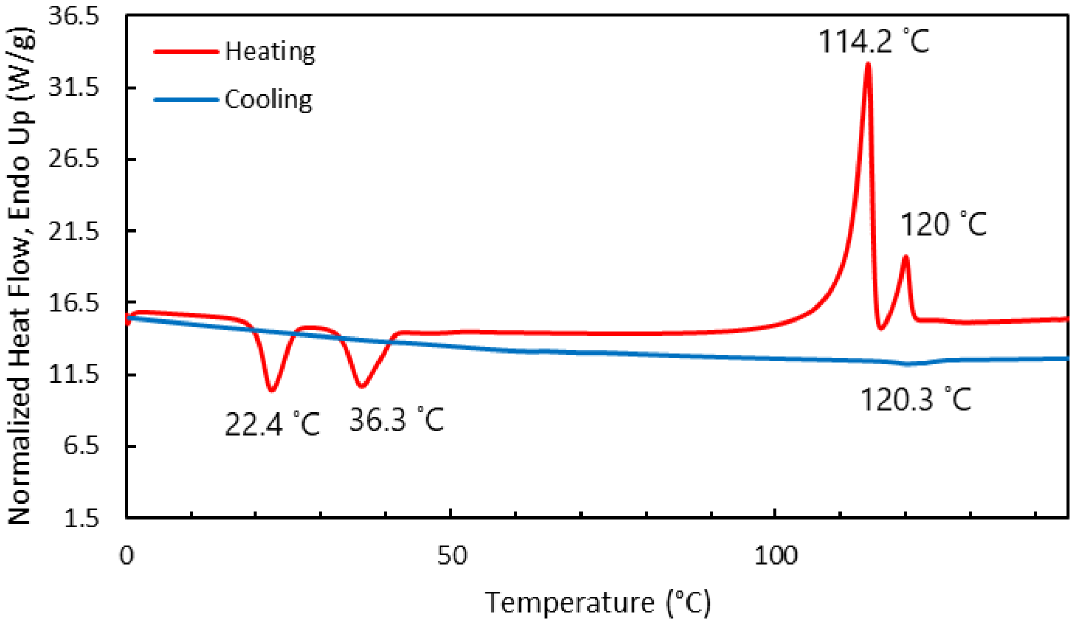

Figure S4. DSC thermogram of **Me$_2$NOC8** at a heating or cooling rate of 10 °C min^{-1} (2nd cycle).

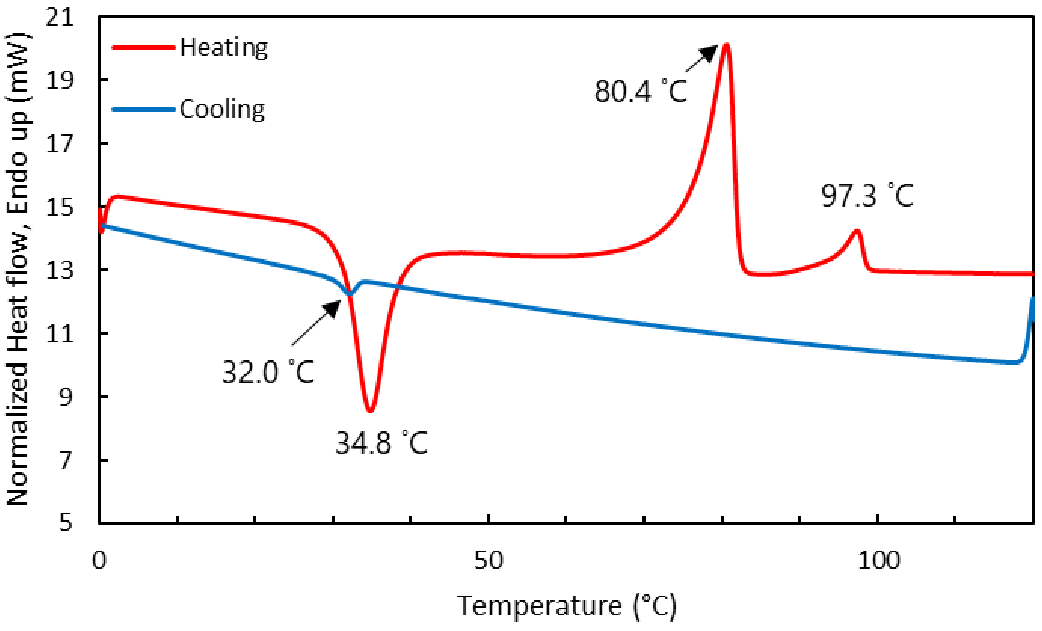

Figure S5. DSC thermogram of **Me_2NOEH** at a heating or cooling rate of 10 °C min^{-1} (2nd cycle).

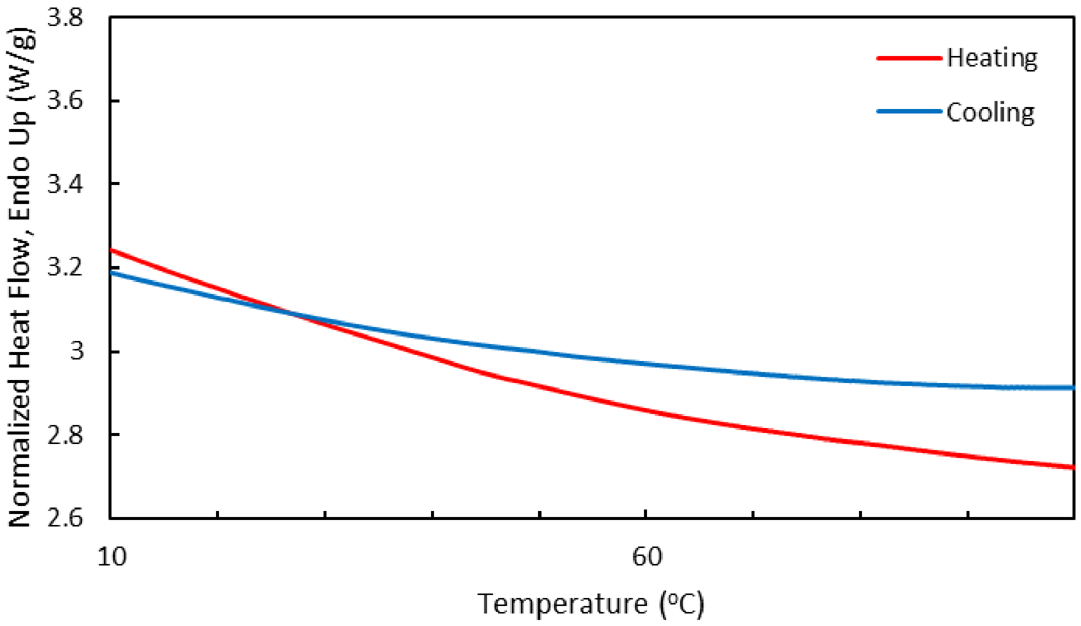

Figure S6. DSC thermogram of **C8MeNC7** at a heating or cooling rate of 10 °C min^{-1} (2nd cycle).

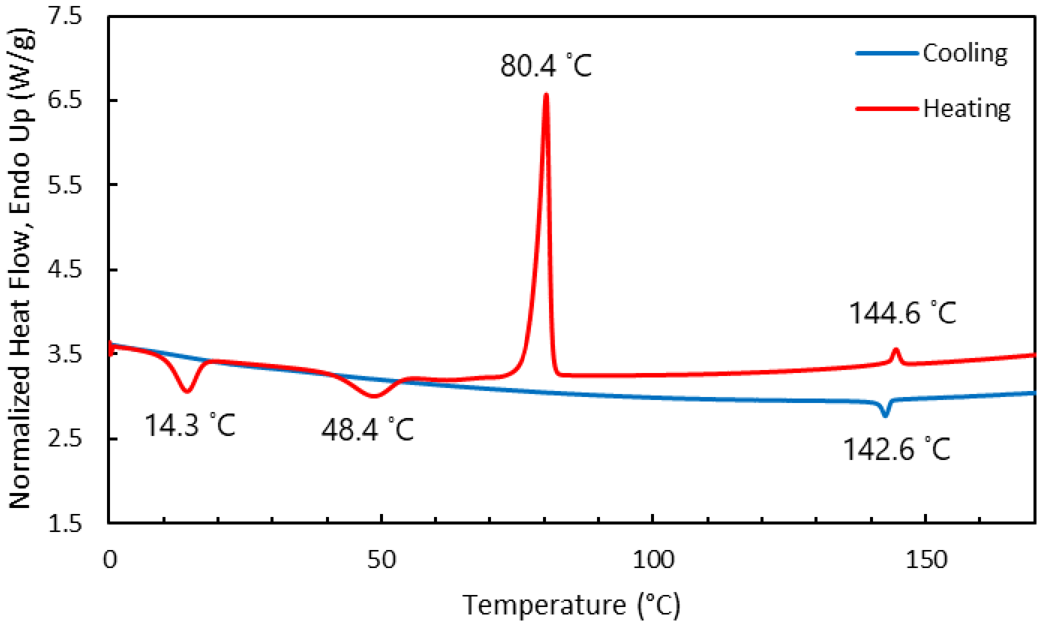

Figure S7. DSC thermogram of **C6NOC6** at a heating or cooling rate of 10 °C min^{-1} (2nd cycle).

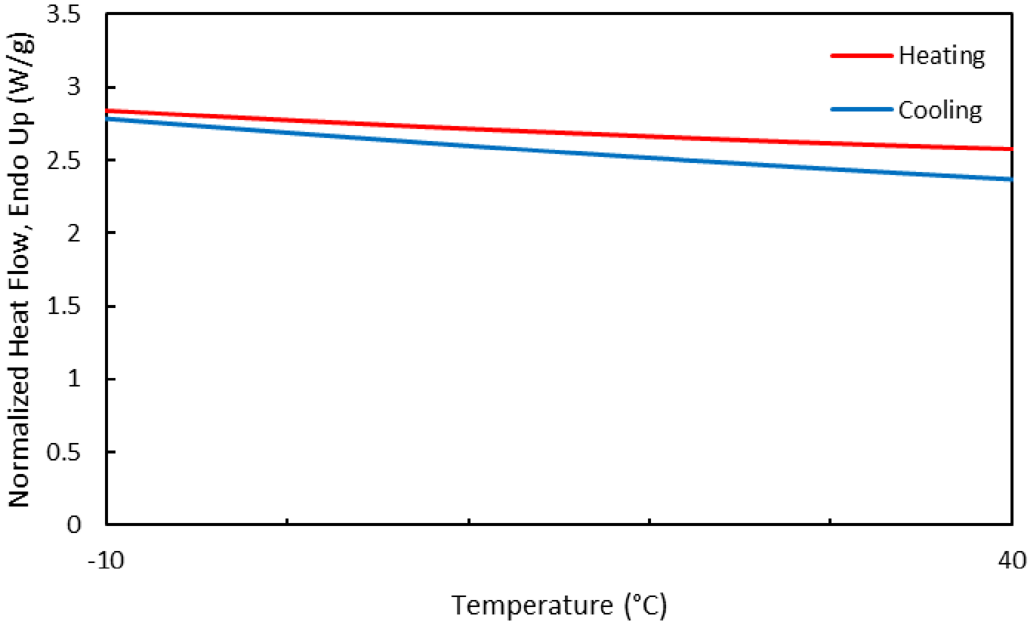

Figure S8. DSC thermogram of **EHNOEH** at a heating or cooling rate of 10 °C min^{-1} (2nd cycle).

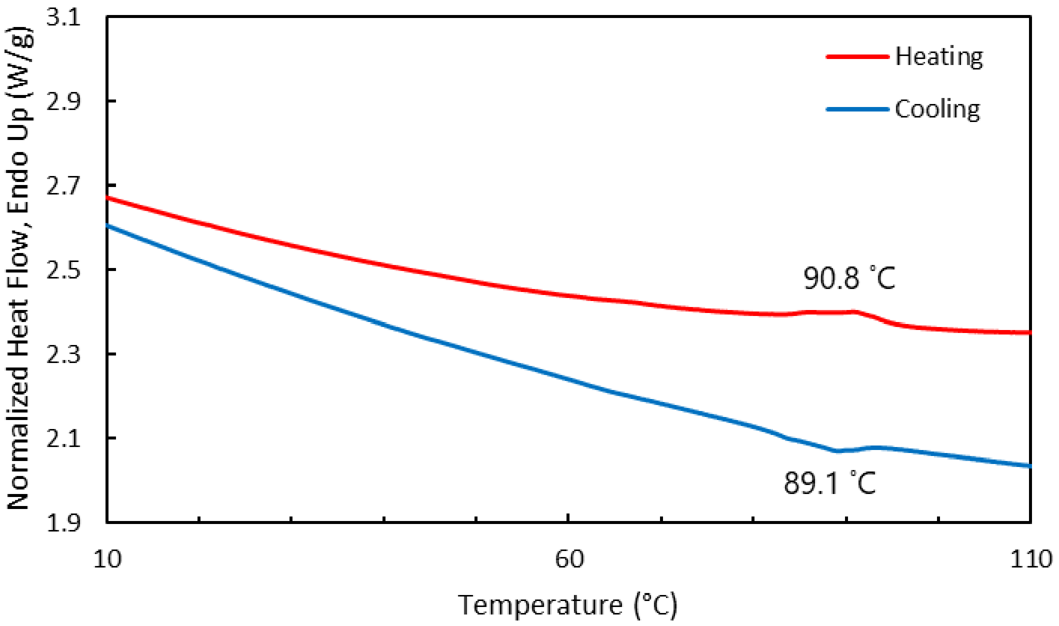

Figure S9. DSC thermogram of **AldC8** at a heating or cooling rate of 10 °C min^{-1} (2nd cycle).

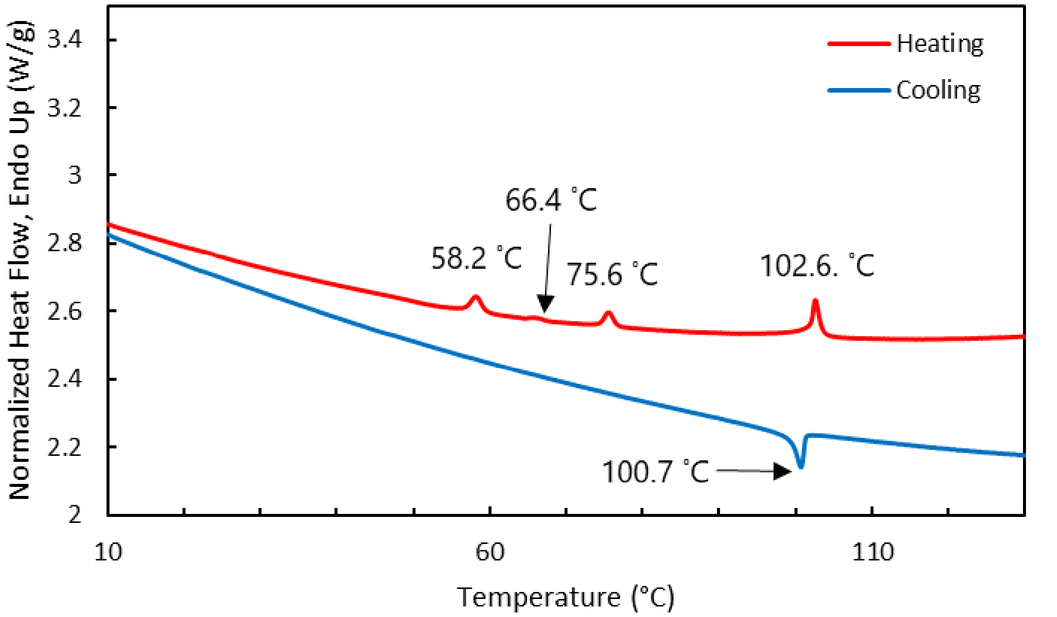

Figure S10. DSC thermogram of **ActC8** at a heating or cooling rate of 10 °C min^{-1} (2nd cycle).

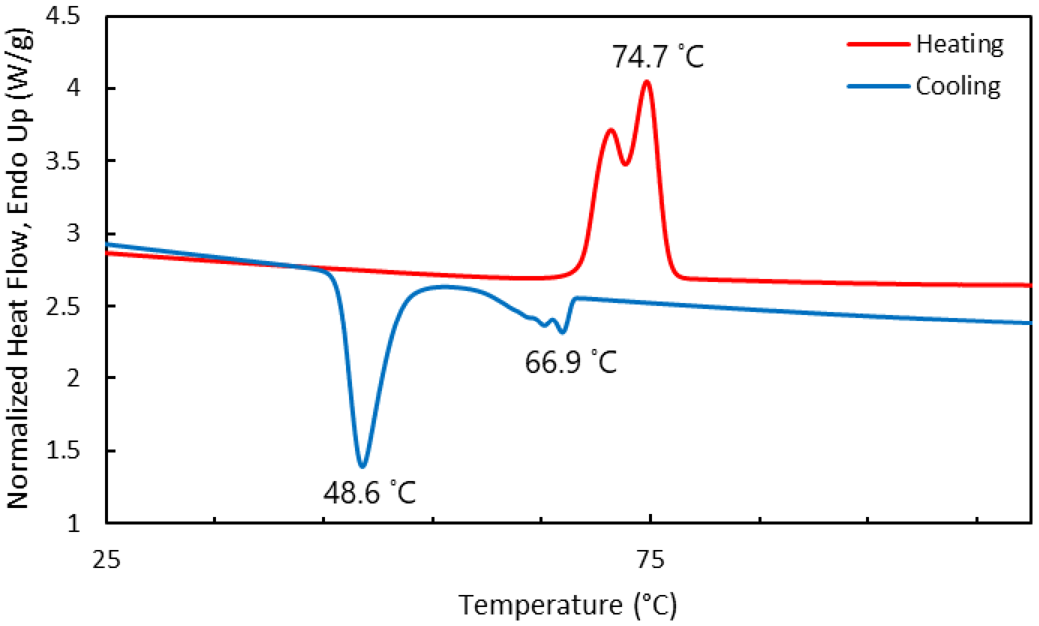

Figure S11. DSC thermogram of **TFMeC8** at a heating or cooling rate of 10 °C min^{-1} (2nd cycle).

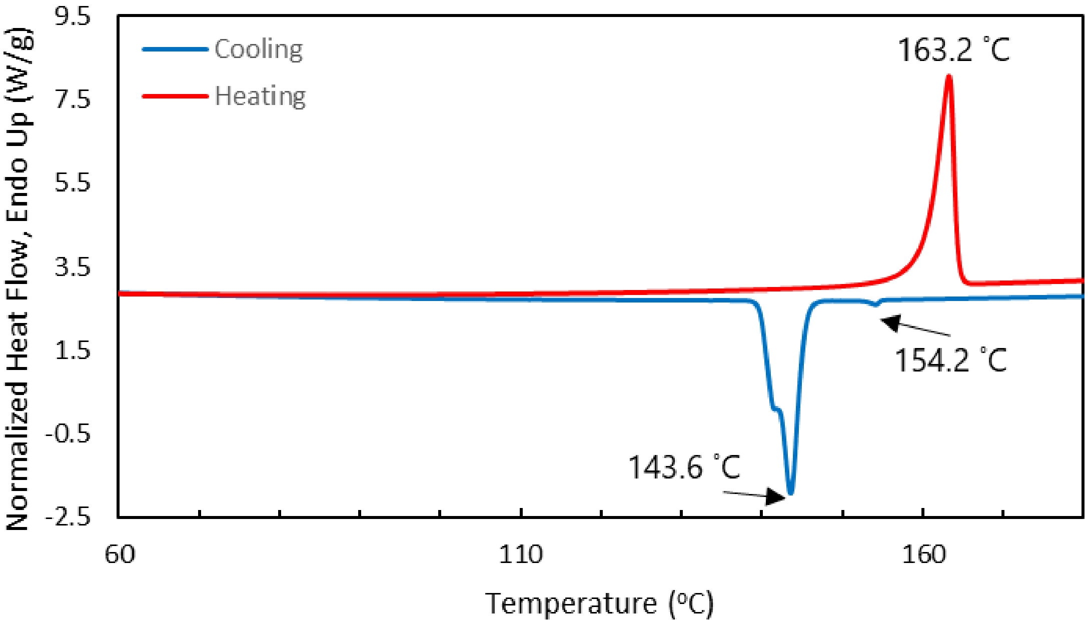

Figure S12. DSC thermogram of **C6NCN** at a heating or cooling rate of 10 °C min^{-1} (2nd cycle).

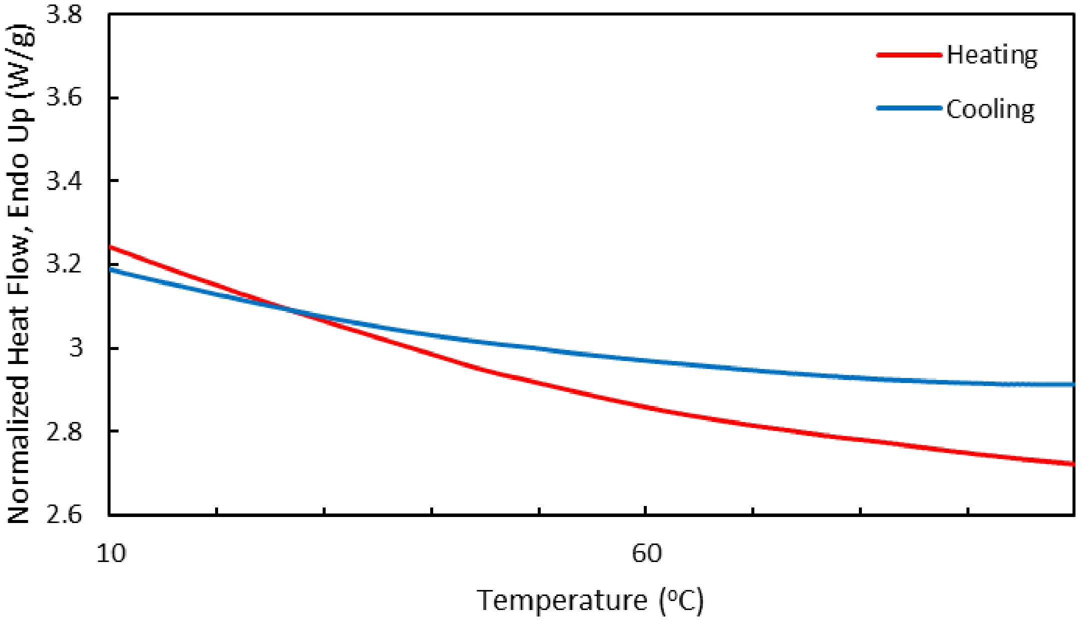

Figure S13. DSC thermogram of **C6NTFMe** at a heating or cooling rate of 10 °C min^{-1} (2nd cycle).

Polarized Optical Microscopy (POM) images

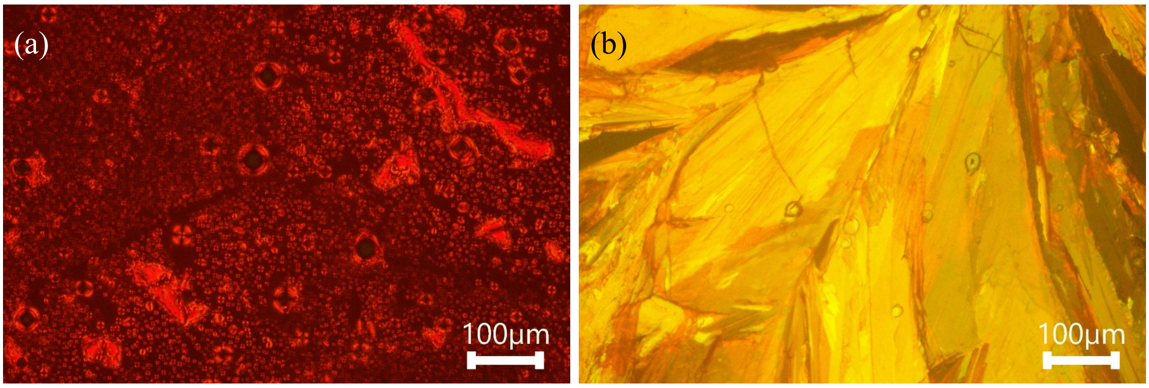

Figure S14. POM images of **Me$_2$NC7** at (a) 94 °C in the nematic (N) phase upon cooling, and (b) room temperature in the crystalline (Cr) phase.

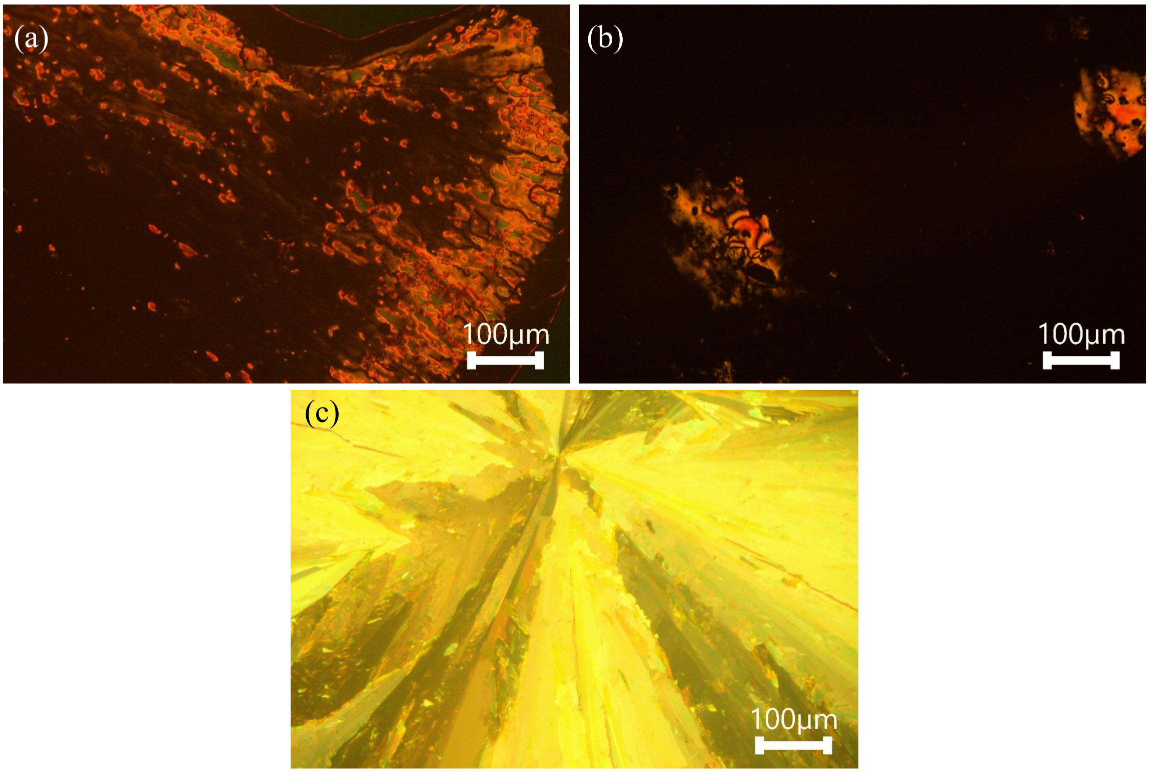

Figure S15. POM images of **Me$_2$NC8** at (a) 90 °C in the N phase upon heating, (b) 90 °C in the N phase upon cooling, and (c) in the Cr phase after being left overnight at room temperature.

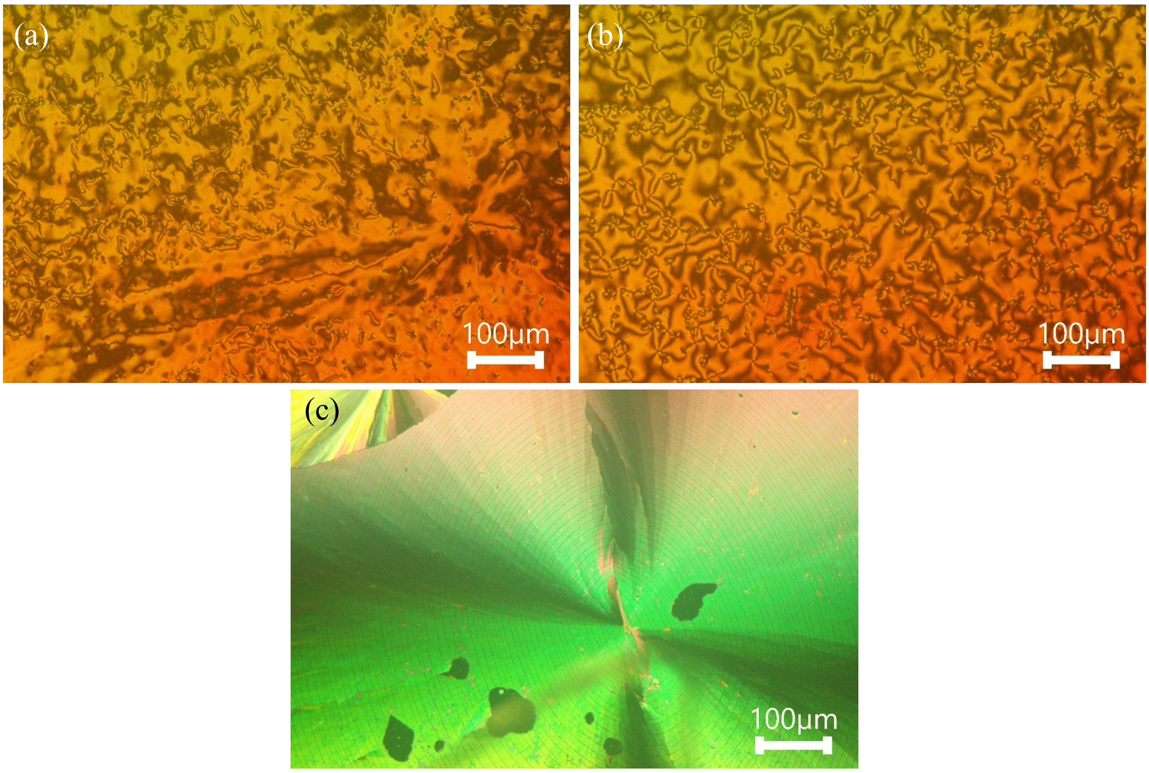

Figure S16. POM images of **Me$_2$NOC7** at (a) 123 °C in the N phase upon heating, (b) 118 °C in the N phase upon cooling, and (c) room temperature in the Cr phase.

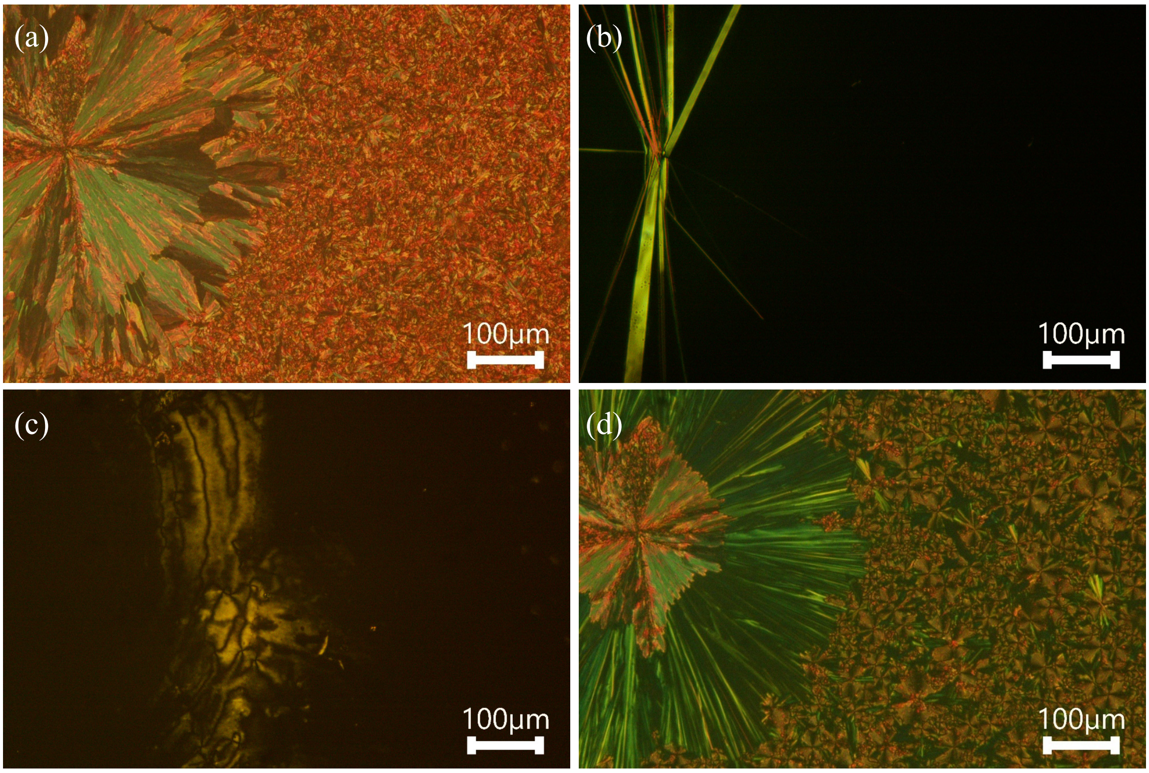

Figure S17. POM images of **Me$_2$NOC8** at (a) 60 °C in the Cr phase, (b) 115 °C in the Cr phase upon heating, (c) 96 °C in the N phase, and (d) room temperature in the Cr phase.

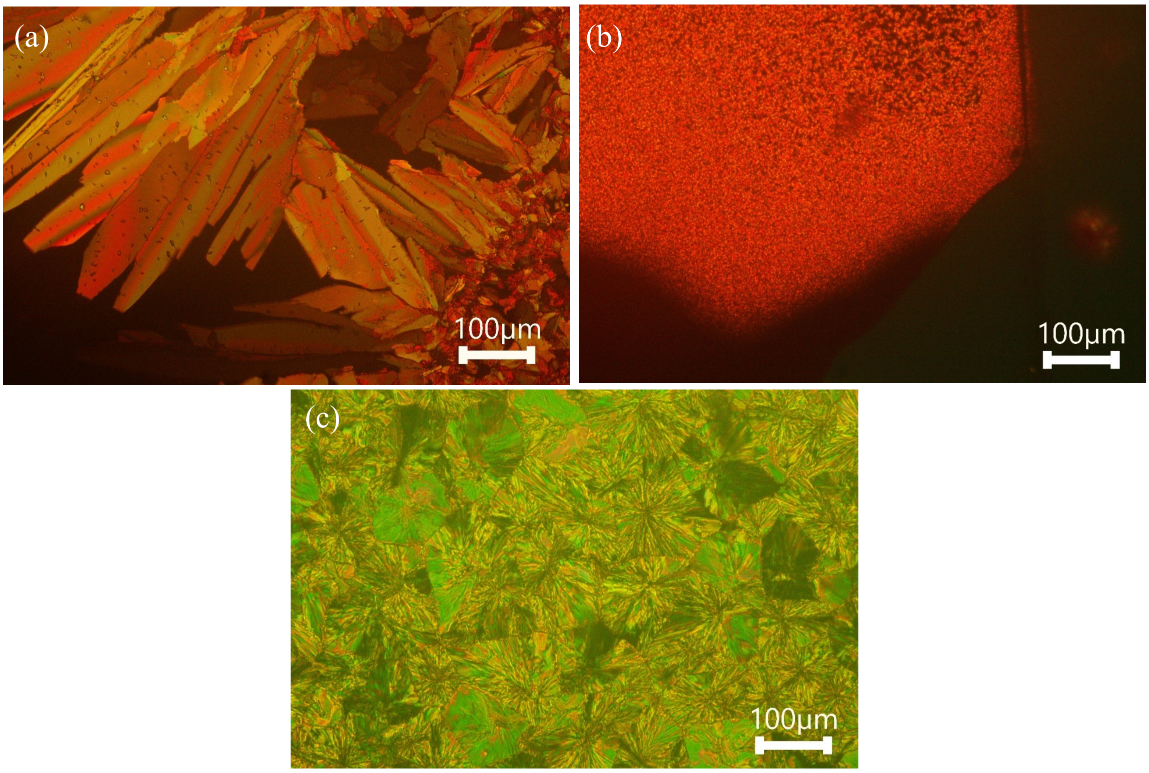

Figure S18. POM images of **Me_2NOEH** at (a) 90 °C in the Cr phase applied under stress upon heating, (b) 34 °C in the N phase upon cooling, and (b) in the Cr phase after being left overnight at room temperature.

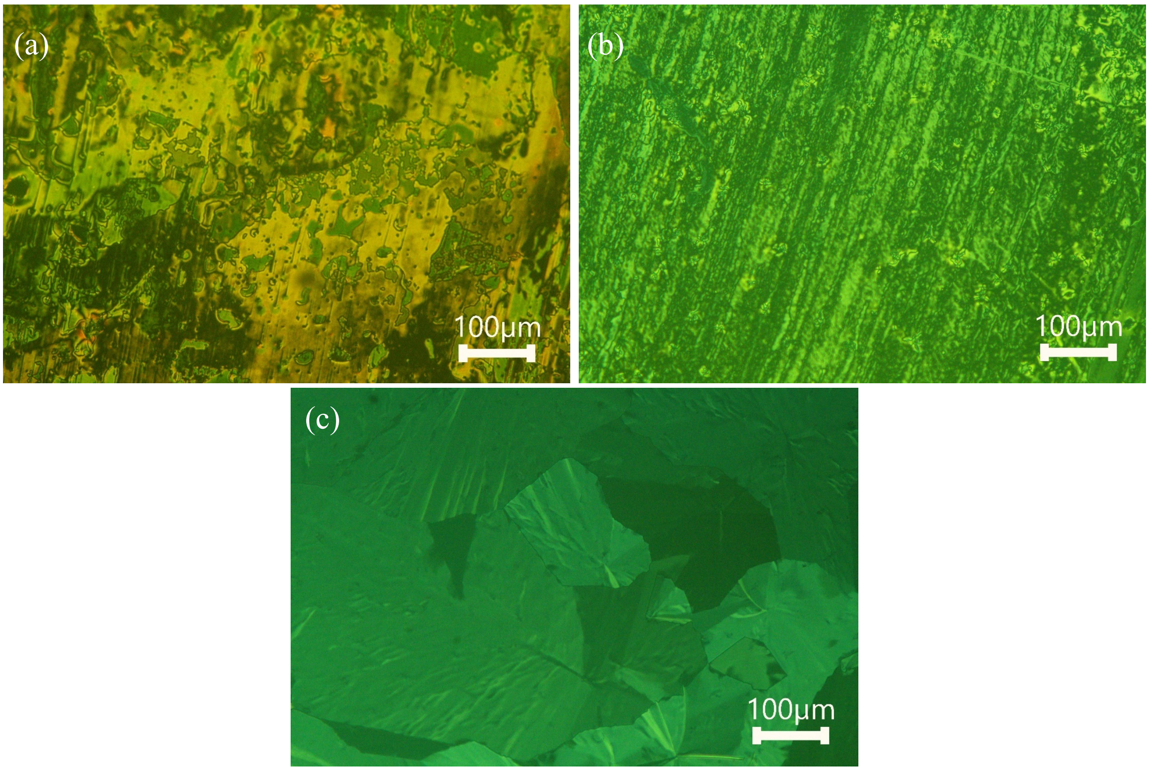

Figure S19. POM images of **C6NOC6** at (a) 98 °C in the N phase upon heating, (b) 77 °C in the N phase upon cooling, and (c) room temperature in the Cr phase.

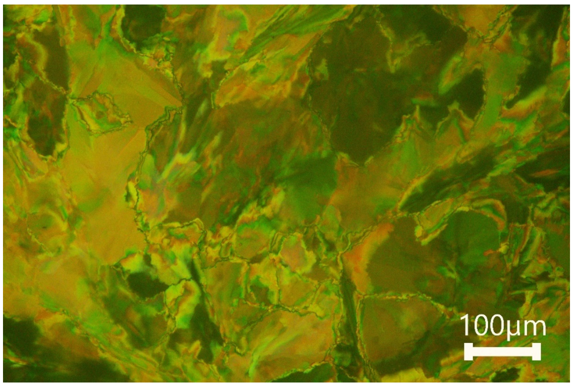

Figure S20. A POM image of **EHNOC6** in the Cr phase after being left overnight at room temperature.

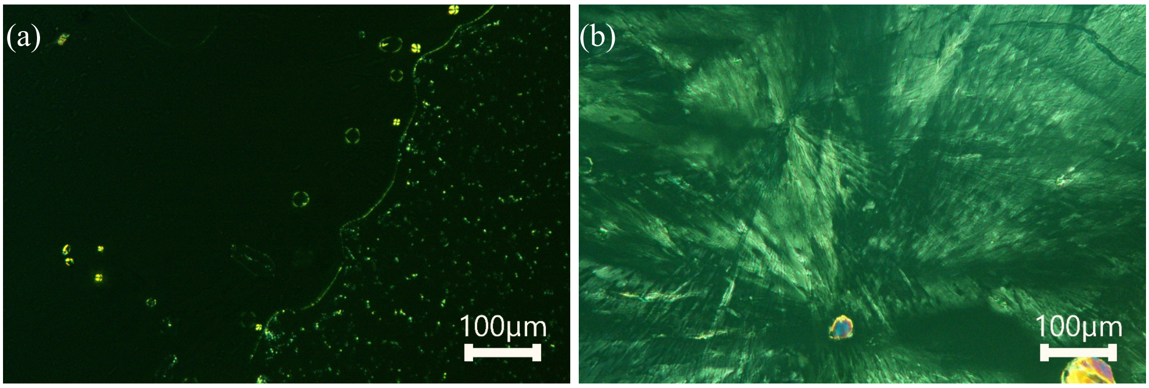

Figure S21. POM images of **AldC8** at (a) 65 °C in the N phase upon cooling, and (b) in the Cr phase after being left for 2 days at room temperature.

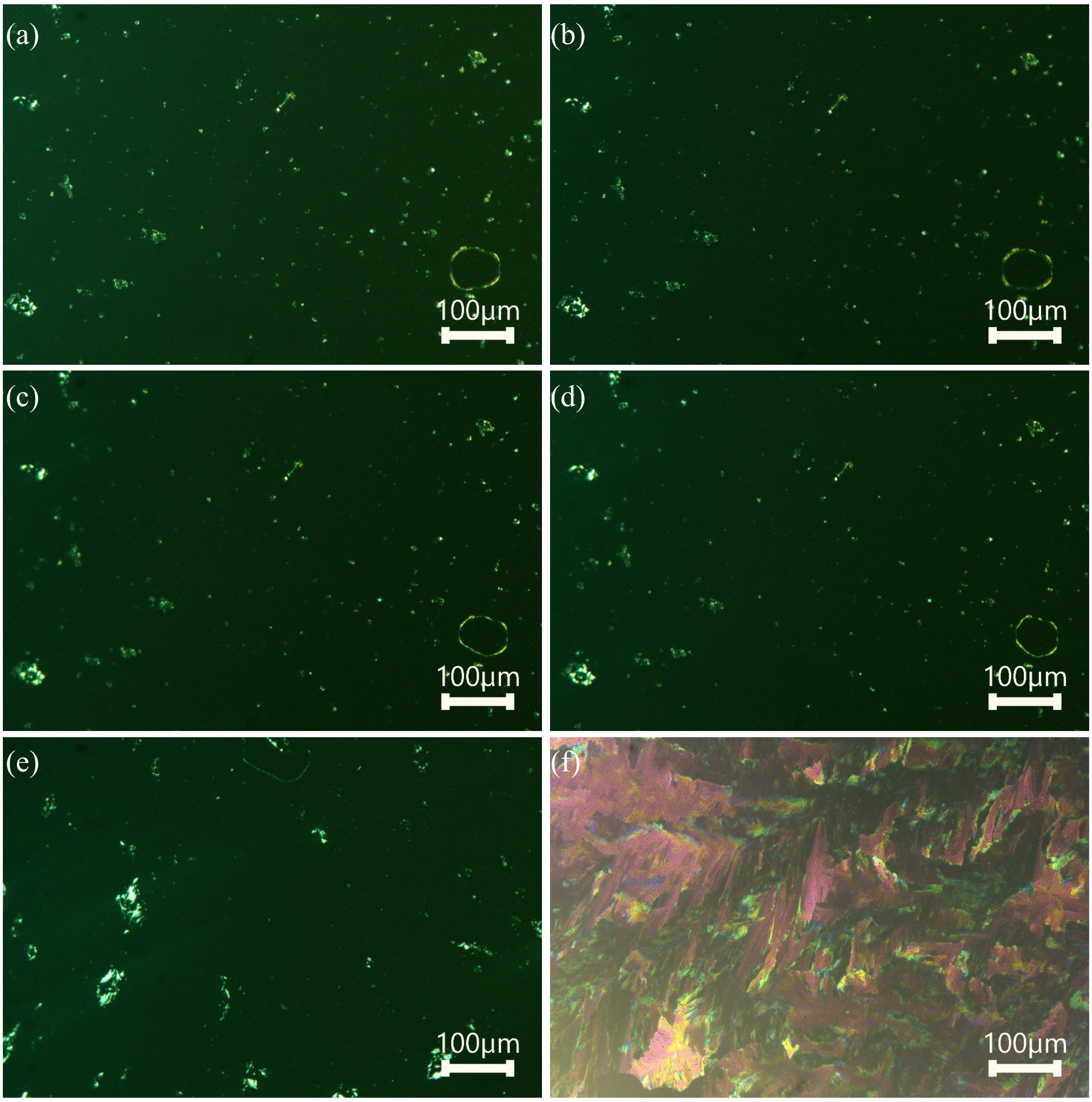

Figure S22. POM images of **ActC8** at (a) 43 °C, (b) 61 °C, (c) 71 °C, (d) 80 °C in the N phase upon heating, (e) 93 °C in the N phase upon cooling, and (f) in the Cr phase after being left overnight at room temperature.

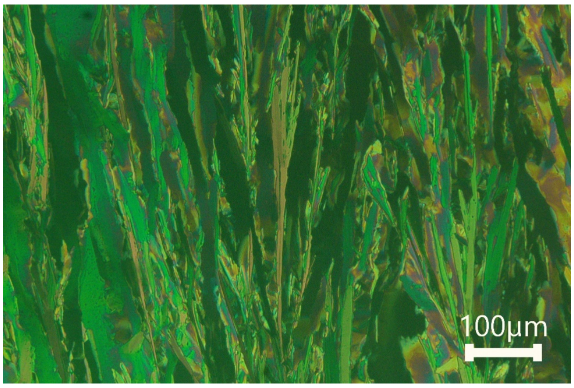

Figure S23. A POM image of **TFActC8** in the Cr phase after being left for a week at room temperature.

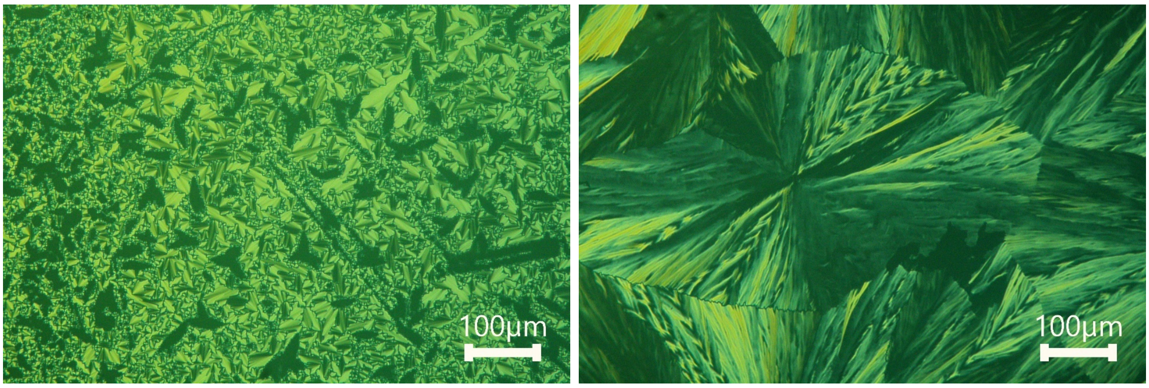

Figure S24. POM images of **TFMeC8** at (a) 60 °C in the smectic A (SmA) phase, and (b) at 40 °C in the Cr phase upon cooling.

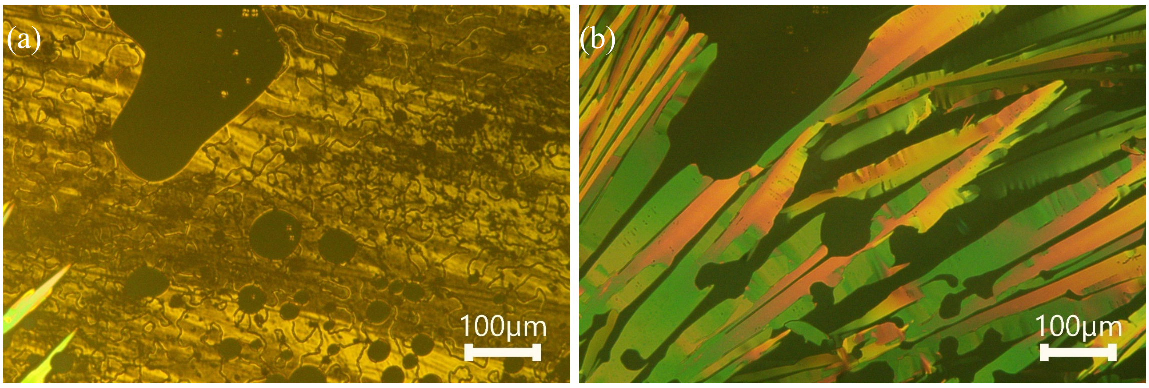

Figure S25. POM images of **C6NCN** at (a) 143 °C in the N phase, and (b) at 120 °C in the Cr phase upon cooling.

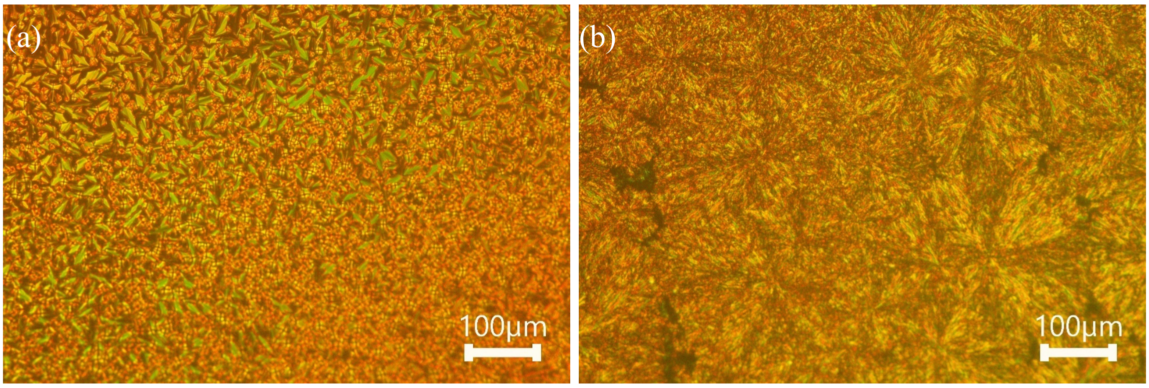

Figure S26. POM images of **C6NTFMe** at (a) 50 °C in the SmA phase upon cooling, and (b) in the Cr phase after being left for a week at room temperature.

Photophysical properties

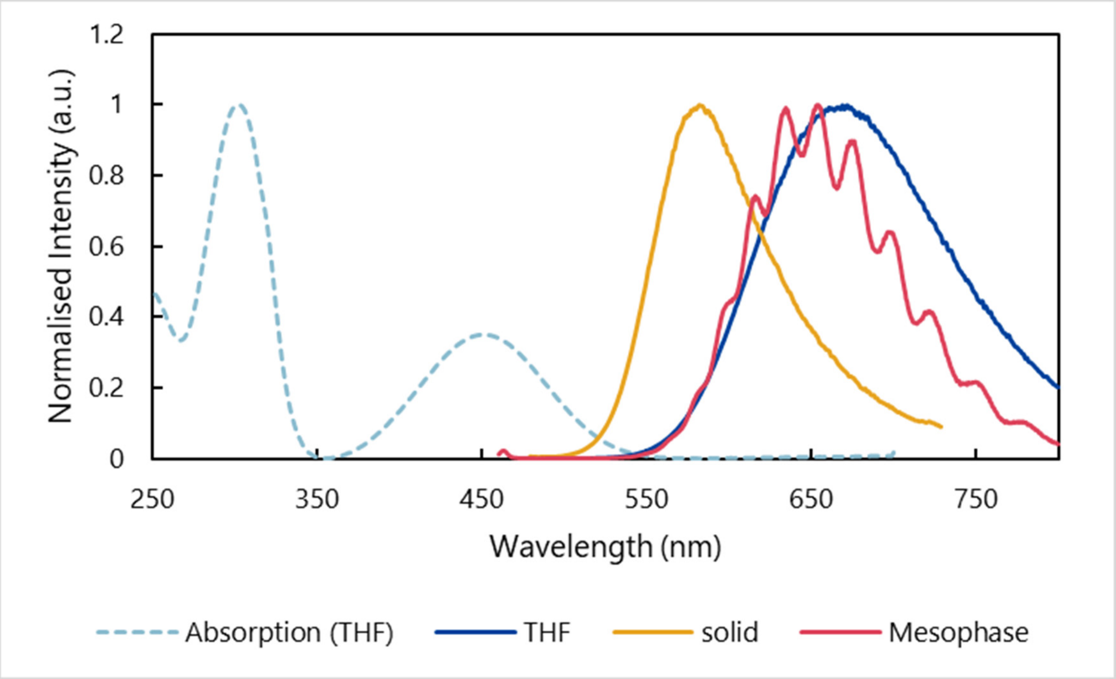

Figure S27. Absorption and fluorescence spectra of **Me_2NC8** in THF solution, the solid state, and the mesophase.

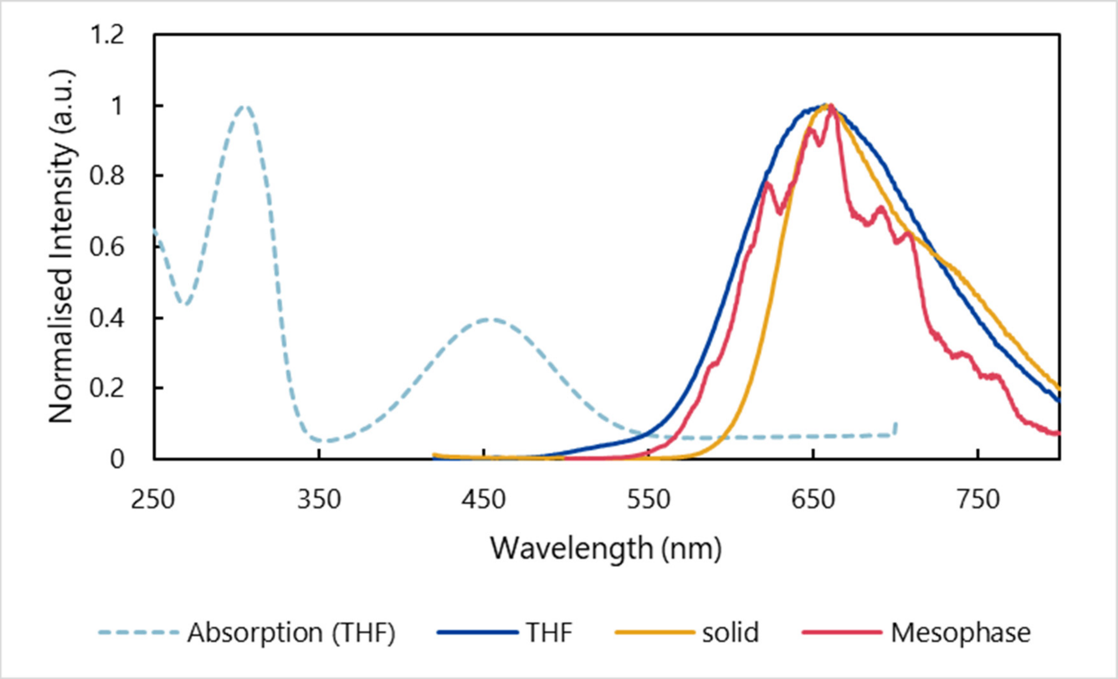

Figure S28. Absorption and fluorescence spectra of **Me_2NOC8** in THF solution, the solid state, and the mesophase.

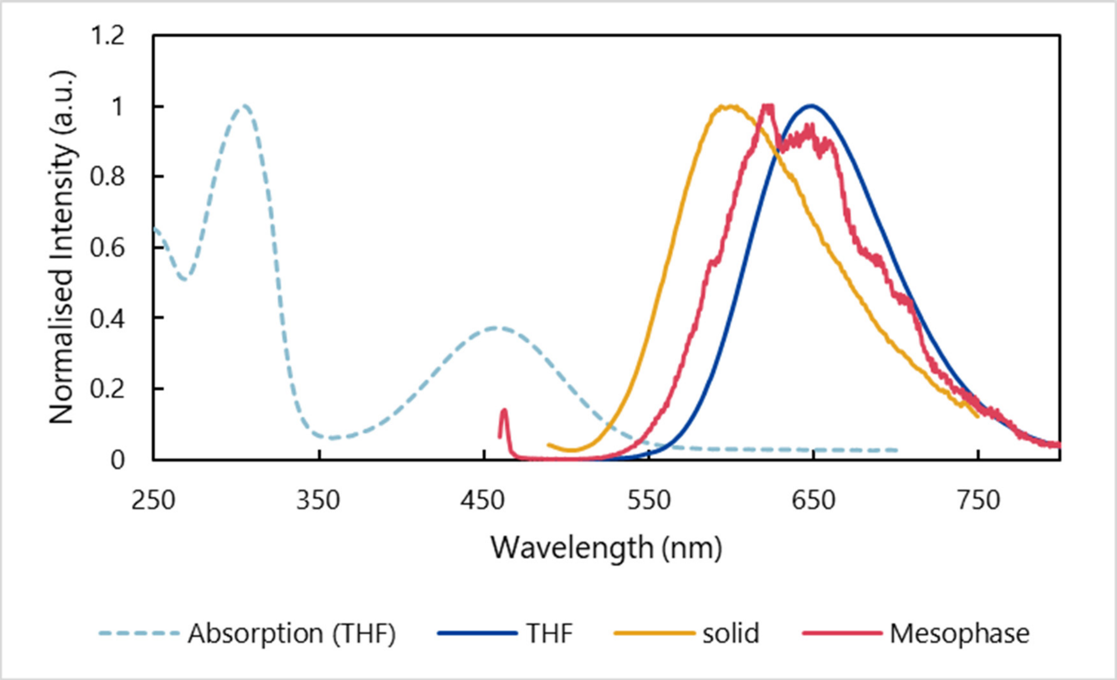

Figure S29. Absorption and fluorescence spectra of **EHNOC6** in THF solution, the solid state, and the mesophase.

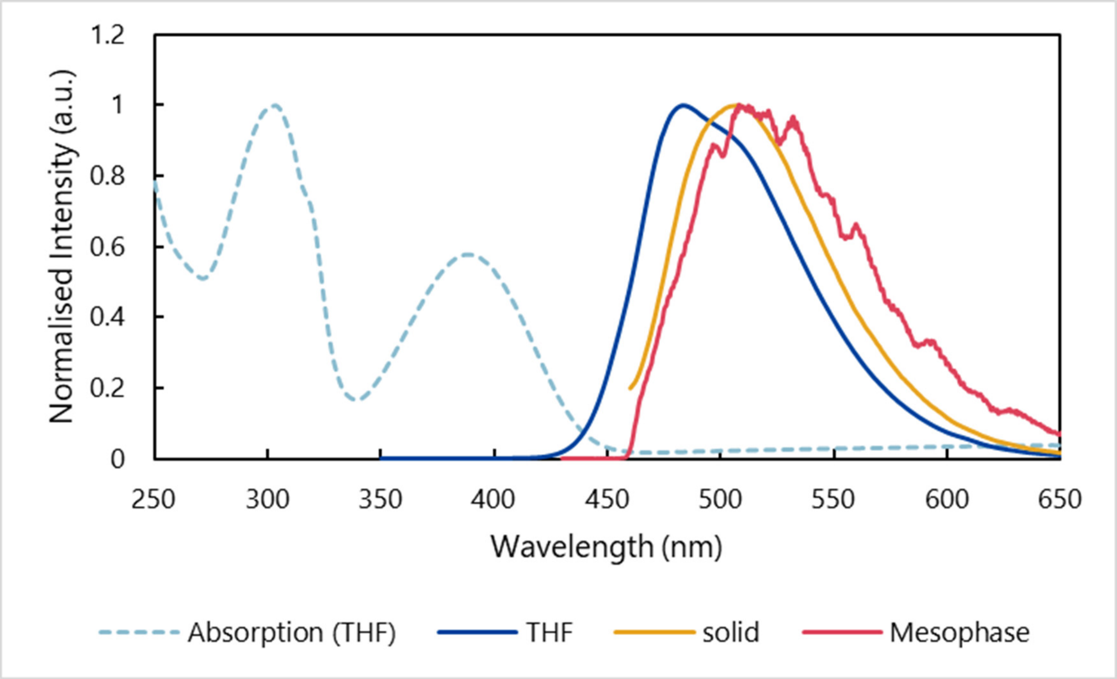

Figure S30. Absorption and fluorescence spectra of **AldC8** in THF solution, the solid state, and the mesophase.

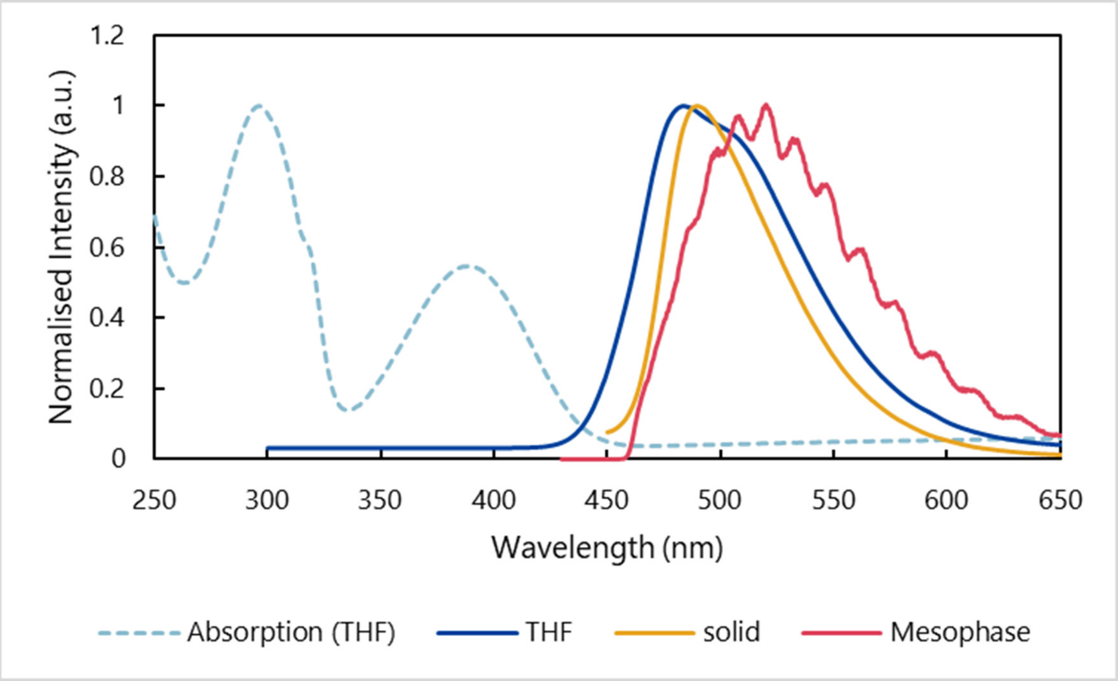

Figure S31. Absorption and fluorescence spectra of **ActC8** in THF solution, the solid state, and the mesophase.

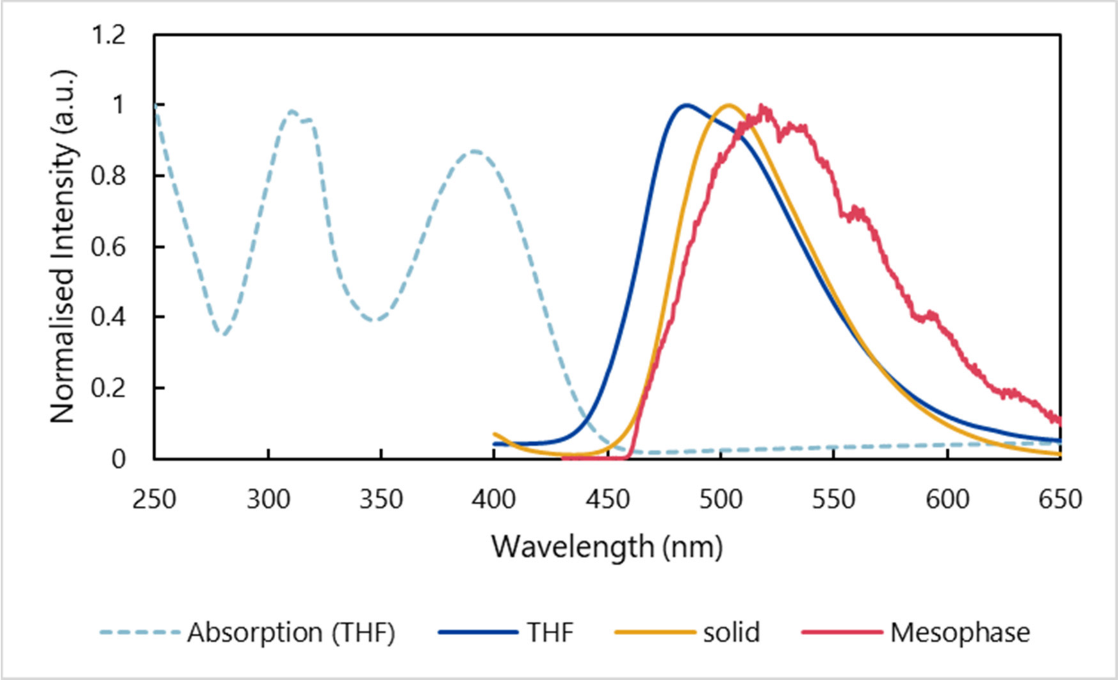

Figure S32. Absorption and fluorescence spectra of **TFActC8** in THF solution, the solid state, and the mesophase.

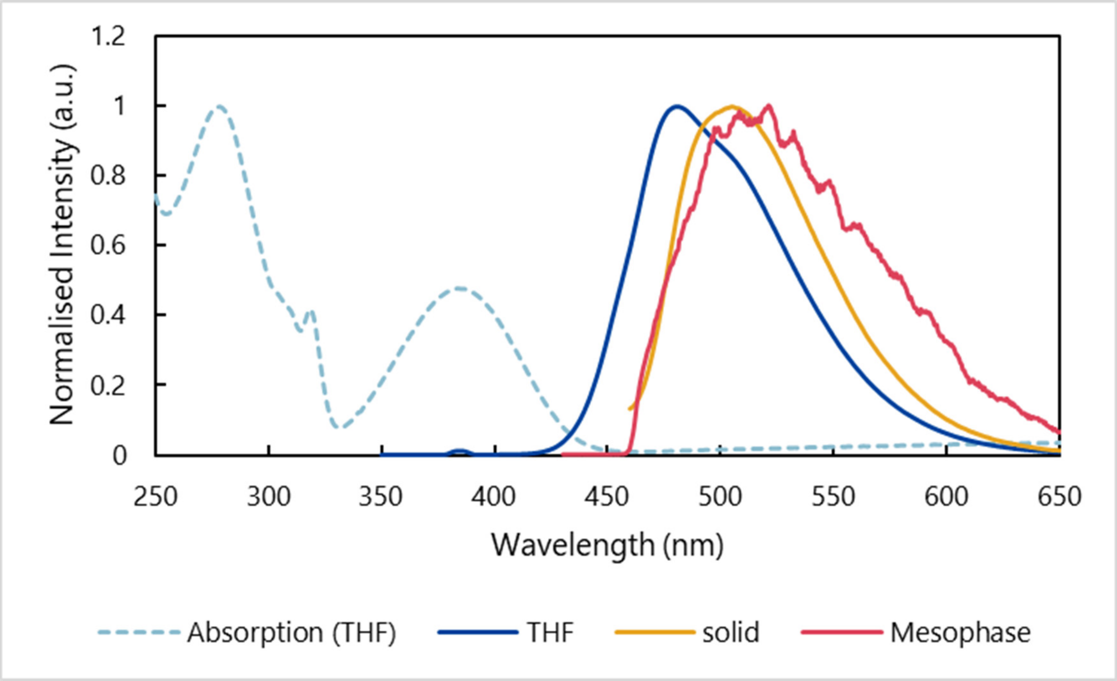

Figure S33. Absorption and fluorescence spectra of **TFMeC8** in THF solution, the solid state, and the mesophase.

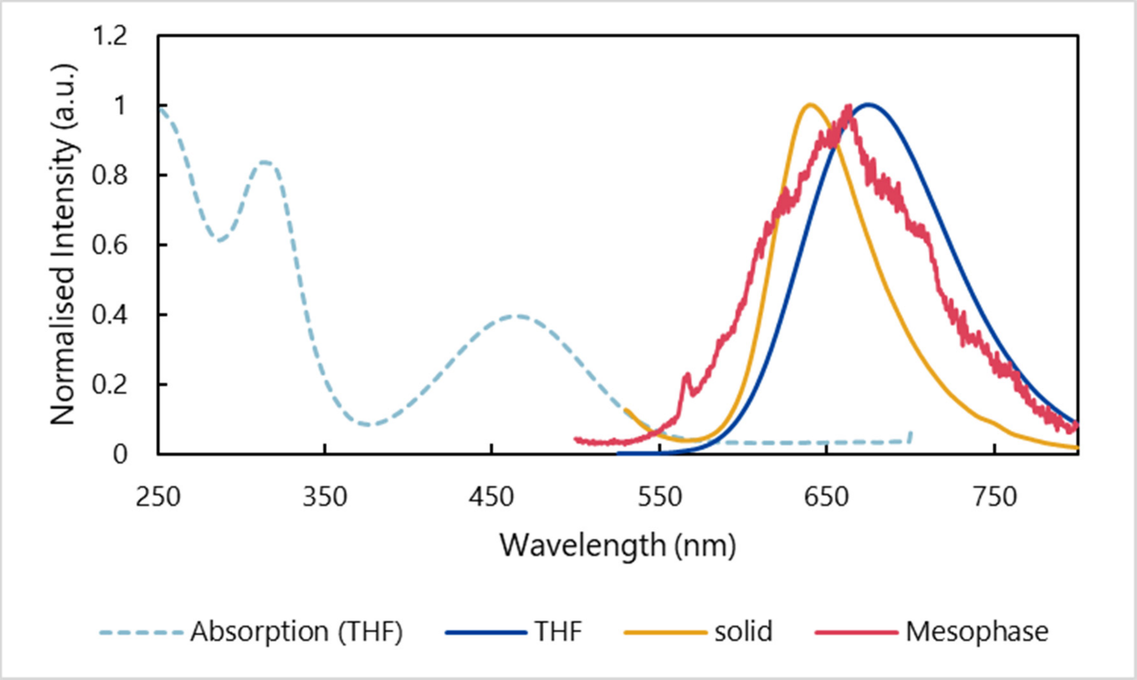

Figure S34. Absorption and fluorescence spectra of **C6NCN** in THF solution, the solid state, and the mesophase.

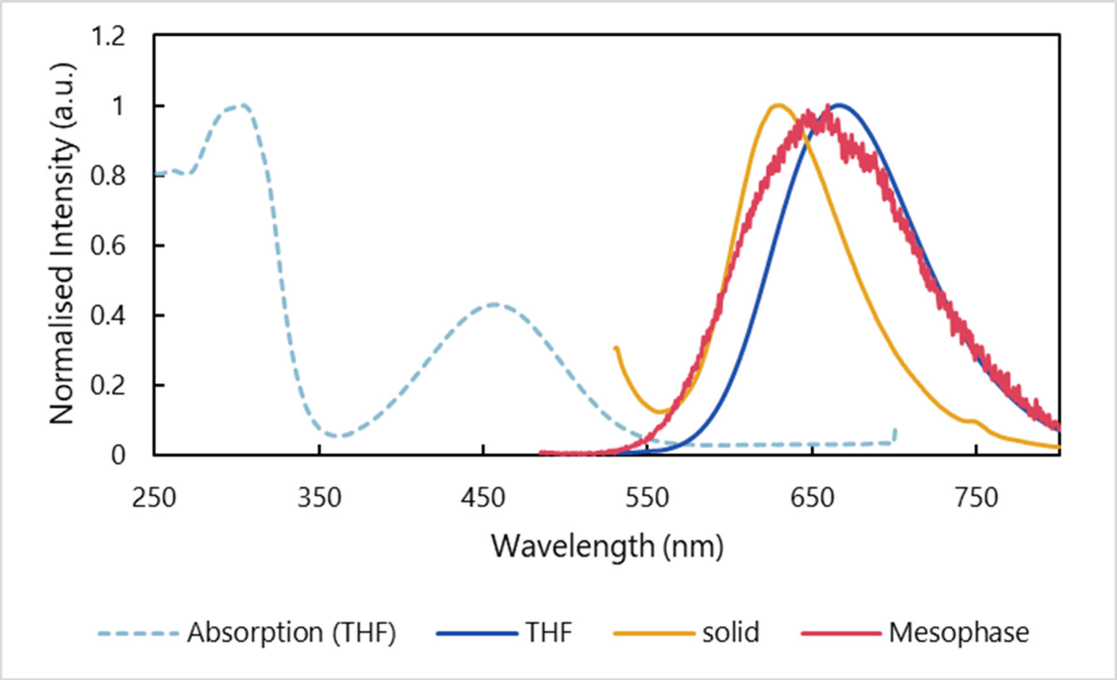

Figure S35. Absorption and fluorescence spectra of **C6NTFMe** in THF solution, the solid state, and the mesophase.

Experimental section

General procedure for Williamson ether synthesis

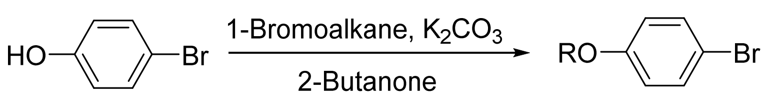

1a-d

a: R = C_6H_{13}
b: R = C_7H_{15}
c: R = C_8H_{17}
d: R = Ethylhexyl

Scheme S1. Wiliamson ether synthesis.

4-Bromophenol (1.0 eq), 1-bromoalkane (1.5 eq) and K_2CO_3 (2.5 eq) were dissolved in 2-butanone and refluxed at 80 °C overnight. After completion, the reaction was quenched with water, and the organic phase was extracted with ethyl acetate. The combined organic layer~~s~~ were dried over $MgSO_4$, filtered and evaporated under reduced pressure. The residue was purified by silica gel column chromatography using hexane as the eluent, affording the target compound.

1-bromo-4-(hexyloxy)benzene (1a)

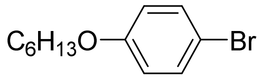

Synthesized from 4-bromophenol (1.04 g, 6.0 mmol), 1-bromohexane (1.24 ml, 9.0 mmol), K_2CO_3 (2.08 g, 15 mmol), and 2-butanone (15 ml). Colorless oil. Yield: 1.41 g (5.46 mmol), 91%; ^{1}H-NMR (500 MHz, $CDCl_3$) δ 7.36 (td, J = 2.8, 10.2 Hz, 2H, Ar**H**), 6.77 (td, J = 2.8, 10.2 Hz, 2H, Ar**H**), 3.91 (t, J = 6.6 Hz, 2H, OC**H**$_2$), 1.76 (m, 2H, C**H**$_2$), 1.47-1.41 (m, 2H, C**H**$_2$), 1.38-1.29 (m, 4H, C**H**$_2$), 0.91 (t, J = 7.0 Hz, 3H, C**H**$_3$) ppm (**Figure S36**).

1-bromo-4-(heptyloxy)benzene (1b)

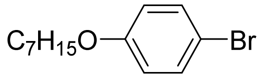

Synthesized from 4-bromophenol (1.04 g, 6.0 mmol), 1-bromoheptane (1.41 ml, 9.0 mmol), K_2CO_3 (2.07 g, 15 mmol), and 2-butanone (15 ml). Colorless oil. Yield: 1.65 g (5.9 mmol), 99%; ^{1}H-NMR (500 MHz, $CDCl_3$)δ 7.35 (td, J = 2.8, 10.2 Hz, 2H, Ar**H**), 6.77 (td, J = 2.8, 10.2 Hz, 2H, Ar**H**), 3.91 (t, J = 6.6 Hz, 2H, OC**H**$_2$), 1.79-1.74 (m, 2H, C**H**$_2$), 1.47-1.41 (m, 2H, C**H**$_2$), 1.38-1.26 (m, 6H, C**H**$_2$), 0.89 (t, J = 7.0 Hz, 3H, C**H**$_3$) ppm (**Figure S37).**

1-bromo-4-(octyloxy)benzene (1c)

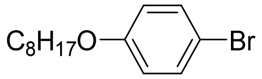

Synthesized from 4-bromophenol (1.04 g, 6.0 mmol), 1-bromooctane (1.57 ml, 9.0 mmol), K_2CO_3 (2.07 g, 15 mmol), and 2-butanone (15 ml). Colorless oil. Yield: 1.07 g (3.75 mmol), 63%; ^{1}H-NMR (500 MHz, $CDCl_3$) δ 7.36 (td, J = 2.8, 10.2 Hz, 2H, Ar**H**), 6.77 (td, J = 2.8, 10.2 Hz, 2H, Ar**H**), 3.91 (t, J = 6.6 Hz, 2H, OC**H**$_2$), 1.76 (m, 2H, C**H**$_2$), 1.46-1.41 (m, 2H, C**H**$_2$), 1.35-1.23 (m, 8H, C**H**$_2$), 0.89 (t, J = 7.0 Hz, 3H, C**H**$_3$) ppm (**Figure S38**).

1-bromo-4-((2-ethylhexyl)loxy)benzene (1d)

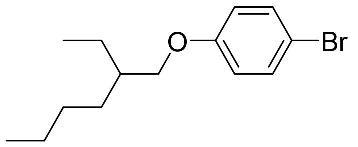

Synthesized from 4-bromophenol (1.04 g, 6.0 mmol), 1-bromo-2-ethylhexane (1.55 ml, 9.0 mmol), K_2CO_3 (2.07 g, 15 mmol), and 2-butanone (15 ml). Colorless oil. Yield: 1.0 g (3.51 mmol), 58% (some amount of 1-bromo-2-ethylhexane was contaminated); ^{1}H-NMR (500 MHz, $CDCl_3$) δ 7.35 (td, *J* = 2.8, 10.2 Hz, 2H, Ar**H**), 6.78 (td, *J* = 2.8, 10.2 Hz, 2H, Ar**H**), 3.82-3.77 (m, 2H, OC**H**$_2$), 1.73-1.68 (m, 1H, C**H**), 1.50-1.25 (m, 8H, C**H**$_2$), 0.92-0.89 (m, 6H, C**H**$_3$) ppm, (**Figure S39**).

Synthesis of 4-bromo-*N*-methyl-*N*-octylaniline (2)

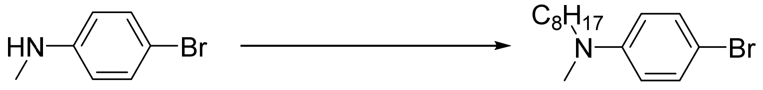

2

Scheme S2. Synthesis of **4-bromo-*N*-methyl-*N*-octylaniline**.

4-bromo-*N*-methylaniline (0.59 ml, 4.68 mmol), 1-iodooctane (1.35 ml, 7.5 mmol), K_2CO_3 (1.38 g, 10 mmol) were dissolved in argon purged dry acetone under an argon atmosphere. The mixture was stirred at room temperature overnight. Subsequently, the reaction was quenched with water, and the organic phase was extracted with ethyl acetate. The combined organic layers were dried over $MgSO_4$, filtered and evaporated under reduced pressure. The residue was purified by column chromatography on silica gel, eluting with a hexane/dichloromethane (5:1, v/v) mixture, yielding pure compound **2** as a brown oil. Yield: 0.37 g (1.24 mmol), 27%; ^{1}H-NMR (500 MHz, $CDCl_3$) δ 7.27 (td, *J* = 2.8, 10.4 Hz, 2H, Ar**H**), 6.54 (td, *J* = 2.8, 10.4 Hz, 2H, Ar**H**), 3.26 (t, *J* = 7.5 Hz, 2H, NC**H**$_2$), 2.89 (s, 3H, NC**H**$_3$), 1.57-1.51 (m, 2H, C**H**$_2$), 1.33-1.27 (m, 10H, C**H**$_2$), 0.88 (t, *J* = 6.9 Hz, 3H, C**H**$_3$) ppm (**Figure S40**).

General procedure for borylation

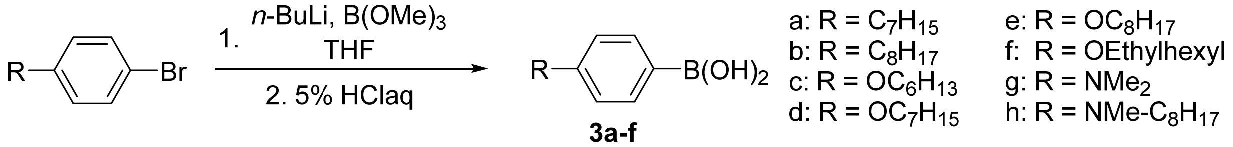

3a-f

Scheme S2. Borylation of aryl bromide.

Aryl bromide (1.0 eq) was dissolved in dry THF under an argon atmosphere, and the mixture was cooled to -78 °C. A 2.3 M solution of *n*-BuLi in cyclohexane (1.2 eq) was added dropwise via syringe, and the mixture was stirred at -78 °C for 1 hour. Trimethyl borate (3.0 eq) was then added dropwise via syringe. The mixture was stirred at -78 °C for 30 minutes, then allowed to warm to room temperature and stirred for an additional 3 hours. Subsequently, the reaction was quenched with 5% HCl *aq*. The organic phase was extracted with ethyl acetate. The combined organic layers were dried over $MgSO_4$, filtered and evaporated under reduced pressure to afford the crude product. The crude product was used as is for the next synthesis without purification, and its structure was confirmed by ^{1}H NMR spectroscopy.

(4-heptylphenyl)boronic acid (3a)

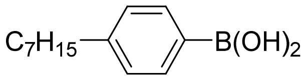

Synthesized from 1-bromo-4-*n*-heptylbenzene (0.44 ml, 2.0 mmol), 2.3 M *n*-BuLi (1.2 ml, 2.4 mmol), trimethyl borate (0.68 ml, 6.0 mmol), and dry THF (10 ml). Yellowish oil. Yield: 0.44 g (2.0 mmol).

(4-octylphenyl)boronic acid (3b)

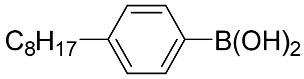

Synthesized from 1-bromo-4-*n*-octylbenzene (0.95 ml, 4.0 mmol), 2.3 M *n*-BuLi (2.4 ml, 4.8 mmol), trimethyl borate (1.4 ml, 12 mmol), and dry THF (10 ml). White solid. Yield: 0.94 g (4.0 mmol).

(4-(hexyloxy)phenyl)boronic acid (3c)

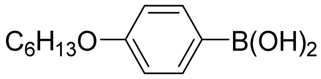

Synthesized from **1a** (1.41 g, 5.4 mmol), 2.3 M *n*-BuLi (2.9 ml, 6.6 mmol), trimethyl borate (1.8 ml, 16 mmol), and dry THF (10 ml). White solid. Yield: 1.27 g (5.7 mmol).

(4-(heptyloxy)phenyl)boronic acid (3d)

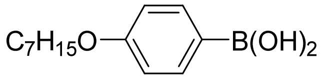

Synthesized from **1b** (1.08 g, 4.0 mmol), 2.3 M *n*-BuLi (2.4 ml, 4.8 mmol), trimethyl borate (1.36 ml, 12 mmol), and dry THF (10 ml). White solid. Yield: 0.90 g (4.07 mmol).

(4-(octyloxy)phenyl)boronic acid (3e)

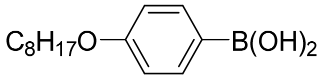

Synthesized from **1c** (1.07 g, 3.75 mmol), 2.3 M *n*-BuLi (1.97 ml, 4.5 mmol), trimethyl borate (1.3 ml, 11 mmol), and dry THF (10 ml). White solid. Yield: 1.02 g (4.1 mmol).

(4-((2-ethylhexyl)oxy)phenyl)boronic acid (3f)

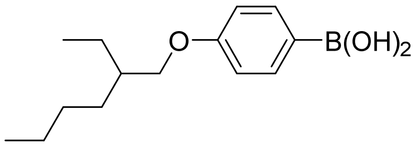

Synthesized from **1d** (1,0 g, 3.5 mmol), 2.3 M *n*-BuLi (1.8 ml, 4.2 mmol), trimethyl borate (1.2 ml, 11 mmol), and dry THF (10 ml). Yellow oil. Yield: 1.08 g (4.32 mmol).

(4-(dimethylamino)phenyl)boronic acid (3g)

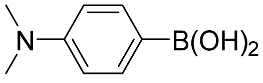

Synthesized from 4-bromo-*N,N*-dimethylaniline (1.2 g, 6.0 mmol), 2.3 M *n*-BuLi (3.2 ml, 7.3 mmol), trimethyl borate (1.4 ml, 18 mmol), and dry THF (10 ml. White solid. Yield: 0.39 g (2.4 mmol).

(4-(methyl(octyl)amino)phenyl)boronic acid (3h)

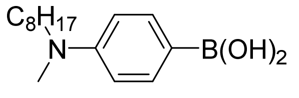

Synthesized from **2** (0.37 g, 1.2 mmol), 2.3 M *n*-BuLi (0.75 ml, 1.5 mmol), trimethyl borate (0.42 ml, 3.7 mmol), and dry THF (10 ml). Yellow oil. Yield: 0.22 g (0.84 mmol).

General procedure for alkylation of amines

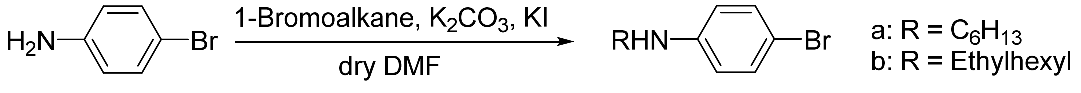

4a,b

Scheme S3. Alkylation of amines.

4-Bromoaniline (1.0 eq), 1-bromoalkane (1.0 eq), K_2CO_3 (1.8 eq) and KI (0.3 eq) were dissolved in argon purged dry DMF. The mixture was refluxed at 80 °C overnight. After completion, the reaction was quenched with water, and the organic phase was extracted with ethyl acetate. The combined organic layers were dried over $MgSO_4$, filtered and evaporated under pressure. The residue was purified by chromatography on silica gel, eluting with a hexane/dichloromethane (6:1, v/v) mixture to afford the target compound.

4-bromo-N-hexylaniline (4a)

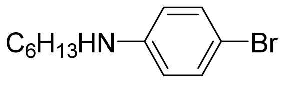

Synthesized from 4-bromoaniline (1.72 g, 10 mmol), 1-bromohexane (1.4 ml, 10 mmol), K_2CO_3 (2.5 g, 18 mmol), KI (0.49 g, 3.0 mmol), and dry DMF (8 ml). Yellow oil. Yield: 1.51 g (5.9 mmol), 59%; ^{1}H-NMR (500 MHz, $CDCl_3$) δ 7.23 (td, *J* = 2.7, 9.9 Hz, 2H, Ar**H**), 6.46 (td, *J* = 2.7, 9.9 Hz, 2H, Ar**H**), 3.62 (s, 1H, N**H**), 3.06 (t, *J* = 7.2 Hz, 2H, NC**H**2), 1.63-1.57 (m, 2H, C**H**2), 1.41-1.30 (m, 6H, C**H**2), 0.90 (t, *J* = 7.0 Hz, 3H, C**H**3) ppm (**Figure S41**).

4-bromo-N-(2-ethylhexyl)aniline (4b)

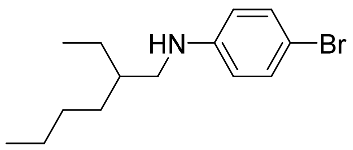

Synthesized from 4-bromoaniline (1.72 g, 10 mmol), 1-bromo-2-ethylhexane (1.72 ml, 10 mmol), K_2CO_3 (2.48 g, 18 mmol), KI (0.49 g, 3.0 mmol), and dry DMF (10 ml). Yield:2.17 g (7.3 mmol), 73%; ^{1}H-NMR (500 MHz, $CDCl_3$) δ 7.23 (td, *J* = 2.7, 10.0 Hz, 2H, Ar**H**), 6.47 (td, *J* = 2.7, 10.0 Hz, 2H, Ar**H**), 3.66 (s, 1H, N**H**), 2.98 (d, *J* = 6.1 Hz, 2H, NC**H**2), 1.58-1.51 (m, 1H, C**H**), 1.44-1.25 (m, 7H, C**H**2), 0.92-0.89 (m, 6H, C**H**3) ppm (**Figure S42**).

General procedure for boronic acid pinacol ester

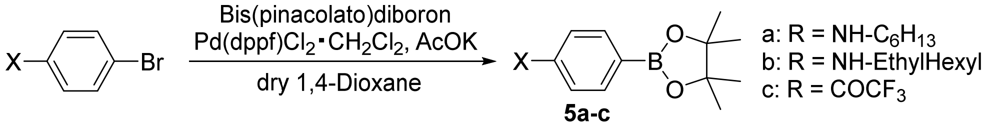

Scheme S4. Miyaura-Ishiyama borylation.

Aryl bromide (1.0 eq), bis(pinacolato)diboron (1.5 eq), Pd(dppf)Cl_2·CH_2Cl_2 (3.0 mol%), and potassium acetate (3.0 eq) were dissolved in argon purged 1,4-dioxane. The mixture was heated at 100 °C for more than 24 hours under an argon atmosphere. The reaction was then quenched with water, and the organic phase was extracted with ethyl acetate. The combined organic layers were dried over $MgSO_4$, filtrated, and evaporated under reduced pressure. The residue was purified by column chromatography on silica gel to afford the target compound.

***N*-hexyl-4-(4,4,5,5-tetramethyl-1,3,2-dioxaborolan-2-yl)aniline (5a)**

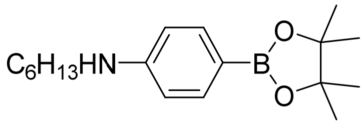

Synthesized from **4a** (1.51 g, 5.9 mmol), bis(pinacolato)diboron (2.3 g, 8.9 mmol), Pd(dppf)Cl_2 CH_2Cl_2 (0.14 g, 0.18 mmol), AcOK (1.7 g, 18 mmol), and dry 1,4-dioxane (10 ml). Eluting with a hexane/ethyl acetate (20:1, v/v) mixture on silica gel column chromatography. Yellow oil. Yield: 1.73 g (5.8 mmol), 98% (some amount of homocoupling derivative and bis(pinacolato)diboron were contaminated); ^{1}H-NMR (500 MHz, $CDCl_3$) δ 7.62 (td, *J* = 1.9, 8.7 Hz, 2H, Ar**H**), 6.56 (td, *J* = 1.9, 8.6 Hz, 2H, Ar**H**), 3.80 (s, 1H, N**H**), 3.13 (t, *J* = 7.2 Hz, 2H, NC**H**$_2$), 1.64-1.57 (m, 2H, C**H**$_2$), 1.35-1.29 (m, 16H, C**H**$_2$, C**H**$_3$), 0.91-0.88 (m, 3H, C**H**$_3$) ppm (**Figure S43**).

***N*-(2-ethylhexyl)-4-(4,4,5,5-tetramethyl-1,3,2-dioxaborolan-2-yl)aniline (5b)**

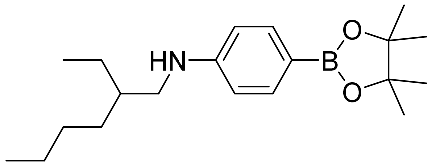

Synthesized from **4b** (2.2 g, 7.3 mmol), bis(pinacolato)diboron (2.7 g, 11 mmol), Pd(dppf)Cl_2 CH_2Cl_2 (0.18 g, 0.22 mmol), AcOK (3.6 g, 37 mmol), and dry 1,4-dioxane (15 ml). Eluting a hexane/ethyl acetate (15:1, v/v) mixture on silica gel column chromatography. Yellow oil. Yield: 1.7 g, 70%; ^{1}H-NMR (500 MHz, $CDCl_3$) δ 7.62 (dt, *J* = 8.6, 1.9 Hz, 2H, Ar**H**), 6.56 (dt, *J* = 8.6, 1.9 Hz, 2H, Ar**H**), 3.83 (s, 1H, N**H**), 3.04 (d, J = 6.1 Hz, 2H, C**H**$_2$), 1.58-1.52 (m, 3H, C**H**, C**H**$_2$), 1.44-1.33 (m, 6H, C**H**$_2$), 1.31 (s, 12H, C**H**$_3$), 0.92-0.88 (m, 6H, C**H**$_3$) ^{1}H-NMR (500 MHz, $CDCl_3$) δ 7.62 (td, *J* = 1.9, 8.7 Hz, 2H, Ar**H**), 6.57 (td, *J* = 1.9, 8.8 Hz, 2H, Ar**H**), 3.83 (s, 1H, N**H**), 3.08-3.01 (m, 2H, NC**H**$_2$), 1.58-1.53 (m, 1H, C**H**), 1.44-1.26 (m, 20H, C**H**$_2$, C**H**$_3$), 0.94-0.87 (m, 6H, C**H**$_3$) ppm (**Figure S44**).

2,2,2-trifluoro-1-(4-(3,3,4,4-tetramethylborolan-1-yl)phenyl)ethan-1-one (5c)

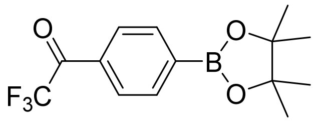

Synthesized from 4'-bromo-2,2,2-trifluoroacetophenone (0.51 g, 2.0 mmol), bis(pinacolato)diboron (1.05 g, 4.0 mmol), Pd(dppf)Cl_2 CH_2Cl_2 (0.049 g, 0.06 mmol), AcOK (0.59 g, 6.0 mmol), and dry 1,4-

dioxane (15 ml). Eluting with 4/1 (v/v) hexane/ethyl acetate on column chromatography. Brown oil. Yield: 0.70 g (2.3 mmol), 99% (some amount of bis(pinacolato)diboron was contaminated); ^{1}H-NMR (500 MHz, $CDCl_3$) δ 8.04 (d, J = 7.3 Hz, 2H, Ar**H**), 7.96 (d, J = 8.2 Hz, 2H, Ar**H**), 1.36 (s, 12H, C**H**$_3$) ppm (**Figure S45**).

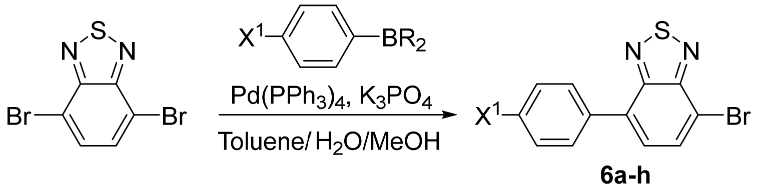

a: X^1 = NMe_2
b: X^1 = C_7H_{15}
c: X^1 = NH-C_6H_{13}
d: X^1 = NH-Ethylhexyl
e: X^1 = CN
f: X^1 = CHO
g: X^1 = $COCH_3$
h: X^1 = $COCF_3$
i: X^1 = CF_3

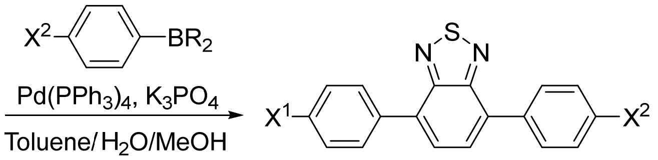

Me₂NC7 (X^1 = NMe_2, X^2 = C_7H_{15})
Me₂NC8 (X^1 = NMe_2, X^2 = C_8H_{17})
Me₂NOC7 (X^1 = NMe_2, X^2 = OC_7H_{15})
Me₂NOC8 (X^1 = NMe_2, X^2 = OC_8H_{17})
Me₂NOEH (X^1 = NMe_2, X^2 = OEthylhexyl)
C8MeNC7 (X^1 = C_7H_{15}, X^2 = NMe-C_8H_{17})
C6NOC6 (X^1 = NH-C_6H_{13}, X^2 = OC_6H_{13}
EHNOC6 (X^1 = NH-Ethylhexyl, X^2 = OC_6H_{13})
EHNOEH (X^1 = NH-Ethylhexyl, X^2 = OEthylhexyl)
C6NCN (X^1 = CN, X^2 = NH-C_6H_{13})
AldC8 (X^1 = CHO, X^2 = C_8H_{17})
ActC8 (X^1 = $COCH_3$, X^2 = C_8H_{17})
TFActC8 (X^1 = $COCF_3$, X^2 = C_8H_{17})
TFMeC8 (X^1 = CF_3, X^2 = C_8H_{17})

Scheme S5. Suzuki-Miyaura coupling

General procedure for Suzuki-Miyaura cross-coupling

4,7-dibromobenzo[c][1,2,5]thiadiazole or aryl bromide (2.0 eq/1.0 eq), aryl boronic acid or aryl boronic acid pinacol ester (1.0–2.0 eq), K_3PO_4 (3.0 eq) and $Pd(PPh_3)_4$ (3.0 mol%) were dissolved in a 5:2:1 (v/v) mixture of toluene/water/methanol under an argon atmosphere. The mixture was refluxed at 100 °C overnight. The reaction was then quenched with water, and the organic phase was extracted with ethyl acetate. The combined organic layers were dried over $MgSO_4$, filtered and evaporated under reduced pressure. The residue was purified by column chromatography on silica gel to afford the target compound.

4-(7-bromobenzo[c][1,2,5]thiadiazol-4-yl)-*N*,*N*-dimethylaniline (6a)

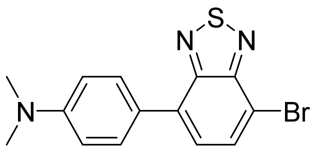

Synthesized from 4,7-dibromobenzo[c][1,2,5]thiadiazole (1.18 g, 4.0 mmol), **3g** (0.38 g, 2.3 mmol), K_3PO_4 (1.27 g, 6.0 mmol), $Pd(PPh_3)_4$, and a 5:2:1 (v/v) mixture of toluene/water/methanol (10 ml). Eluting with a hexane/dichloromethane (3:1, v/v) mixture on silica gel column chromatography. Orange solid. Yield: 0.42 g (1.26 mmol), 55%; ^{1}H-NMR (500 MHz, $CDCl_3$) δ 7.88-7.84 (m, 3H, Ar**H**), 7.51 (d, J = 7.6 Hz, 1H, Ar**H**), 6.86 (td, J = 2.5, 9.9 Hz, 2H, Ar**H**), 3.05 (s, 6H, NC**H**$_3$) ppm (**Figure S46**).

4-bromo-7-(4-heptylphenyl)benzo[c][1,2,5]thiadiazole (6b)

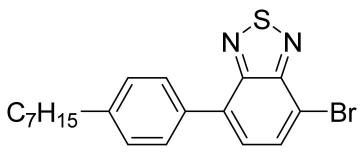

Synthesized from 4,7-dibromobenzo[c][1,2,5]thiadiazole (0.59 g, 2.0 mmol), **3a** (0.22 g, 1.0 mmol), K_3PO_4 (0.64 g, 3.0 mmol), $Pd(PPh_3)_4$, and a 5:2:1 (v/v) mixture of toluene/water/methanol (10 ml). Eluting with a hexane/dichloromethane (2:1, v/v) mixture on silica gel column chromatography. Green solid. Yield: 0.31 g (0.80 mmol), 80%; ^{1}H-NMR (500 MHz, $CDCl_3$) δ 7.91 (d, J = 7.6 Hz, 1H, Ar**H**), 7.82-7.80 (m, 2H, Ar**H**), 7.56 (d, J = 7.6 Hz, 1H, Ar**H**), 7.35 (d, J = 8.2 Hz, 2H, C**H**$_2$), 2.69 (t, J = 7.8 Hz, 2H, C**H**$_2$), 1.71-1.65 (m, 2H, C**H**$_2$), 1.41-1.24 (m, 8H, C**H**$_2$), 0.90-0.88 (t, J = 7.0 Hz, 3H, C**H**$_3$) ppm (**Figure S47**).

4-(7-bromobenzo[c][1,2,5]thiadiazol-4-yl)-*N*-hexylaniline (6c)

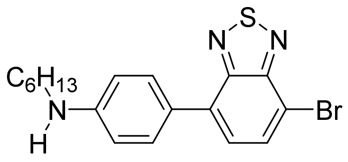

Synthesized from 4,7-dibromobenzo[c][1,2,5]thiadiazole (1.18 g, 4.0 mmol), **5a** (0.60 g, 2.0 mmol), K_3PO_4 (1.27 g, 6.0 mmol), $Pd(PPh_3)_4$, and a 5:2:1 (v/v) mixture of toluene/water/methanol (10 ml). Eluting with a hexane/dichloromethane (1:1, v/v) mixture on silica gel column chromatography. Red oil. Yield: 0.34 g (0.87 mmol), 44% (some amount of homocoupling byproduct was contaminated); ^{1}H-NMR (500 MHz, $CDCl_3$) δ 7.86 (d, J = 7.9 Hz, 1H, Ar**H**), 7.79 (td, J = 2.4, 9.5 Hz, 2H, Ar**H**), 7.49 (d, J = 7.6 Hz, 1H, Ar**H**), 6.73 (td, J = 2.4, 9.3 Hz, 2H, Ar**H**), 3.88 (s, 1H, N**H**), 3.19 (t, J = 7.0 Hz, 2H, NC**H**$_2$), 1.69-1.63 (m, 2H, C**H**$_2$), 1.45-1.40 (m, 2H, C**H**$_2$), 1.35-1.31 (m, 4H, C**H**$_2$), 0.93-0.90 (m, 3H, C**H**$_3$) ppm (**Figure S48**).

4-(7-bromobenzo[c][1,2,5]thiadiazol-4-yl)-N-(2-ethylhexyl)aniline (6d)

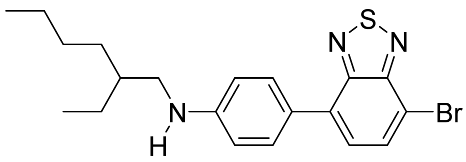

Synthesized from 4,7-dibromobenzo[c][1,2,5]thiadiazole (1.4 g, 4.8 mmol), **5b** (0.81 g, 2.4 mmol), K_3PO_4 (1.55 g, 7.3 mmol), $Pd(PPh_3)_4$ (0.085 g, 0.070 mmol), and a 5:2:1 (v/v) mixture of toluene/water/methanol (10 ml). Eluting with a hexane/dichloromethane (2:1, v/v) mixture on silica gel column chromatography. Yellow solid. Yield: 0.16 g (0.38 mmol), 16%; ^{1}H-NMR (500 MHz, $CDCl_3$) δ 7.86 (d, J = 7.6 Hz, 1H, Ar**H**), 7.78 (td, J = 2.5, 9.7 Hz, 2H, Ar**H**), 7.49 (d, J = 7.6 Hz, 1H, Ar**H**), 6.73 (td, J = 2.4, 9.5 Hz, 2H, Ar**H**), 3.90 (s, 1H, N**H**), 3.14-3.07 (m, 2H, NC**H**$_2$), 1.65-1.58 (m, 1H, C**H**), 1.48-1.30 (m, 8H, C**H**$_2$), 0.95-0.90 (m, 6H, C**H**$_3$) ppm (**Figure S49**).

4-(7-bromobenzo[c][1,2,5]thiadiazol-4-yl)benzonitrile (6e)

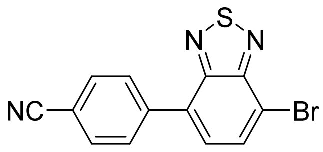

Synthesized from 4,7-dibromobenzo[c][1,2,5]thiadiazole (0.88 g, 3.0 mmol), 4-cyanophenylboronic acid (0.29 g, 2.0 mmol), K_3PO_4 (1.27 g, 6.0 mmol), $Pd(PPh_3)_4$ (0.069 g, 0.060 mmol), and a 5:2:1 (v/v) mixture of toluene/water/methanol (10 ml). Eluting with a hexane/dichloromethane (1:1, v/v) mixture on silica gel column chromatography. Green solid. Yield: 0.17 g (0.54 mmol), 26%; ^{1}H-NMR (500 MHz, $CDCl_3$) δ 8.04 (td, *J* = 1.8, 8.4 Hz, 2H, Ar**H**), 7.97 (d, *J* = 7.6 Hz, 1H, Ar**H**), 7.82 (td, *J* = 1.8, 8.3 Hz, 2H, Ar**H**), 7.63 (d, *J* = 7.6 Hz, 1H, Ar**H**) ppm (**Figure S50**).

4-(7-bromobenzo[c][1,2,5]thiadiazol-4-yl)benzaldehyde (6f)

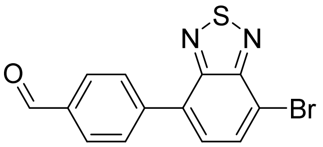

Synthesized from 4,7-dibromobenzo[c][1,2,5]thiadiazole (0.88 g, 3.0 mmol), 4-formylphenylboronic acid (0.23 g, 1.5 mmol), K_3PO_4 (0.96 g, 4.5 mmol), $Pd(PPh_3)_4$, and a 5:2:1 (v/v) mixture of toluene/water/methanol (10 ml). Eluting with a hexane/dichloromethane (1:1, v/v) mixture on silica gel column chromatography. Green solid. Yield: 0.18 g (0.6 mmol), 40%; ^{1}H-NMR (500 MHz, $CDCl_3$) δ 10.12 (s, 1H, C**HO**), 8.10-8.09 (m, 2H, Ar**H**), 8.06-8.04 (m, 2H, Ar**H**), 7.98 (d, *J* = 7.6 Hz, 1H, Ar**H**), 7.66 (d, *J* = 7.6 Hz, 1H, Ar**H**) ppm (**Figure S51**).

1-(4-(7-bromobenzo[c][1,2,5]thiadiazol-4-yl)phenyl)ethan-1-one (6g)

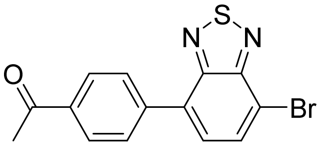

Synthesized from 4,7-dibromobenzo[c][1,2,5]thiadiazole (0.88 g, 3.0 mmol), 4-acetylphenylboronic acid (0.25 g, 1.5 mmol), K_3PO_4 (0.96 g, 4.5 mmol), $Pd(PPh_3)_4$, and a 5:2:1 (v/v) mixture of toluene/water/methanol (10 ml). Eluting with a hexane/dichloromethane (1:1, v/v) mixture on silica gel column chromatography. Green solid. Yield: 0.16 g (0.51 mmol), 33%; ^{1}H-NMR (500 MHz, $CDCl_3$) δ 8.12 (td, *J* = 1.9, 8.4 Hz, 2H, Ar**H**), 8.01 (td, *J* = 1.9, 8.4 Hz, 2H, Ar**H**), 7.97 (d, *J* = 7.3 Hz, 1H, Ar**H**), 7.64 (d, *J* = 7.6 Hz, 1H, Ar**H**), 2.68 (s, 3H, C**H**$_3$) ppm (**Figure S52**).

1-(4-(7-bromobenzo[c][1,2,5]thiadiazol-4-yl)phenyl)-2,2,2-trifluoroethan-1-one (6h)

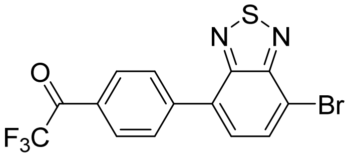

Synthesized from 4,7-dibromobenzo[c][1,2,5]thiadiazole (1.18 g, 4.0 mmol), **5c** (0.70 g, 2.3 mmol), K_3PO_4 (1.27 g, 6.0 mmol), $Pd(PPh_3)_4$, and a 5:2:1 (v/v) mixture of toluene/water/methanol (10 ml). Eluting with a hexane/dichloromethane (5:1, v/v) mixture on silica gel column chromatography. Green solid. Yield: 0.20 g (0.59 mmol), 25%; ^{1}H-NMR (500 MHz, $CDCl_3$) δ 8.24 (d, *J* = 8.5 Hz, 2H, Ar**H**), 8.12 (d, *J* = 8.2 Hz, 2H, Ar**H**), 7.99 (d, *J* = 7.6 Hz, 1H, Ar**H**), 7.68 (d, *J* = 7.3 Hz, 1H, Ar**H**) ppm (**Figure S53**).

4-bromo-7-(4-(trifluoromethyl)phenyl)benzo[c][1,2,5]thiadiazole (6i)

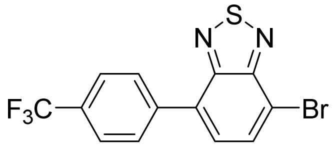

Synthesized from 4,7-dibromobenzo[c][1,2,5]thiadiazole (0.88 g, 3.0 mmol), 4-(trifluoromethyl)phenylboronic acid (0.28 g, 1.5 mmol), K_3PO_4 (0.96 g, 4.5 mmol), $Pd(PPh_3)_4$ (0.052 g, 0.045 mmol), and a 5:2:1 (v/v) mixture of toluene/water/methanol (10 ml). Eluting with a hexane/dichloromethane (1:1, v/v) mixture on silica gel column chromatography. Green solid. Yield: 0.37 g (1.03 mmol), 69% (some amount of di-cross-coupling byproduct was contaminated); ^{1}H-NMR (500 MHz, $CDCl_3$) δ 8.13 (d, J = 7.9 Hz, 1H, Ar**H**), 7.99 (d, J = 7.6 Hz, 1H, Ar**H**), 7.83 (dd, J = 14.2, 8.1 Hz, 3H, Ar**H**), 7.65 (d, J = 7.6 Hz, 1H, Ar**H**) ppm (**Figure S54**).

4-(7-(4-heptylphenyl)benzo[c][1,2,5]thiadiazol-4-yl)-*N*,*N*-dimethylaniline (Me_2NC7)

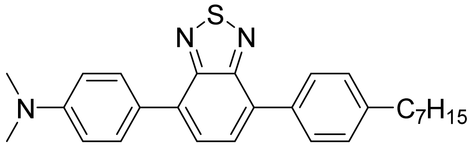

Synthesized from **6a** (0.11 g, 0.28 mmol), **3a** (0.14 g, 0.42 mmol), K_3PO_4 (0.18 g, 0.84 mmol), $Pd(PPh_3)_4$ (0.01 g, 0.01 mmol), and a 5:2:1 (v/v) mixture of toluene/water/methanol (10 ml). Eluting with a hexane/dichloromethane (3:2, v/v) mixture on silica gel column chromatography. Orange solid. Yield: 0.06 g (0.14 mmol), 50%; ^{1}H-NMR (500 MHz, $CDCl_3$) δ 7.93 (td, J = 2.6, 9.9 Hz, 2H, Ar**H**), 7.87 (td, J = 1.9, 8.4 Hz, 2H, Ar**H**), 7.74 (d, J = 7.3 Hz, 1H, Ar**H**), 7.71 (d, J = 7.3 Hz, 1H, Ar**H**), 7.35 (d, J = 8.2 Hz, 2H, Ar**H**), 6.90 (td, J = 2.4, 9.9 Hz, 2H, Ar**H**), 3.05 (s, 6H, NC**H**$_3$), 2.69 (t, J = 7.6 Hz, 2H, C**H**$_2$), 1.72-1.66 (m, 2H, C**H**$_2$), 1.42-1.25 (m, 8H, C**H**$_2$), 0.90 (t, J = 7.0 Hz, 3H, C**H**$_3$) ppm (**Figure S55**). ^{13}C-NMR (126 MHz, $CDCl_3$) δ 154.5, 150.7, 143.2, 135.2, 133.4, 131.9, 130.2, 129.1, 128.8, 128.2, 126.5, 125.5, 112.5, 40.6, 36.0, 32.0, 31.6, 29.5, 29.4, 22.8, 14.3 ppm (**Figure S56**). FT-IR (**Figure S85**); HRMS (EI) Calcd for $C_{27}H_{31}N_3S$: 429.2239, Found 429.2246 (**Figure S100**).

4-(7-(4-octylphenyl)benzo[c][1,2,5]thiadiazol-4-yl)-N,N-dimethylaniline (Me_2NC8)

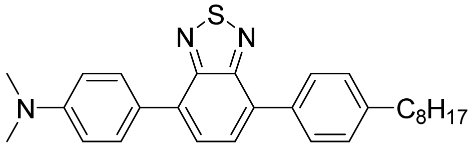

Synthesized from **6a** (0.10 g, 0.30 mmol), **3b** (0.11g, 0.45 mmol), K_3PO_4 (0.19 g, 0.90 mmol), $Pd(PPh_3)_4$, (1.5 mg, 9.0 µmol) and a 5:2:1 (v/v) mixture of toluene/water/methanol (10 ml). Eluting with a hexane/dichloromethane (3:2, v/v) mixture on silica gel column chromatography. Orange solid. Yield: 0.09 g (0,24 mmol), 68%; ^{1}H-NMR (500 MHz, $CDCl_3$) δ 7.93 (td, J = 2.5, 9.8 Hz, 2H, Ar**H**), 7.87 (td, J = 1.9, 8.3 Hz, 2H, Ar**H**), 7.74 (d, J = 7.3 Hz, 1H, Ar**H**), 7.71 (d, J = 7.3 Hz, 1H, Ar**H**), 7.35 (d, J = 8.2 Hz, 2H, Ar**H**), 6.90 (td, J = 2.5, 9.9 Hz, 2H, Ar**H**), 3.05 (s, 6H, NC**H**$_3$), 2.69 (t, J = 7.8 Hz, 2H, C**H**$_2$), 1.72-1.66 (m, 2H, C**H**$_2$), 1.42-1.24 (m, 10H, C**H**$_2$), 0.89 (t, J = 6.9 Hz, 3H, C**H**$_3$) ppm (**Figure S57**). ^{13}C-NMR (126 MHz, $CDCl_3$) δ 154.5, 150.7, 143.2, 135.2, 133.3, 131.9, 130.2, 129.1, 128.8, 128.2, 126.5, 125.5, 112.5, 40.6, 36.0, 32.1, 31.6, 29.7, 29.6, 29.4, 22.8, 14.3 ppm (**Figure S58**). FT-IR (**Figure S86**); HRMS (EI) Calcd for $C_{28}H_{33}N_3S$: 443.2395, Found 443.2393 (**Figure S101**).

4-(7-(4-(hexyloxy)phenyl)benzo[c][1,2,5]thiadiazol-4-yl)-*N*,*N*-dimethylaniline (Me_2NOC7)

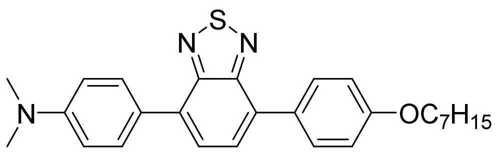

Synthesized from **6a** (0.10 g, 0.30 mmol), **3d** (0.19 g, 0.60 mmol), K_3PO_4 (0.20 g, 0.90 mmol), $Pd(PPh_3)_4$ (0.04 g, 0.03 mmol), and a 5:2:1 (v/v) mixture of toluene/water/methanol (10 ml). Eluting with a hexane/dichloromethane (4:1, v/v) mixture on silica gel column chromatography. Orange solid. Yield: 0.10 g (0.23 mmol), 75%; ^{1}H-NMR (500 MHz, $CDCl_3$) δ 7.91 (m, 4H, Ar**H**), 7.70 (s, 2H, Ar**H**), 7.06 (td, *J* = 2.5, 9.7 Hz, 2H, Ar**H**), 6.90 (d, *J* = 9.2 Hz, 2H, Ar**H**), 4.05 (t, *J* = 6.6 Hz, 2H, OC**H**$_2$), 3.05 (s, 6H, NC**H**$_3$), 1.86-1.80 (m, 2H, C**H**$_2$), 1.52-1.46 (m, 2H, C**H**$_2$), 1.41-1.31 (m, 6H, C**H**$_2$), 0.91 (t, *J* = 6.9 Hz, 3H, C**H**$_3$) ppm (**Figure S59**). ^{13}C-NMR (126 MHz, $CDCl_3$) δ 159.4, 154.5, 150.6, 133.0, 131.6, 130.4, 130.1, 127.7, 126.5, 125.6, 114.8, 112.5, 68.3, 40.6, 32.0, 29.4, 29.2, 26.2, 22.8, 14.2 ppm (**Figure S60).** FT-IR (**Figure S87**). HRMS (EI) Calcd for $C_{27}H_{31}N_3OS$: 445.2188, Found 445.2191 (**Figure S102**).

4-(7-(4-(octyloxy)phenyl)benzo[c][1,2,5]thiadiazol-4-yl)-*N*,*N*-dimethylaniline (Me$_2$NOC8)

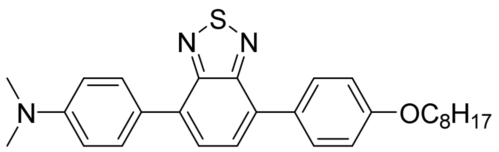

Synthesized from **6a** (0.10 g, 0.30 mmol), **3e** (0.15 g, 0.60 mmol), K_3PO_4 (0.19 g, 0.90 mmol), $Pd(PPh_3)_4$ (0.01 g, 0.01 mmol), and a 5:2:1 (v/v) mixture of toluene/water/methanol (10 ml). Eluting with a hexane/dichloromethane (4:1, v/v) mixture on silica gel column chromatography. Red solid. Yield: 0.14 g (0.28 mmol), 94%; ^{1}H-NMR (500 MHz, $CDCl_3$) δ 7.93-7.89 (m, 4H, Ar**H**), 7.70 (s, 2H, Ar**H**), 7.06 (td, *J* = 2.5, 9.6 Hz, 2H, Ar**H**), 6.89 (d, *J* = 8.9 Hz, 2H, Ar**H**), 4.05 (t, *J* = 6.6 Hz, 2H, OC**H**$_2$), 3.05 (s, 6H, NC**H**$_3$), 1.85-1.80 (m, 2H, C**H**$_2$), 1.52-1.47 (m, 2H, C**H**$_2$), 1.40-1.27 (m, 8H, C**H**$_2$), 0.90 (t, *J* = 7.0 Hz, 3H, C**H**$_3$) ppm (**Figure S61**); ^{13}C-NMR (126 MHz, $CDCl_3$) δ 159.4, 154.5, 150.6, 133.0, 131.6, 130.4, 130.1, 127.7, 126.5, 125.6, 114.8, 112.5, 68.3, 40.6, 32.0, 29.5, 29.4, 29.4, 26.2, 22.8, 14.3 ppm (**Figure S62**). FT-IR (**Figure S88**); HRMS (EI) Calcd for $C_{28}H_{33}N_3OS$: 459.2344, Found 459.2351 (**Figure S103**).

4-(7-(4-((2-ethylhexyl)oxy)phenyl)benzo[c][1,2,5]thiadiazol-4-yl)-*N*,*N*-dimethylaniline (Me$_2$NOEH)

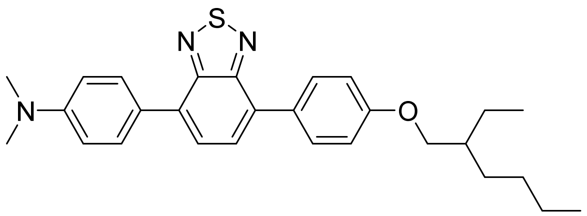

Synthesized from **6a** (0.10 g, 0.30 mmol), **3f** (0.20 g, 0.60 mmol), K_3PO_4 (0.19 g, 0.90 mmol), $Pd(PPh_3)_4$ (0.01 g, 0.01 mmol), and a 5:2:1 (v/v) mixture of toluene/water/methanol (10 ml). Eluting with a hexane/dichloromethane (4:1, v/v) mixture on silica gel column chromatography. Red solid. Yield: 0.08 g (0.17 mmol), 58%; ^{1}H-NMR (500 MHz, $CDCl_3$) δ 7.93-7.89 (m, 4H, Ar**H**), 7.70 (s, 2H, Ar**H**), 7.07 (td, *J* = 2.5, 9.7 Hz, 2H, Ar**H**), 6.90(td, *J* = 2.5, 10.0 Hz, 2H, Ar**H**), 3.96-3.91 (m, 2H, OC**H**$_2$), 3.05 (s, 6H, NC**H**$_3$), 1.80-1.75 (m, 1H, C**H**), 1.58-1.40 (m, 4H, C**H**$_2$), 1.37-1.33 (m, 4H, C**H**$_2$), 0.96 (t, *J* = 7.5 Hz, 3H, C**H**$_3$), 0.92 (t, *J* = 7.0 Hz, 3H, C**H**$_3$) ppm (**Figure S63**). ^{13}C-NMR (126 MHz, $CDCl_3$) δ 159.6,

154.5, 150.6, 133.0, 131.7, 130.4, 130.1, 130.1, 127.7, 126.5, 125.6, 114.8, 112.5, 70.7, 40.6, 39.5, 30.7, 29.2, 24.1, 23.2, 14.3, 11.3 ppm **(Figure S64)**. FT-IR (**Figure S89**); HRMS (EI) Calcd for $C_{28}H_{33}N_3OS$: 459.2344, Found 459.2339 (**Figure S104**).

4-(7-(4-heptylphenyl)benzo[c][1,2,5]thiadiazol-4-yl)-*N*-methyl-*N*-octylaniline (C8MeNC7)

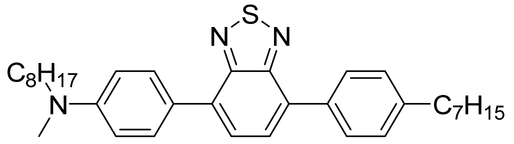

Synthesized from **6b** (0.31 g, 0.80 mmol), **3a** (0.22 g, 0.84 mmol), K_3PO_4 (0.51 g, 2.4 mmol), $Pd(PPh_3)_4$ (0.02 g, 0.02 mmol), and a 5:2:1 (v/v) mixture of toluene/water/methanol (10 ml). Eluting with a hexane/dichloromethane (5:1, v/v) mixture on silica gel column chromatography. Red oil. Yield: 0.05 g (0.09 mmol), 11%; ^{1}H-NMR (500 MHz, $CDCl_3$) δ 7.91 (d, J = 8.9 Hz, 2H, Ar**H**), 7.87 (d, J = 8.2 Hz, 2H, Ar**H**), 7.73 (d, J = 7.3 Hz, 1H, Ar**H**), 7.70 (d, J = 7.3 Hz, 1H, Ar**H**), 7.35 (d, J = 8.2 Hz, 2H, Ar**H**), 6.84 (d, J = 8.9 Hz, 2H, Ar**H**), 3.39 (t, J = 7.5 Hz, 2H, NC**H**$_2$), 3.03 (s, 3H, NC**H**$_3$), 2.69 (t, J = 7.8 Hz, 2H, C**H**$_2$), 1.72-1.62 (m, 4H, C**H**$_2$), 1.41-1.26 (m, 18H, C**H**$_2$), 0.91-0.88 (m, 6H, C**H**$_3$) ppm (**Figure S65**). ^{13}C-NMR (126 MHz, $CDCl_3$) δ 154.5, 154.5, 149.4, 143.1, 135.2, 133.4, 131.7, 130.2, 129.1, 128.8, 128.2, 126.3, 124.7, 112.0, 52.8, 38.5, 36.0, 32.0, 31.6, 29.7, 29.5, 29.5, 29.4, 27.4, 27.0, 22.8, 22.8, 14.3 ppm **(Figure S66)**. FT-IR (**Figure S90**).

***N*-hexyl-4-(7-(4-(hexyloxy)phenyl)benzo[c][1,2,5]thiadiazol-4-yl)aniline (C6NOC6)**

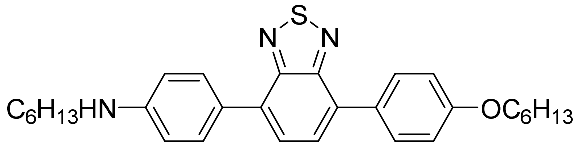

Synthesized from **6c** (0.34 g, 0.87 mmol), **3c** (0.39 g, 1.74 mmol), K_3PO_4 (0.55 g, 2.6 mmol), $Pd(PPh_3)_4$ (0.03 g, 0.03 mmol), and a 5:2:1 (v/v) mixture of toluene/water/methanol (10 ml). Eluting with a hexane/dichloromethane (4:1, v/v) mixture on silica gel column chromatography. Orange solid. Yield: 0.26 g (0.53 mmol), 61%; ^{1}H-NMR (500 MHz, $CDCl_3$) δ 7.90 (td, J = 2.5, 9.7 Hz, 2H, Ar**H**), 7.85 (td, J = 2.3, 9.4 Hz, 2H, Ar**H**), 7.70 (d, J = 7.6 Hz, 1H, Ar**H**), 7.68 (d, J = 7.6 Hz, 1H, Ar**H**), 7.06 (td, J = 2.4, 9.6 Hz, 2H, Ar**H**), 6.76 (td, J = 2.3, 9.4 Hz, 2H, Ar**H**), 4.05 (t, J = 6.6 Hz, 2H, OC**H**$_2$), 3.85 (s, 1H, N**H**), 3.20 (t, J = 7.2 Hz, 2H, NC**H**$_2$), 1.86-1.80 (m, 2H, C**H**$_2$), 1.70-1.64 (m, 2H, C**H**$_2$), 1.51-1.48 (m, 2H, C**H**$_2$), 1.47-1.41 (m, 2H, C**H**$_2$), 1.40-1.33 (m, 8H, C**H**$_2$), 0.94-0.90 (m, 6H, C**H**$_3$) ppm (**Figure S67**). ^{13}C-NMR (126 MHz, $CDCl_3$) δ 159.4, 154.5, 154.4, 148.8, 133.0, 131.6, 130.4, 130.3, 130.1, 127.7, 126.5, 126.2, 114.8, 112.7, 68.3, 44.0, 31.8, 31.8, 29.7, 29.4, 27.0, 25.9, 22.8, 14.2 ppm (**Figure S68**). FT-IR (**Figure S91**); HRMS (EI) Calcd for $C_{30}H_{37}N_3OS$: 487.2657, Found 487.2659 (**Figure S105**).

***N*-(2-ethylhexyl)-4-(7-(4-(hexyloxy)phenyl)benzo[c][1,2,5]thiadiazol-4-yl)aniline (EHNOC6)**

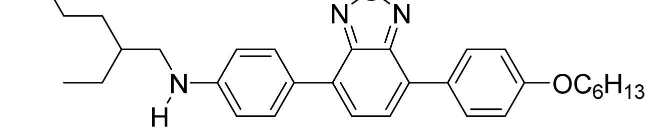

Synthesized from **6d** (0.16 g, 0.38 mmol), **3c** (0.17 g, 0.76 mmol), K_3PO_4 (0.25 g, 1.14 mmol), $Pd(PPh_3)_4$ (0.013 g, 0.011 mmol), and a 5:2:1 (v/v) mixture of toluene/water/methanol (10 ml). Eluting with a hexane/dichloromethane (1:1, v/v) mixture on silica gel column chromatography. Yellow solid.

Yield: 0.08 g (0.17 mmol), 45%; ^{1}H-NMR (500 MHz, $CDCl_3$) δ 7.90 (td, J = 2.5, 9.7 Hz, 2H, Ar**H**), 7.85 (td, J = 2.3, 9.4 Hz, 2H, Ar**H**), 7.70 (d, J = 7.3 Hz, 1H, Ar**H**), 7.68 (d, J = 7.3 Hz, 1H, Ar**H**), 7.06 (td, J = 2.5, 9.6 Hz, 2H, Ar**H**), 6.76 (td, J = 2.3, 9.5 Hz, 2H, Ar**H**), 4.05 (t, J = 6.6 Hz, 2H, OC**H**$_2$), 3.87 (s, 1H, N**H**), 3.11 (d, J = 6.1 Hz, 2H, NC**H**$_2$), 1.86-1.80 (m, 2H, C**H**$_2$), 1.67-1.59 (m, 1H, C**H**), 1.51-1.48 (m, 2H, C**H**$_2$), 1.46-1.41 (m, 2H, C**H**$_2$), 1.40-1.30 (m, 10H, C**H**$_2$), 0.96-0.91 (m, 9H, C**H**$_3$) ppm (**Figure S69**). ^{13}C-NMR (126 MHz, $CDCl_3$) δ 159.4, 154.5, 154.5, 149.0, 133.1, 131.6, 130.4, 130.4, 130.2, 127.7, 126.5, 126.1, 114.8, 112.6, 68.3, 47.0, 39.2, 31.8, 31.5, 29.4, 29.2, 25.9, 24.6, 23.3, 22.8, 14.3, 14.2, 11.1 ppm **(Figure S70)**. FT-IR (**Figure S92**); HRMS (EI) Calcd for $C_{32}H_{41}N_3OS$: 515.2970, Found 525.2978 (**Figure S106**).

***N*-(2-ethylhexyl)-4-(7-(4-((2-ethylhexyl)oxy)phenyl)benzo[c][1,2,5]thiadiazol-4-yl)aniline (EHNOEH)**

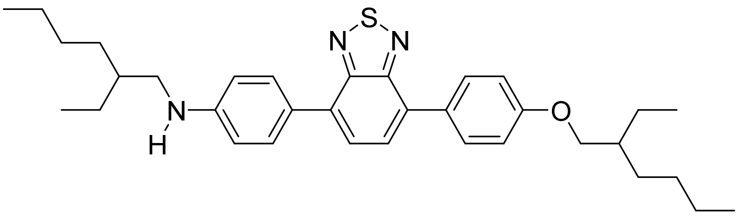

Synthesized from **6d** (0.84 g, 2.0 mmol), **3f** (1.0 g, 4.0 mmol), K_3PO_4 (1.27 g, 6.0 mmol), $Pd(PPh_3)_4$ (0.07 g, 0.06 mmol), and a 5:2:1 (v/v) mixture of toluene/water/methanol (10 ml). Eluting with a hexane/dichloromethane (3:1, v/v) mixture on silica gel column chromatography. Red oil. Yield: 1.07 g (1.92 mmol), 96%; ^{1}H-NMR (500 MHz, $CDCl_3$) δ 7.90 (td, J = 2.5, 9.7 Hz, 2H, Ar**H**), 7.85 (td, J = 2.3, 9.4 Hz, 2H, Ar**H**), 7.69 (d, J = 7.3 Hz, 1H, Ar**H**), 7.68 (d, J = 7.3 Hz, 1H, Ar**H**), 7.07 (td, J = 2.5, 9.7 Hz, 2H, Ar**H**), 6.76 (td, J = 2.3, 9.5 Hz, 2H, Ar**H**), 3.96-3.91 (m, 2H, OC**H**$_2$), 3.87 (s, 1H, N**H**), 3.11 (d, J = 6.1 Hz, 2H, NC**H**$_2$), 1.80-1.74 (m, 1H, C**H**), 1.65-1.60 (m, 1H, C**H**), 1.52-1.32 (m, 16H, C**H**$_2$), 0.97-0.91 (m, 12H, C**H**$_3$) ppm (**Figure S71**). ^{13}C-NMR (126 MHz, $CDCl_3$) δ 159.6, 154.5, 154.4, 149.0, 133.0, 131.6, 130.4, 130.3, 130.0, 127.6, 126.4, 126.1, 114.8, 112.6, 70.7, 47.0, 39.5, 39.2, 31.4, 30.7, 29.2, 29.1, 24.6, 24.0, 23.3, 23.2, 14.2, 11.3, 11.1 ppm (**Figure S72**). FT-IR (**Figure S93**).

4-(7-(4-octylphenyl)benzo[c][1,2,5]thiadiazol-4-yl)benzaldehyde (AldC8)

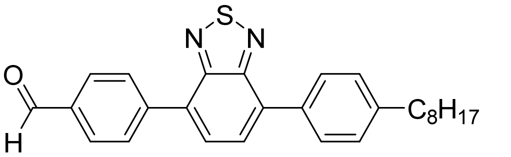

Synthesized from **6f** (0.18 g, 0.6 mmol), **3b** (0.14 g, 1.2 mmol), K_3PO_4 (0.38 g, 1.8 mmol), $Pd(PPh_3)_4$ (0.02 g, 0.02 mmol), and a 5:2:1 (v/v) mixture of toluene/water/methanol (10 ml). Eluting with a hexane/dichloromethane (1:1, v/v) mixture on silica gel column chromatography. Yellow solid. Yield: 0.12 g (0.28 mmol), 46%; ^{1}H-NMR (500 MHz, $CDCl_3$) δ 10.12 (s, 1H, C**H**O), 8.17 (d, J = 8.2 Hz, 2H, Ar**H**), 8.06 (td, J = 1.8, 8.4 Hz, 2H, Ar**H**), 7.90 (td, J = 1.9, 8.5 Hz, 2H, Ar**H**), 7.87 (d, J = 7.3 Hz, 1H, Ar**H**), 7.81 (d, J = 7.3 Hz, 1H, Ar**H**), 7.38 (d, J = 8.2 Hz, 2H, Ar**H**), 2.71 (t, J = 7.6 Hz, 2H, C**H**$_2$), 1.72-1.66 (m, 2H, C**H**$_2$), 1.43-1.24 (m, 10H, C**H**$_2$), 0.89 (t, J = 7.0 Hz, 3H, C**H**$_3$) ppm (**Figure S73**). ^{13}C-NMR (126 MHz, $CDCl_3$) δ 192.0, 154.3, 153.9, 143.9, 143.5, 135.9, 134.8, 134.5, 131.5, 130.1, 130.0, 129.3, 129.1, 128.9, 127.7, 36.0, 32.1, 31.6, 29.7, 29.6, 29.4, 22.8, 14.3 ppm (**Figure S74**). FT-IR (**Figure S94**); HRMS (EI) Calcd for $C_{27}H_{28}N_2OS$: 428.1922, Found 428.1925 (**Figure S107**).

1-(4-(7-(4-octylphenyl)benzo[c][1,2,5]thiadiazol-4-yl)phenyl)ethan-1-one (ActC8)

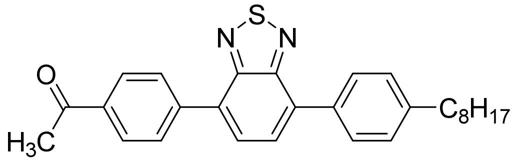

Synthesized from **6g** (0.16 g, 0.51 mmol), **3b** (0.24 g, 1.02 mmol), K_3PO_4 (0.33 g, 1.53 mmol), $Pd(PPh_3)_4$ (0.02 g, 0.02 mmol), and a 5:2:1 (v/v) mixture of toluene/water/methanol (10 ml). Eluting with a hexane/dichloromethane (1:1, v/v) mixture on silica gel column chromatography. Green solid. Yield: 0.22 g (0.49 mmol), 97%; ^{1}H-NMR (500 MHz, $CDCl_3$) δ 8.15-8.13 (m, 2H, Ar**H**), 8.10-8.09 (m, 2H, Ar**H**), 7.90-7.89 (m, 2H, Ar**H**), 7.85 (d, *J* = 7.3 Hz, 1H, Ar**H**), 7.80 (d, *J* = 7.3 Hz, 1H, Ar**H**), 7.38 (d, *J* = 8.2 Hz, 2H, Ar**H**), 2.71 (t, *J* = 7.9 Hz, 2H, C**H**$_2$), 2.68 (s, 3H, C**H**$_3$), 1.73-1.67 (m, 2H, C**H**$_2$), 1.43-1.26 (m, 10H, C**H**$_2$), 0.89 (t, *J* = 6.9 Hz, 3H, C**H**$_3$) ppm (**Figure 75**). ^{13}C-NMR (126 MHz, $CDCl_3$) δ 197.8, 154.3, 154.0, 143.9, 142.2, 136.7, 134.6, 134.6, 131.7, 129.5, 129.3, 128.9, 128.9, 128.8, 127.7, 36.0, 32.1, 31.6, 29.7, 29.6, 29.4, 26.9, 22.8, 14.3 ppm (**Figure S76**). FT-IR (**Figure S95**); HRMS (EI) Calcd for $C_{28}H_{30}N_2OS$: 442.2079, Found 442.2090 (**Figure S108**).

2,2,2-trifluoro-1-(4-(7-(4-octylphenyl)benzo[c][1,2,5]thiadiazol-4-yl)phenyl)ethan-1-one (TFActC8)

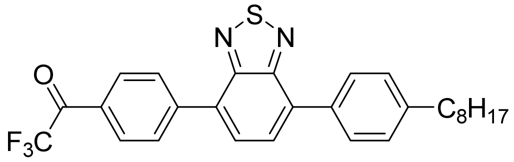

Synthesized from **6h** (0.20 g, 0.59 mmol), **3b** (0.14 g, 1.2 mmol), K_3PO_4 (0.38 g, 1.8 mmol), $Pd(PPh_3)_4$ (0.02 g, 0.02 mmol), and a 5:2:1 (v/v) mixture of toluene/water/methanol (10 ml). Eluting with a hexane/dichloromethane (4:1, v/v) mixture on silica gel column chromatography. Green oil. Yield: 0.13 g (0.26 mmol), 44 %; ^{1}H-NMR (500 MHz, $CDCl_3$) δ 8.26 (d, *J* = 7.9 Hz, 2H, Ar**H**), 8.20 (td, *J* = 1.8, 8.5 Hz, 2H, Ar**H**), 7.91-7.88 (m, 3H, Ar**H**), 7.82 (d, *J* = 7.3 Hz, 1H, Ar**H**), 7.38 (d, *J* = 7.9 Hz, 2H, Ar**H**), 2.71 (t, *J* = 7.6 Hz, 2H, C**H**$_2$), 1.73-1.67 (m, 2H, C**H**$_2$), 1.43-1.26 (m, 10H, C**H**$_2$), 0.89 (t, *J* = 7.0 Hz, 3H, C**H**$_3$ ppm (**Figure S77**). ^{13}C-NMR (126 MHz, $CDCl_3$) δ 180.4, 180.1, 154.3, 153.8, 144.6, 144.1, 135.3, 134.5, 130.9, 130.6, 129.9, 129.3, 129.3, 129.0, 127.7, 118.1, 115.8, 36.0, 32.1, 31.6, 29.7, 29.6, 29.4, 22.8, 14.3 ppm (**Figure S78**). FT-IR (**Figure S96**); HRMS (EI) Calcd for $C_{28}H_{27}F_3N_2OS$: 496.1796, Found 496.1806 (**Figure S109**).

4-(4-octylphenyl)-7-(4-(trifluoromethyl)phenyl)benzo[c][1,2,5]thiadiazole (TFMeC8)

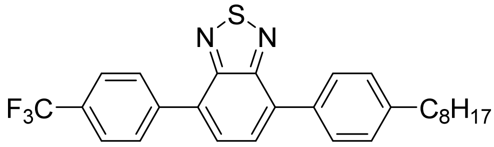

Synthesized from **6i** (0.37 g, 1.0 mmol), **3b** (0.23 g, 2.0 mmol), K_3PO_4 (0.64 g, 3.0 mmol), $Pd(PPh_3)_4$ (0.04 g, 0.03 mmol), and a 5:2:1 (v/v) mixture of toluene/water/methanol (10 ml). Eluting with a hexane/dichloromethane (8:1, v/v) mixture on silica gel column chromatography. Green solid. Yield: 0.05 g (0.11 mmol), 11%; ^{1}H-NMR (500 MHz, $CDCl_3$) δ 8.09 (d, *J* = 7.9 Hz, 2H, Ar**H**), 7.89 (td, *J* = 1.9, 8.5 Hz, 2H, Ar**H**), 7.83-7.79 (m, 4H, Ar**H**), 7.38 (d, *J* = 8.2 Hz, 2H, Ar**H**), 2.71 (t, *J* = 7.8 Hz, 2H, C**H**$_2$), 1.73-1.67 (m, 2H, C**H**$_2$), 1.43-1.25 (m, 10H, C**H**$_2$), 0.89 (t, *J* = 6.9 Hz, 3H, C**H**$_3$) ppm (**Figure S79**). ^{13}C-NMR (126 MHz, $CDCl_3$) δ 154.3, 154.0, 143.9, 141.1, 134.6, 134.6, 131.5, 130.4, 130.2, 129.7, 129.3, 129.2, 128.9, 128.9, 128.0, 127.7, 125.7, 125.7, 125.4, 123.3, 36.0, 32.1, 31.6, 29.7, 29.6,

29.4, 22.8, 14.3 ppm (**Figure S80**). FT-IR (**Figure S97**); HRMS (EI) Calcd for $C_{27}H_{27}F_3N_2S$: 468.1847, Found 468.1841 (**Figure S110**).

4-(7-(4-(hexylamino)phenyl)benzo[c][1,2,5]thiadiazol-4-yl)benzonitrile (C6NCN)

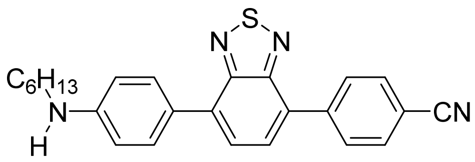

Synthesized from **6e** (0.17 g, 0.54 mmol), **5a** (0.24 g, 0.81 mmol), K_3PO_4 (0.34 g, 1.62 mmol), $Pd(PPh_3)_4$ (0.018 g, 0.016 mmol), and a 5:2:1 (v/v) mixture of toluene/water/methanol (10 ml). Eluting with a hexane/dichloromethane (1:1, v/v) mixture on silica gel column chromatography. Red solid. Yield: 0.08 g (0.19 mmol), 36%; ^{1}H-NMR (500 MHz, $CDCl_3$) δ 8.11 (td, *J* = 1.7, 8.4 Hz, 2H, Ar**H**), 7.88 (td, *J* = 2.4, 9.7 Hz, 2H, Ar**H**), 7.83-7.81 (m, *J* = 6.9, 1.7 Hz, 2H, Ar**H**), 7.80 (d, *J* = 7.3 Hz, 1H, Ar**H**), 7.74 (d, *J* = 7.3 Hz, 1H, Ar**H**), 6.76 (td, *J* = 2.2, 9.4 Hz, 2H, Ar**H**), 3.91 (s, 1H, N**H**), 3.23-3.19 (m, 2H, NC**H**$_2$), 1.70-1.65 (m, 2H, C**H**$_2$), 1.46-1.41 (m, 2H, C**H**$_2$), 1.36-1.33 (m, 4H, C**H**$_2$), 0.92 (t, *J* = 7.0 Hz, 3H, C**H**$_3$) ppm (**Figure S81**). ^{13}C-NMR (126 MHz, $CDCl_3$) δ 154.3, 153.9, 149.2, 142.3, 135.3, 132.4, 130.6, 129.8, 129.4, 129.3, 126.0, 125.5, 119.1, 112.7, 111.5, 43.9, 31.8, 29.6, 27.0, 22.8, 14.2 ppm (**Figure S82**). FT-IR (**Figure S98**); HRMS (EI) Calcd for $C_{25}H_{24}N_4S$: 412.1722, Found 412.1713 (**Figure S111**).

***N*-hexyl-4-(7-(4-(trifluoromethyl)phenyl)benzo[c][1,2,5]thiadiazol-4-yl)aniline (C6NTFMe)**

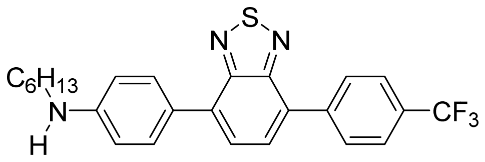

Synthesized from **6i** (0.25 g, 0.70 mmol), **5a** (0.42 g, 1.4 mmol), K_3PO_4 (0.34 g, 2.1 mmol) and $Pd(PPh_3)_4$ (0.02 g, 0.02 mmol) was dissolved in a 5:2:1 (v/v) mixture of toluene/water/methanol (10 ml). Eluting with a hexane/dichloromethane (1:1, v/v) mixture on silica gel column chromatography. Yield: 0.14 g (0.31 mmol), 43%; ^{1}H-NMR (500 MHz, $CDCl_3$) δ 8.08 (d, *J* = 7.9 Hz, 2H, Ar**H**), 7.88 (td, *J* = 2.4, 9.4 Hz, 2H, Ar**H**), 7.80-7.78 (m, 3H, Ar**H**), 7.73 (d, *J* = 7.3 Hz, 1H, Ar**H**), 6.77 (td, *J* = 2.4, 9.7 Hz, 2H, Ar**H**), 3.89 (s, 1H, N**H**), 3.21 (t, *J* = 7.2 Hz, 2H, NC**H**$_2$), 1.70-1.65 (m, 2H, C**H**$_2$), 1.47-1.41 (m, 2H, C**H**$_2$), 1.37-1.33 (m, 4H, C**H**$_2$), 0.92 (t, *J* = 7.0 Hz, 3H, C**H**$_3$) ppm (**Figure S83**). ^{13}C-NMR (126 MHz, $CDCl_3$) δ 154.3, 154.1, 149.1, 141.3, 134.8, 130.5, 130.1, 130.1, 129.9, 129.5, 129.1, 126.1, 125.7, 125.7, 125.6, 125.6, 125.6, 125.5, 112.7, 44.0, 31.8, 29.7, 27.0, 22.8, 14.2 ppm (**Figure S84**). FT-IR (**Figure S99**); HRMS (EI) Calcd for $C_{25}H_{24}F_3N_3S$: 455.1643, Found 455.1641 (**Figure S112**).

Spectra chart

NMR chart

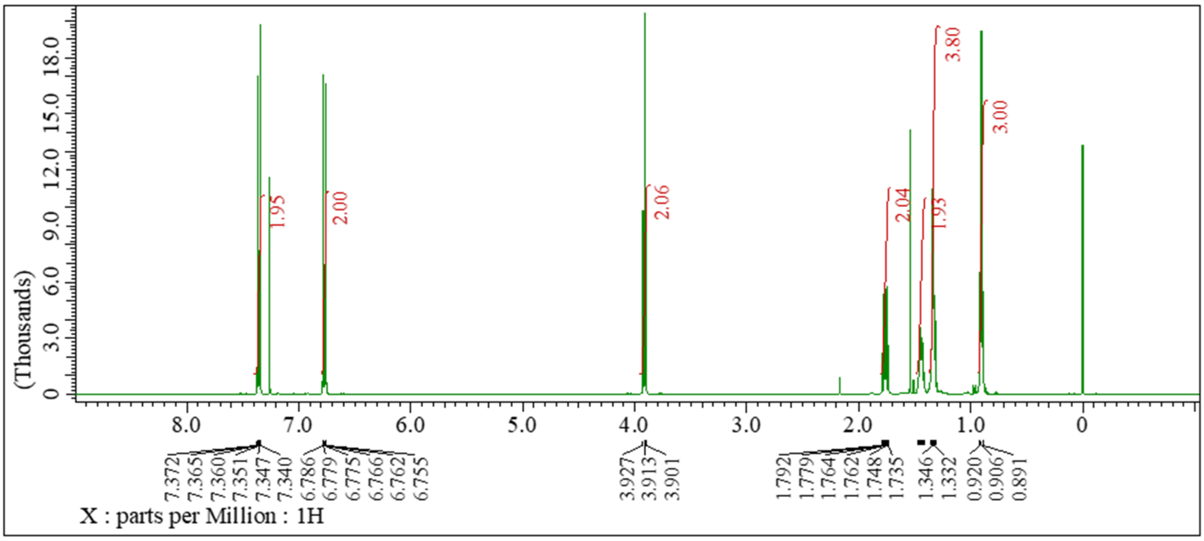

Figure S36. ^{1}H-NMR spectrum of **1a** (500 MHz, $CDCl_3$).

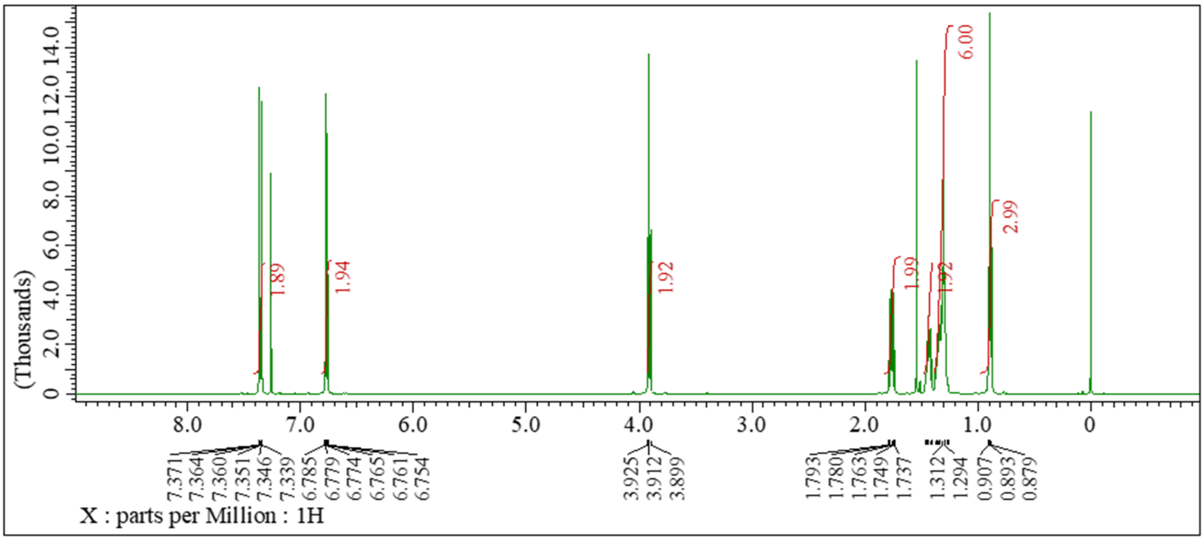

Figure S37. ^{1}H-NMR spectrum of **1b** (500 MHz, $CDCl_3$).

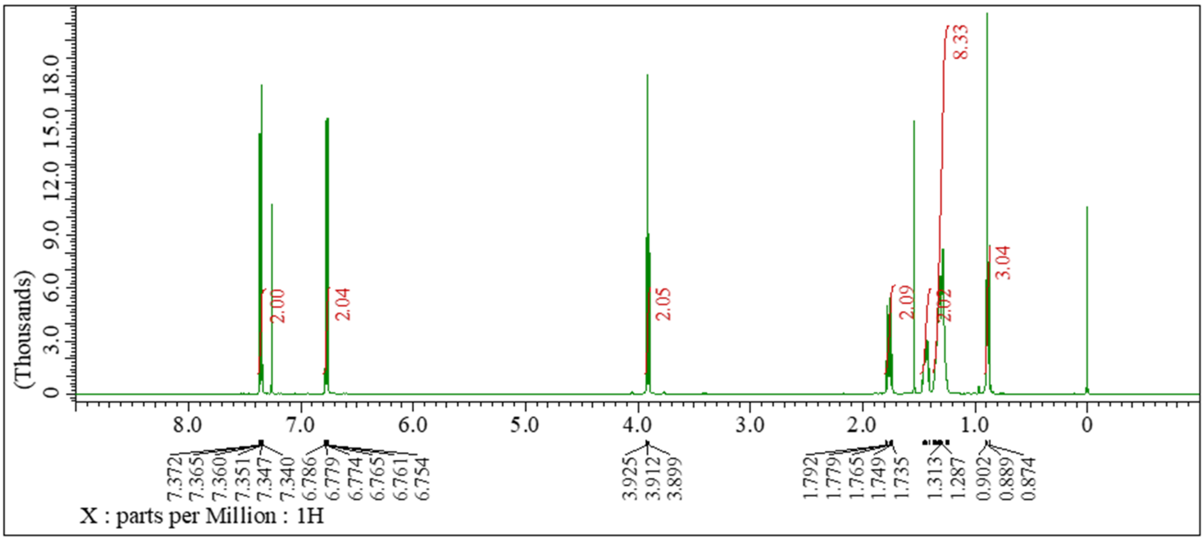

Figure S38. ^{1}H-NMR spectrum of **1c** (500 MHz, $CDCl_3$).

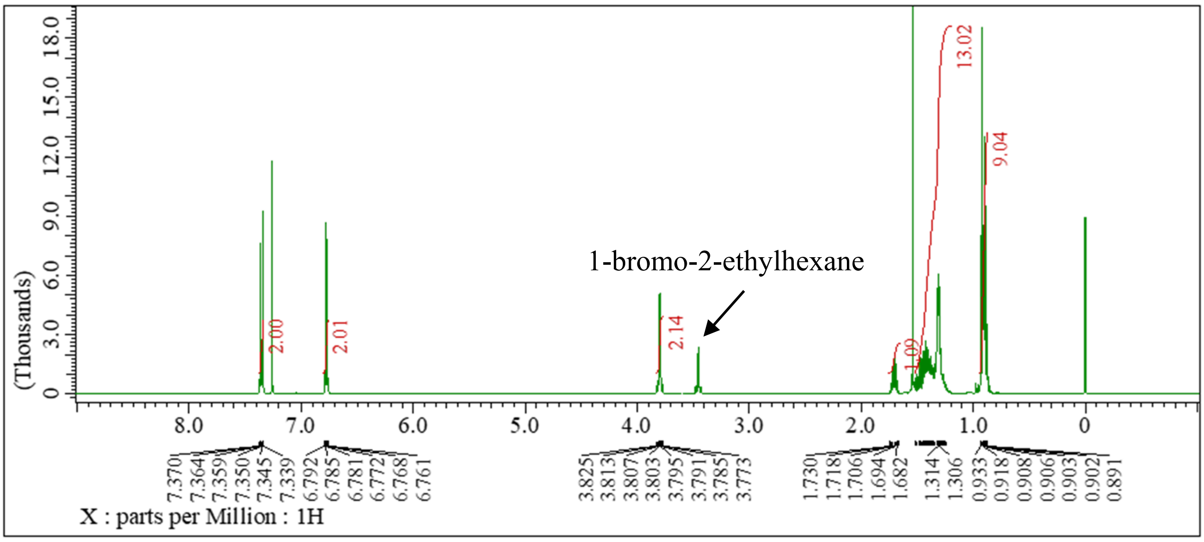

Figure S39. ^{1}H-NMR spectrum of **1d** (500 MHz, $CDCl_3$).

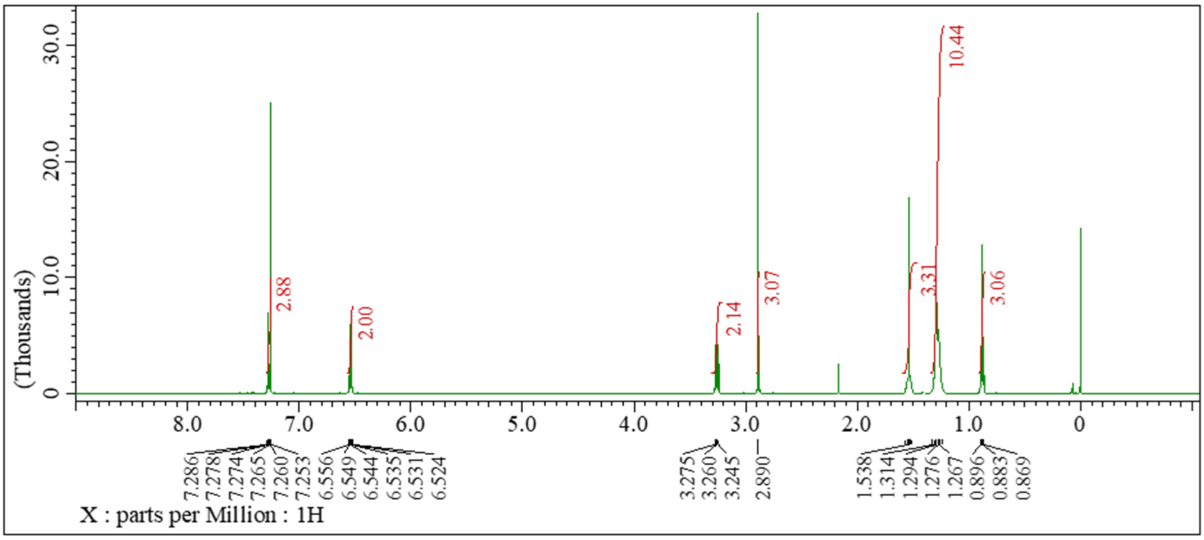

Figure S40. ^{1}H-NMR spectrum of **2** (500 MHz, $CDCl_3$).

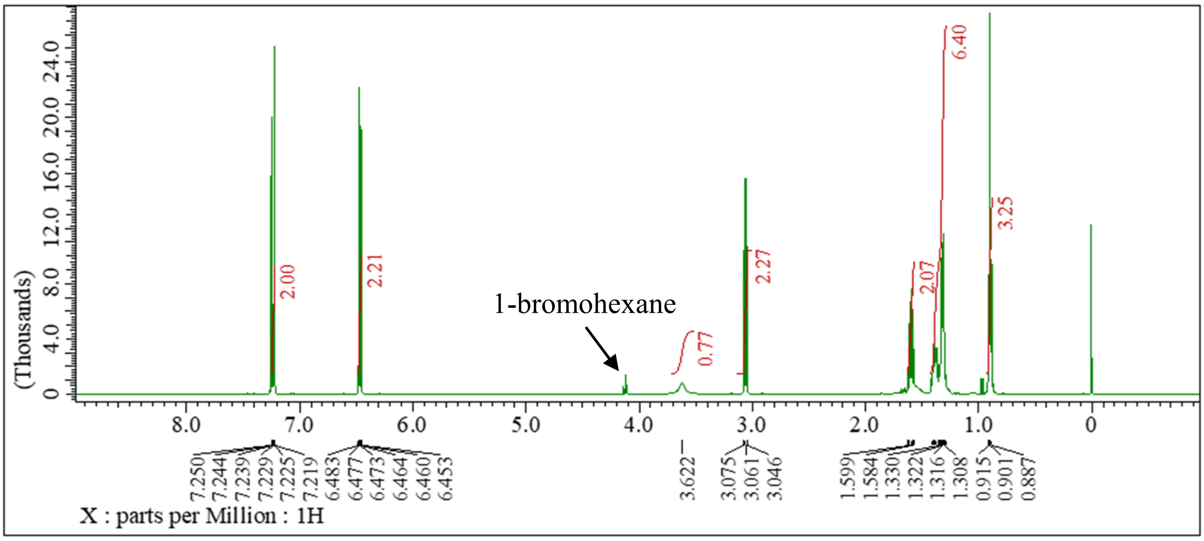

Figure S41. ^{1}H-NMR spectrum of **4a** (500 MHz, $CDCl_3$).

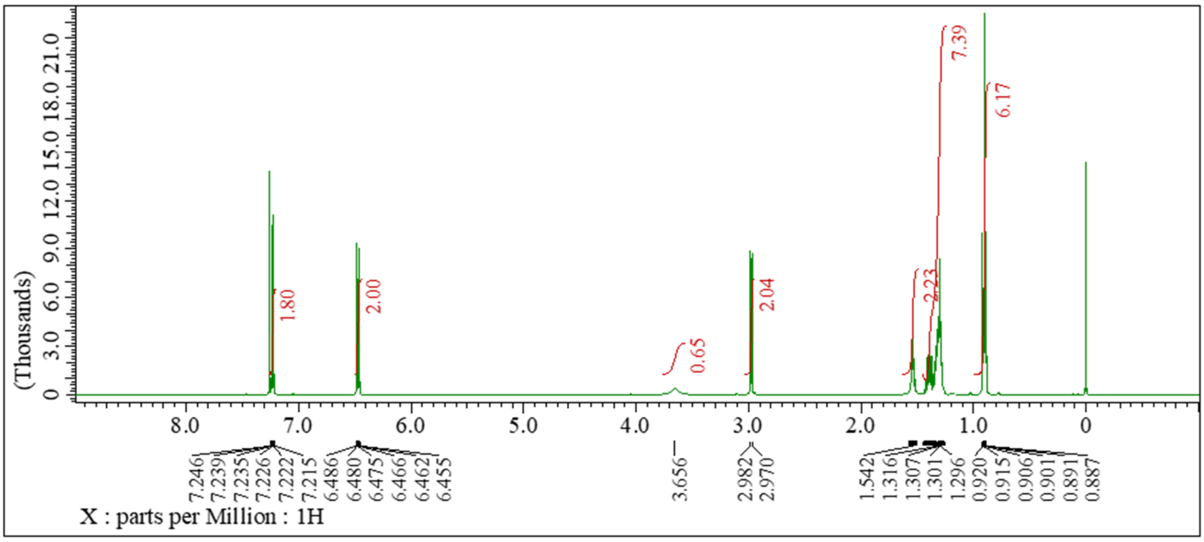

Figure S42. ^{1}H-NMR spectrum of **4b** (500 MHz, $CDCl_3$).

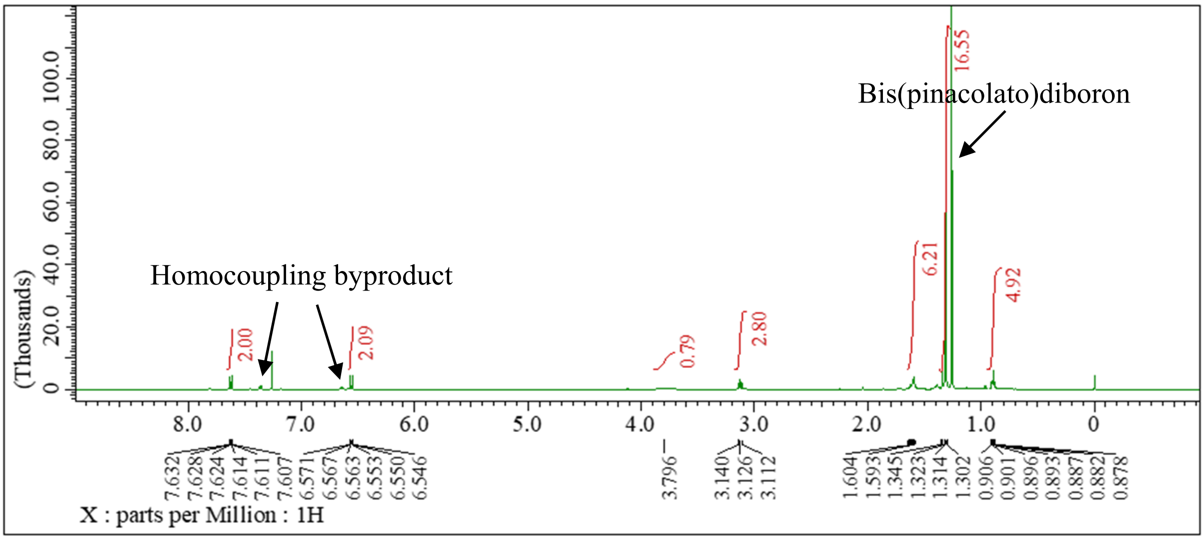

Figure S43. ^{1}H-NMR spectrum of **5a** (500 MHz, $CDCl_3$).

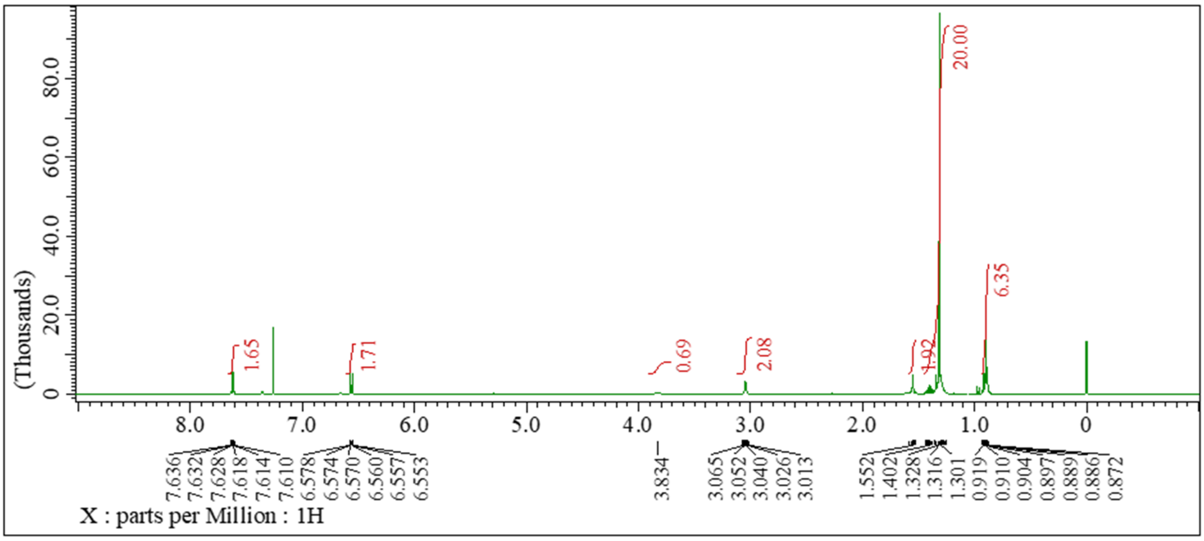

Figure S44. ^{1}H-NMR spectrum of **5b** (500 MHz, $CDCl_3$).

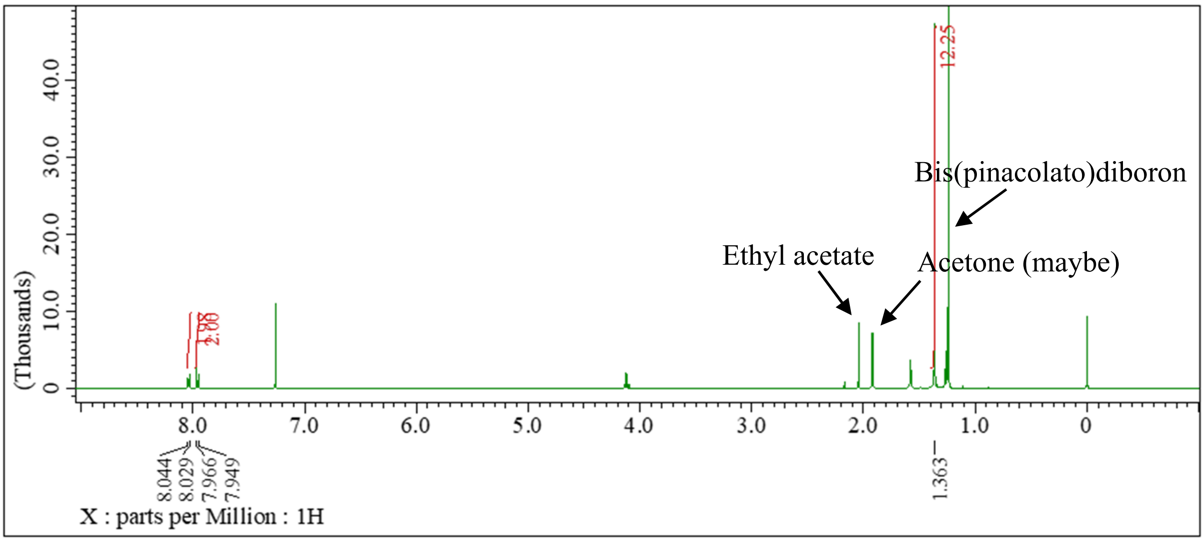

Figure S45. ^{1}H-NMR spectrum of **5c** (500 MHz, $CDCl_3$).

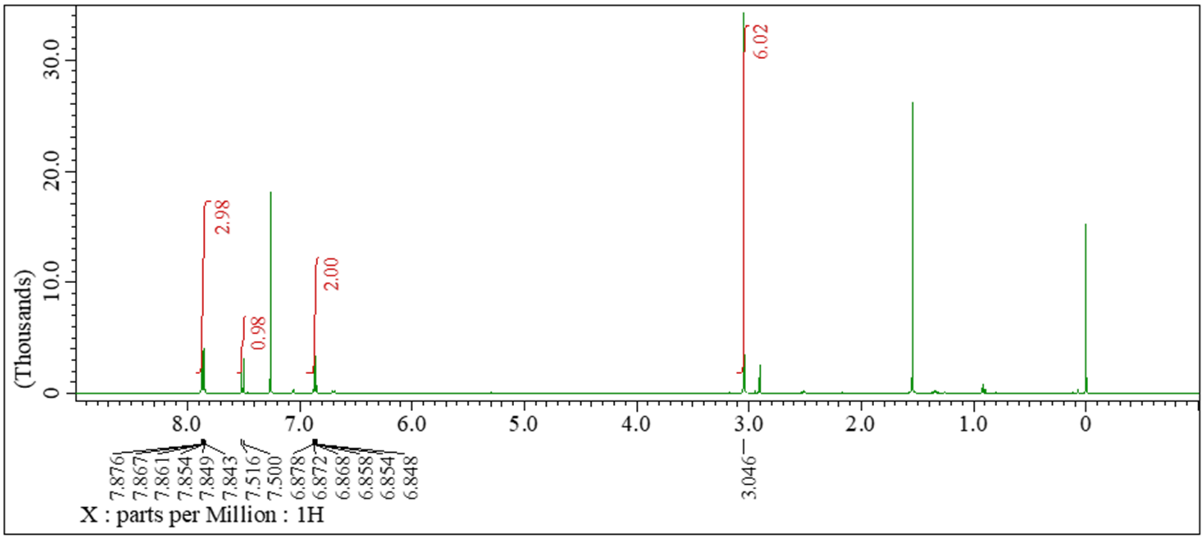

Figure S46. ^{1}H-NMR spectrum of **6a** (500 MHz, $CDCl_3$).

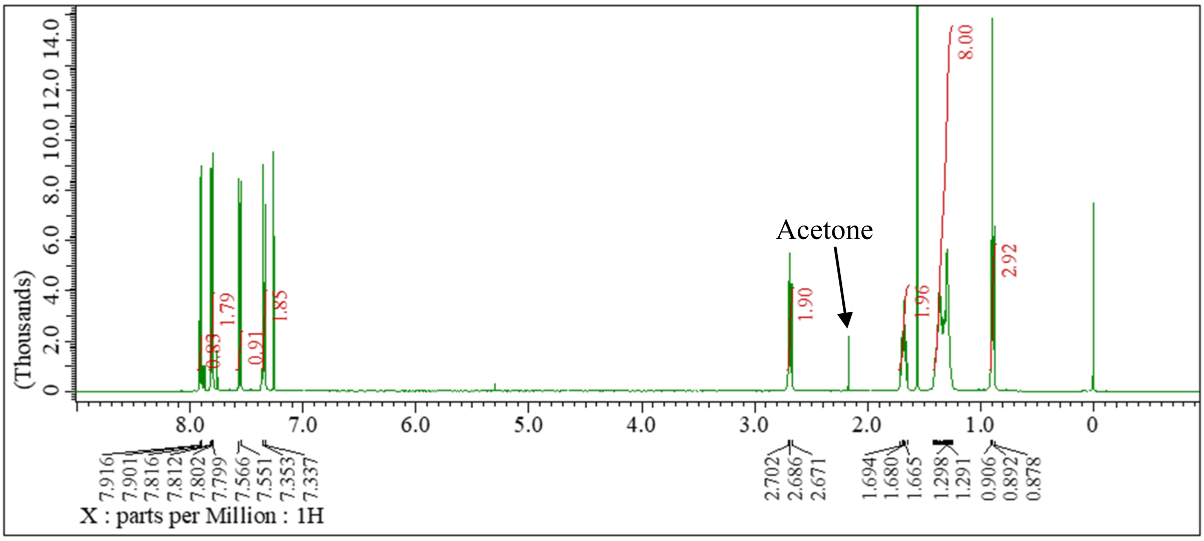

Figure S47. ^{1}H-NMR spectrum of **6b** (500 MHz, $CDCl_3$).

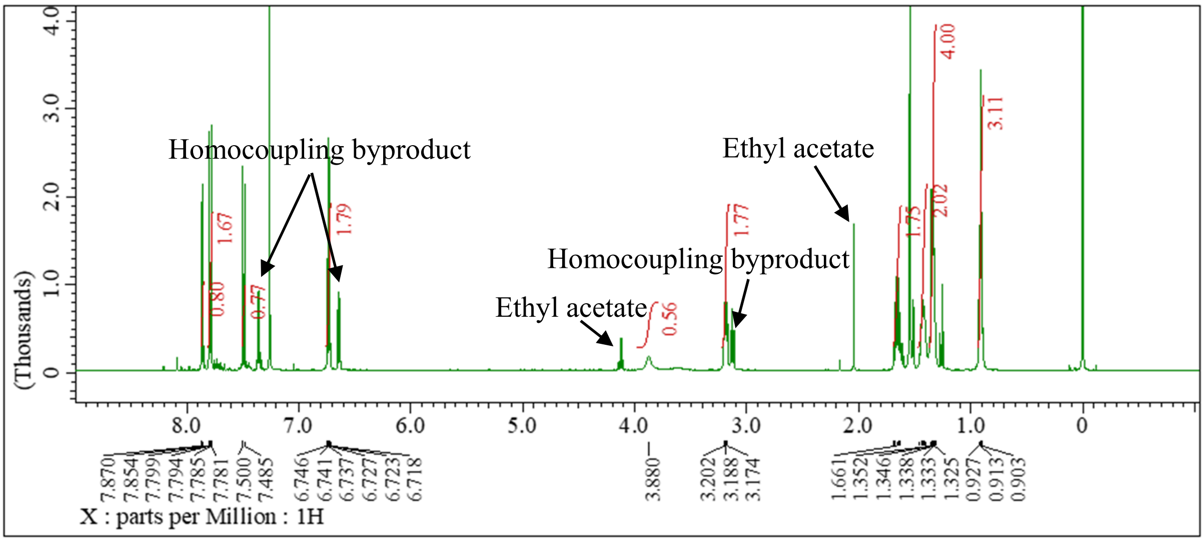

Figure S48. ^{1}H-NMR spectrum of **6c** (500 MHz, $CDCl_3$).

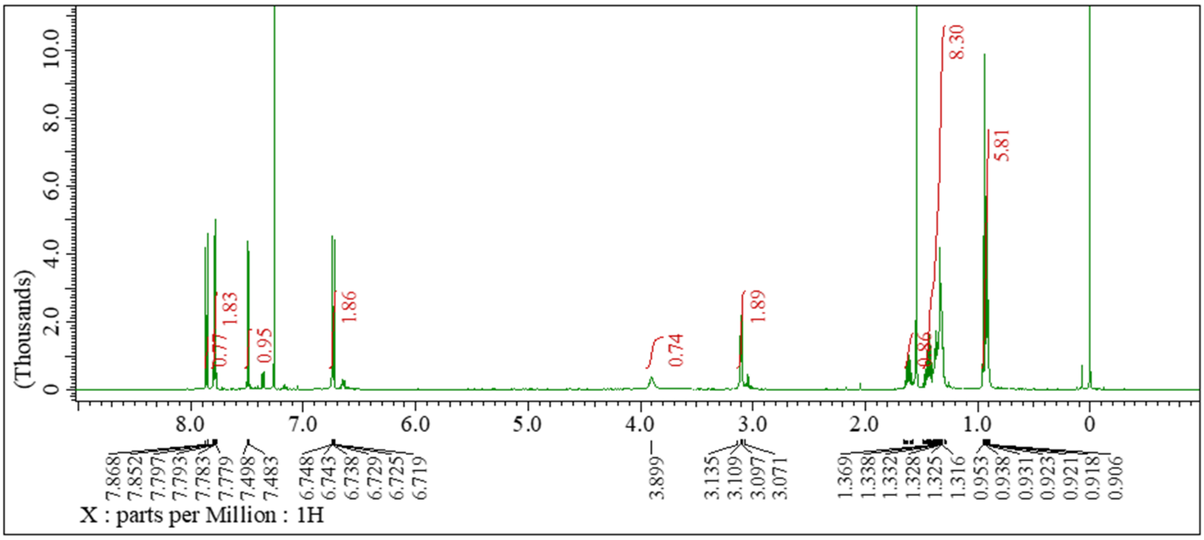

Figure S49. ^{1}H-NMR spectrum of **6d** (500 MHz, $CDCl_3$).

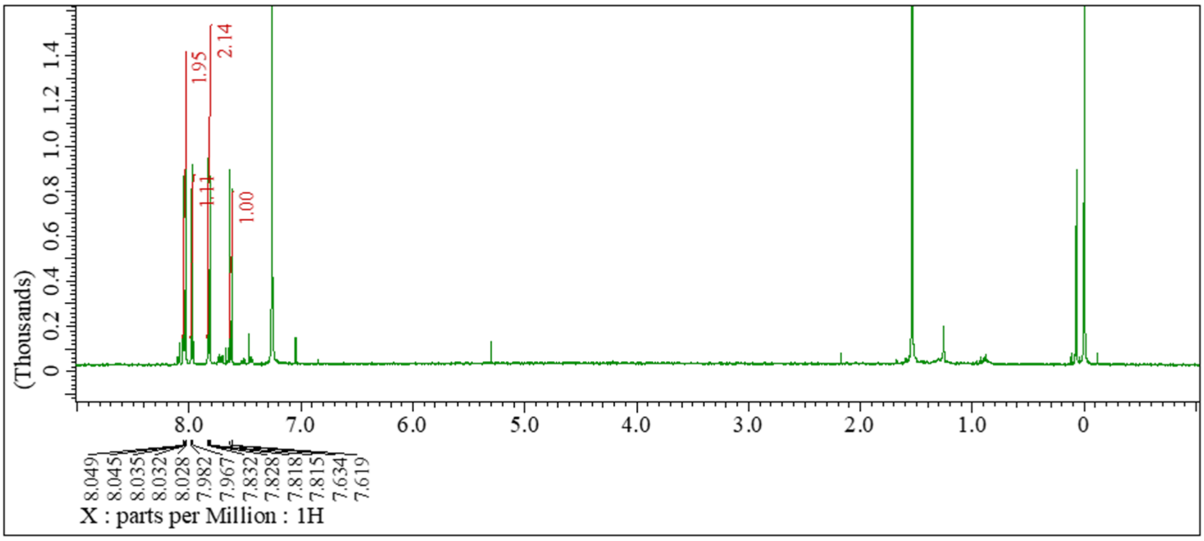

Figure S50. ^{1}H-NMR spectrum of **6e** (500 MHz, $CDCl_3$).

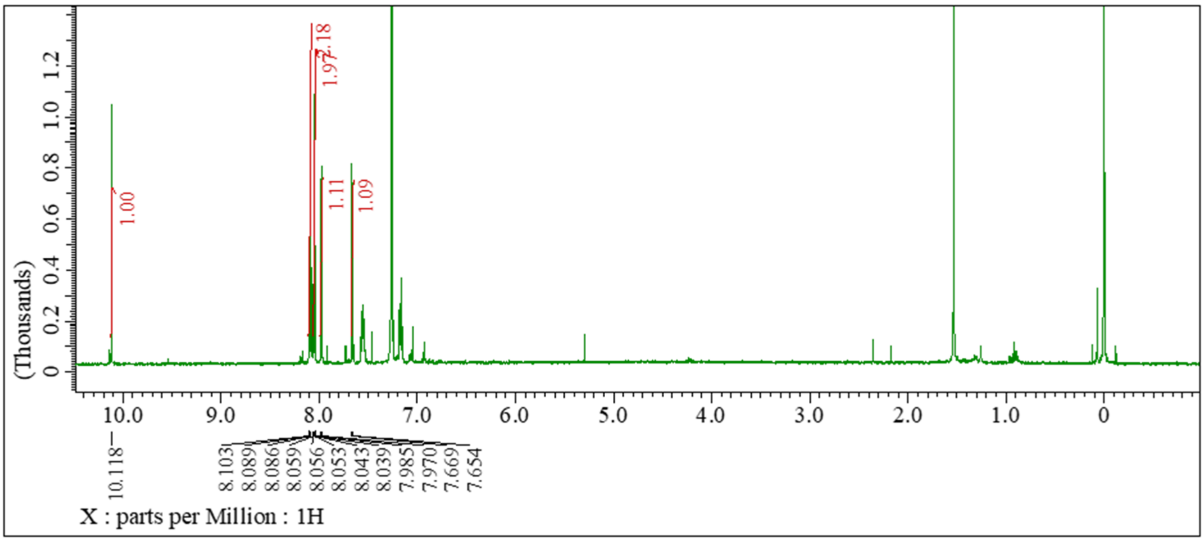

Figure S51. ^{1}H-NMR spectrum of **6f** (500 MHz, $CDCl_3$).

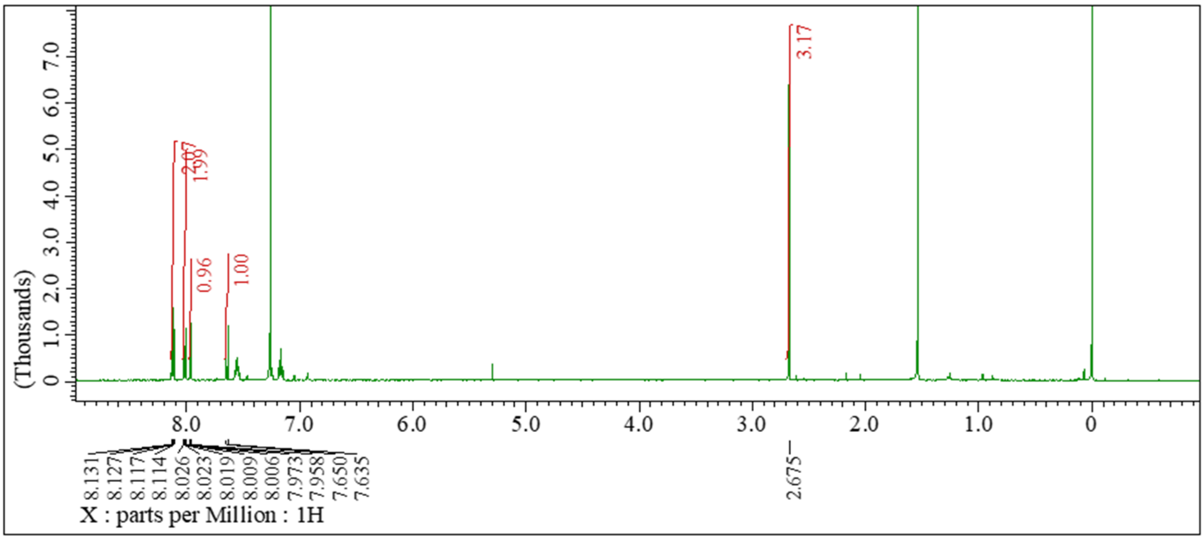

Figure S52. ^{1}H-NMR spectrum of **6g** (500 MHz, $CDCl_3$).

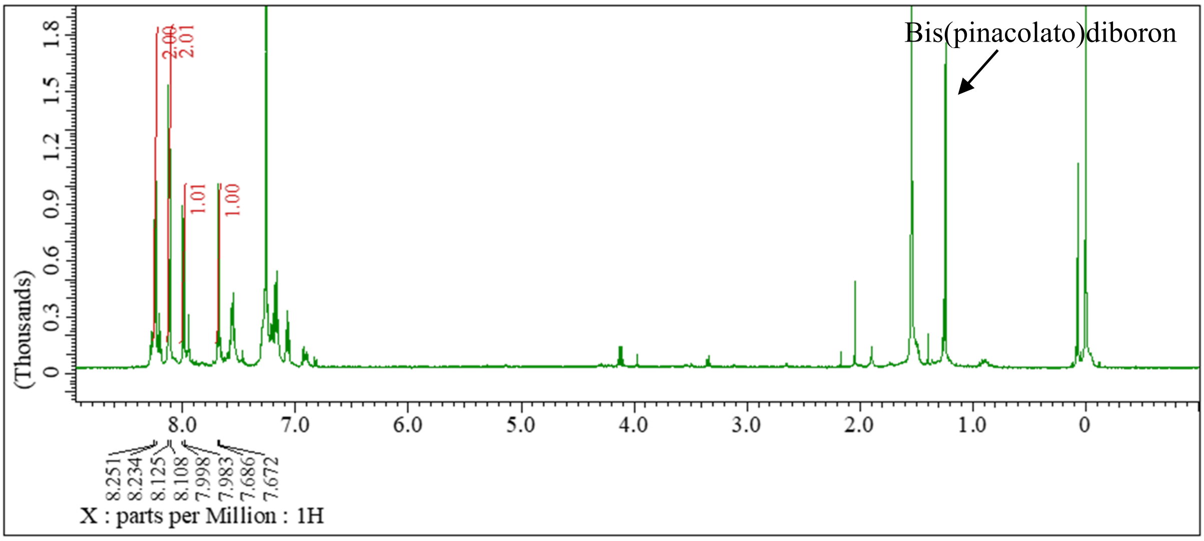

Figure S53. ^{1}H-NMR spectrum of **6h** (500 MHz, $CDCl_3$).

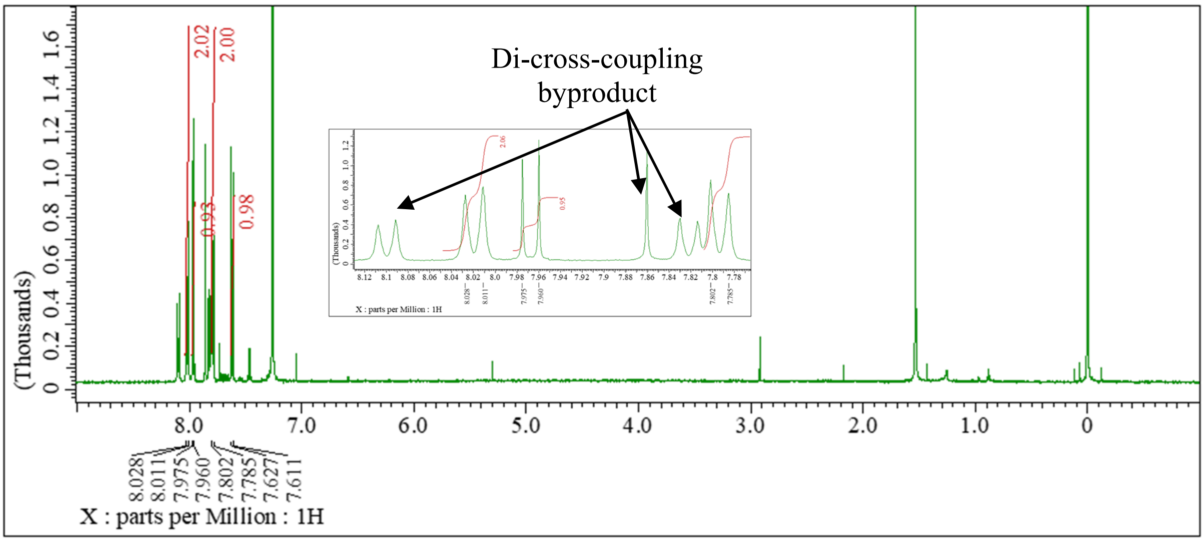

Figure S54. ^{1}H-NMR spectrum of **6i** (500 MHz, $CDCl_3$).

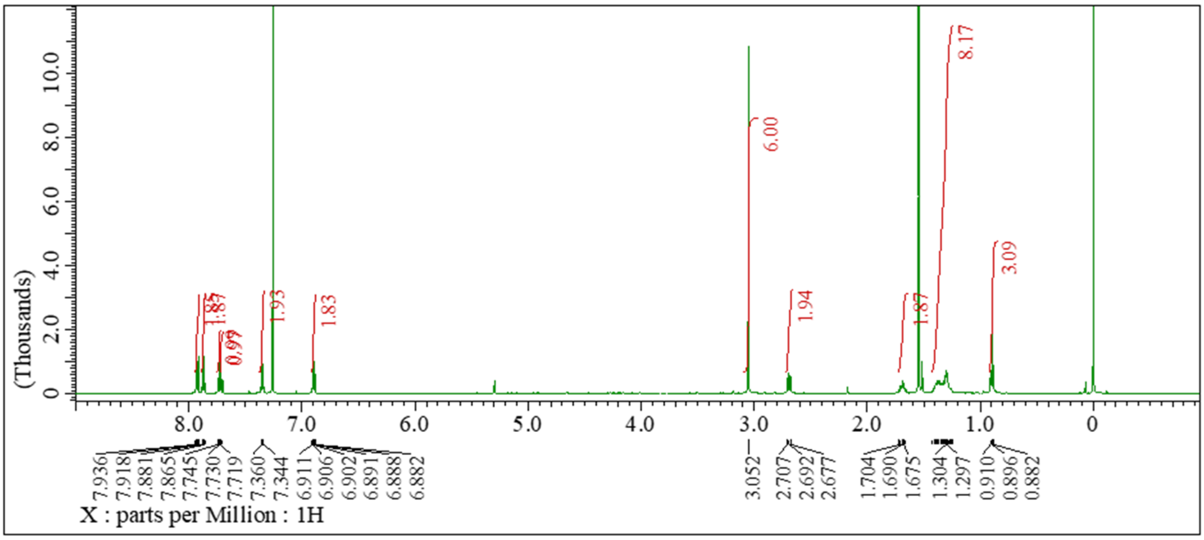

Figure S55. ^{1}H-NMR spectrum of **Me$_2$NC7** (500 MHz, $CDCl_3$).

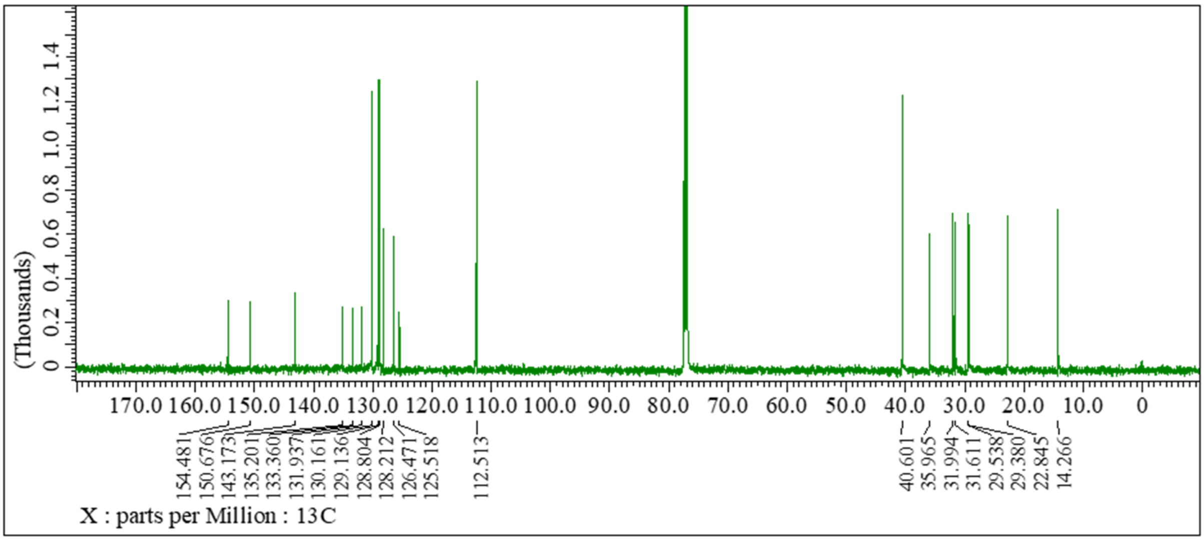

Figure S56. ^{13}C-NMR spectrum of **Me$_2$NC7** (126 MHz, $CDCl_3$).

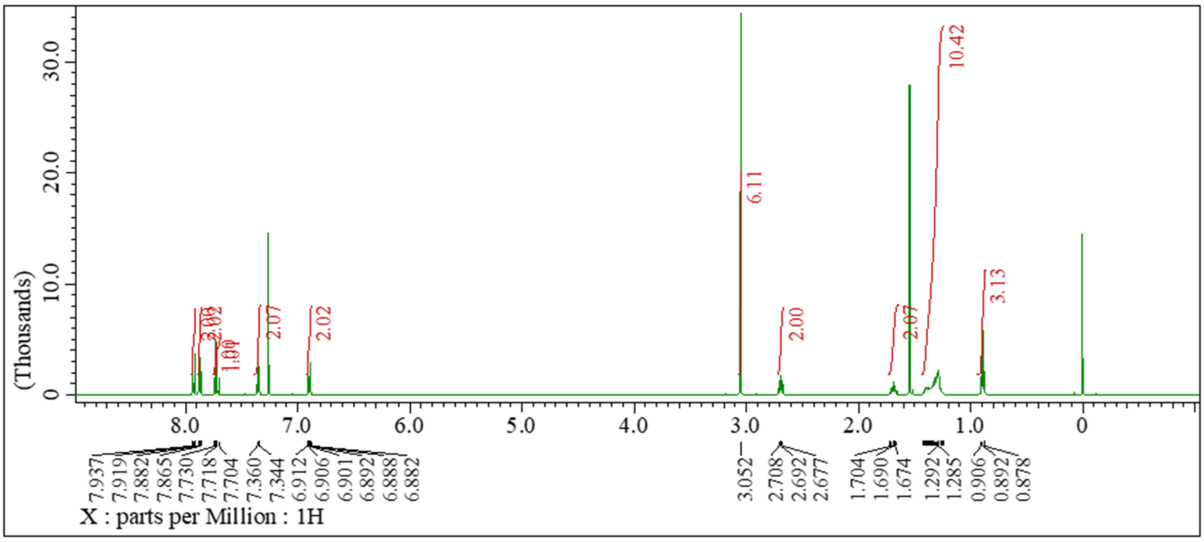

Figure S57. ^{1}H-NMR spectrum of **Me$_2$NC8** (500 MHz, $CDCl_3$).

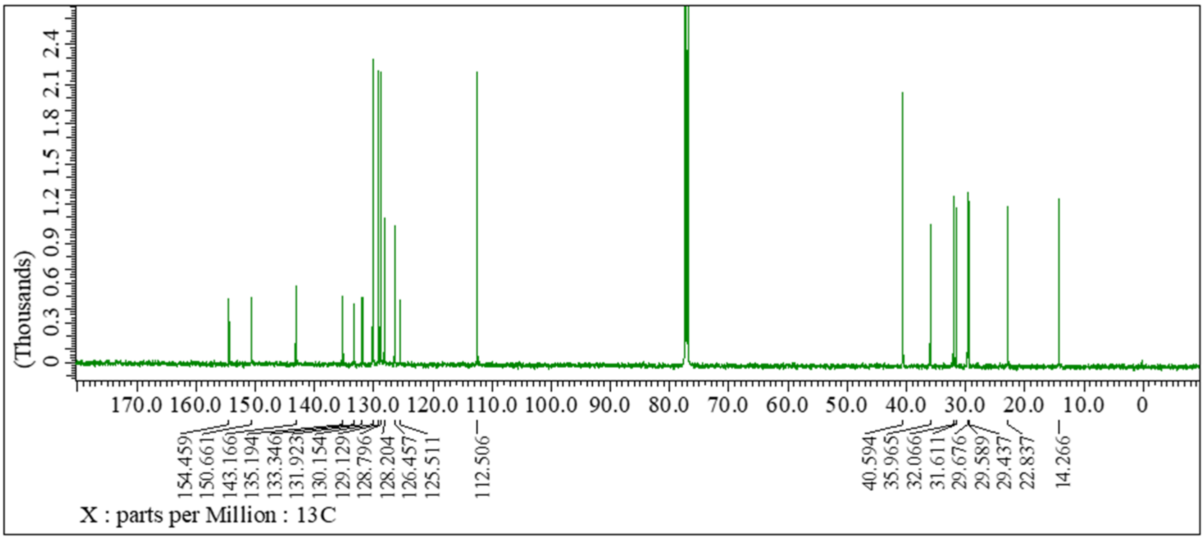

Figure S58. ^{13}C-NMR spectrum of **Me$_2$NC8** (126 MHz, $CDCl_3$).

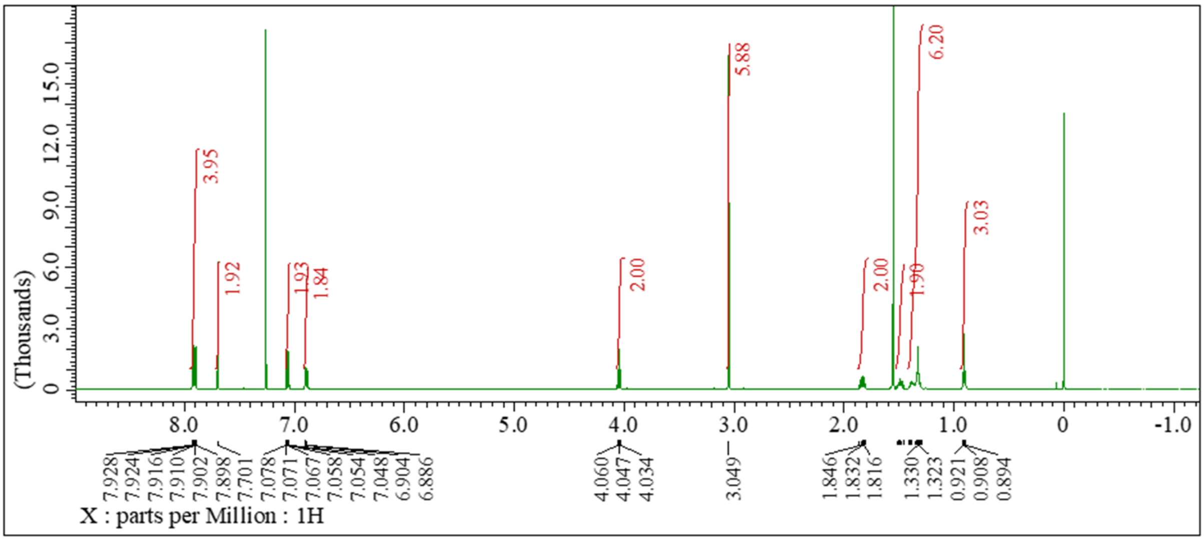

Figure S59. ^{1}H-NMR spectrum of **Me$_2$NOC7** (500 MHz, $CDCl_3$).

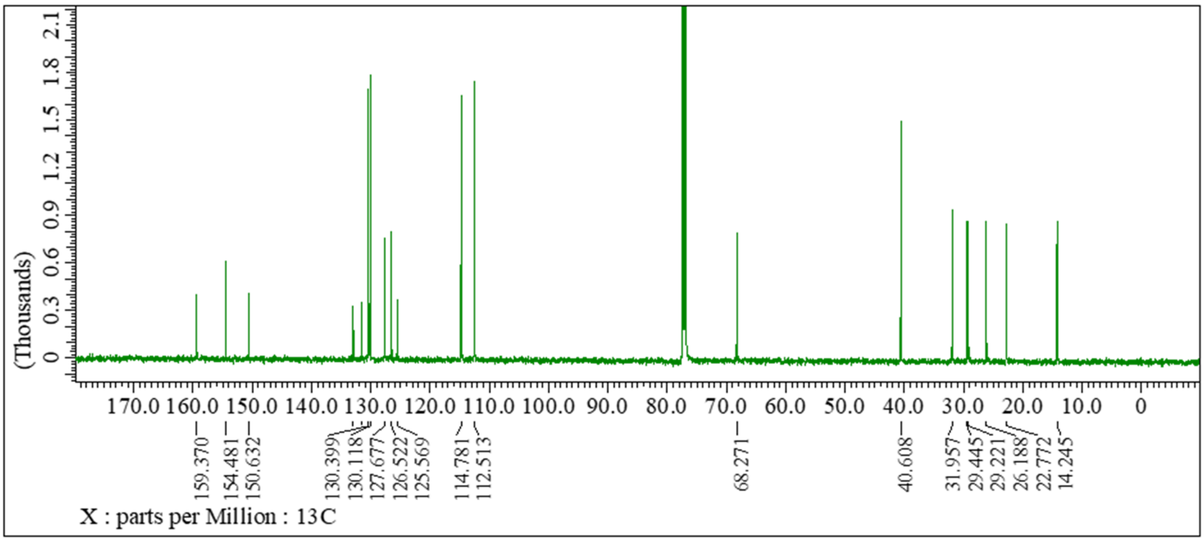

Figure S60. ^{13}C-NMR spectrum of **Me$_2$NOC7** (126 MHz, $CDCl_3$).

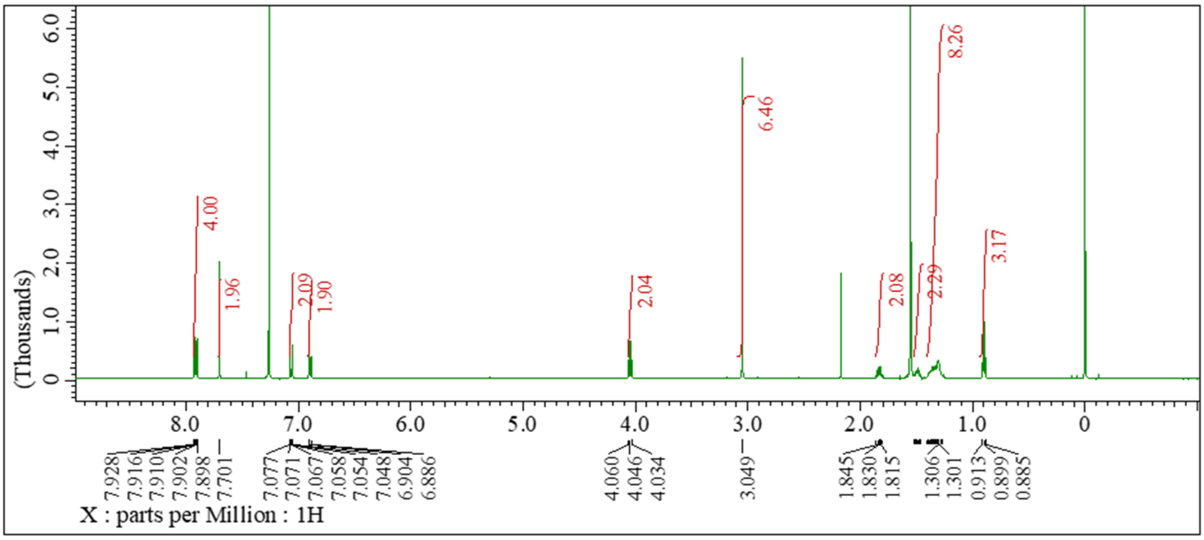

Figure S61. ^{1}H-NMR spectrum of **Me$_2$NOC8** (500 MHz, $CDCl_3$).

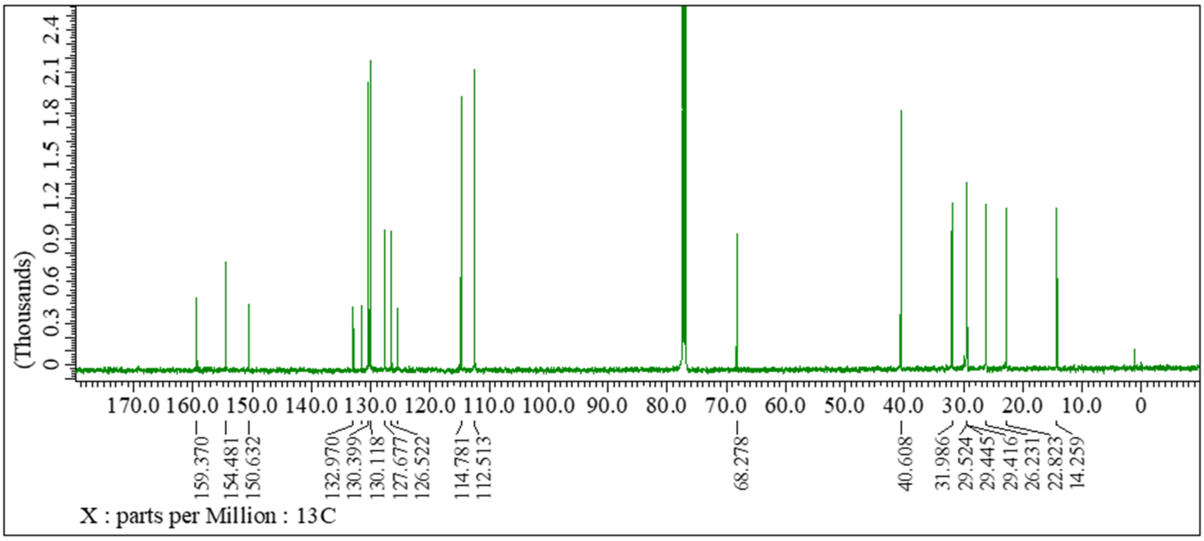

Figure S62. ^{13}C-NMR spectrum of **Me$_2$NOC8** (126 MHz, $CDCl_3$).

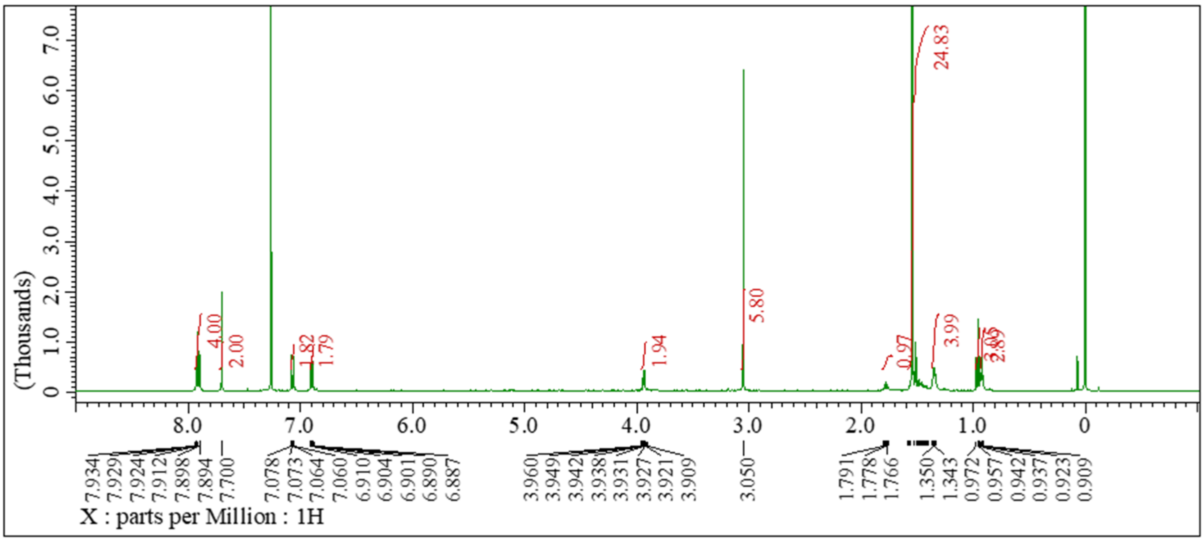

Figure S63. ^{1}H-NMR spectrum of **Me$_2$NOEH** (500 MHz, $CDCl_3$).

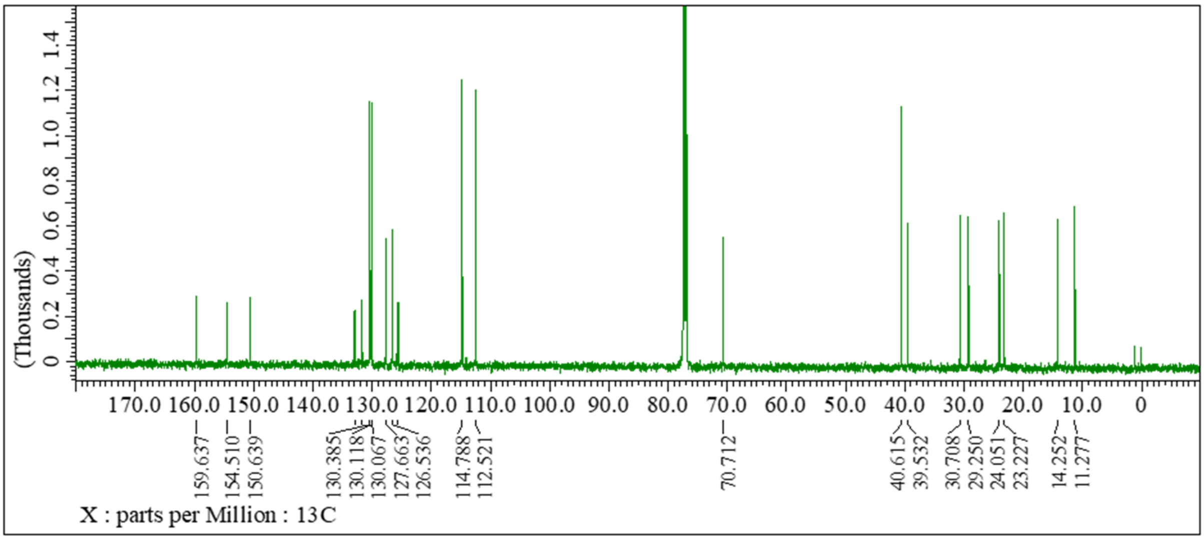

Figure S64. ^{13}C-NMR spectrum of **Me$_2$NOEH** (126 MHz, $CDCl_3$).

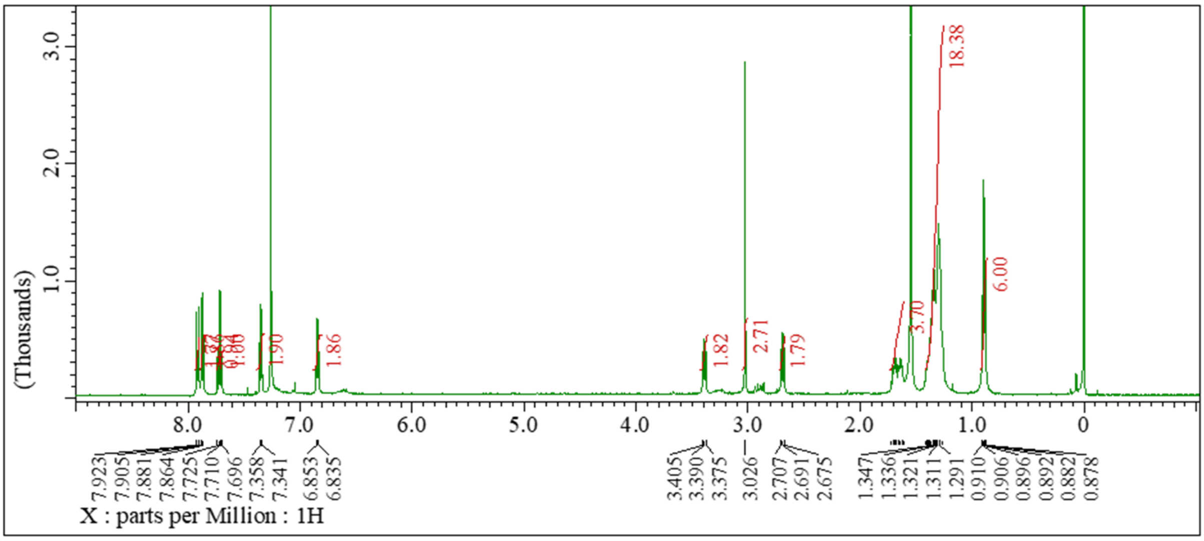

Figure S65. ^{1}H-NMR spectrum of **C8MeNC7** (500 MHz, $CDCl_3$).

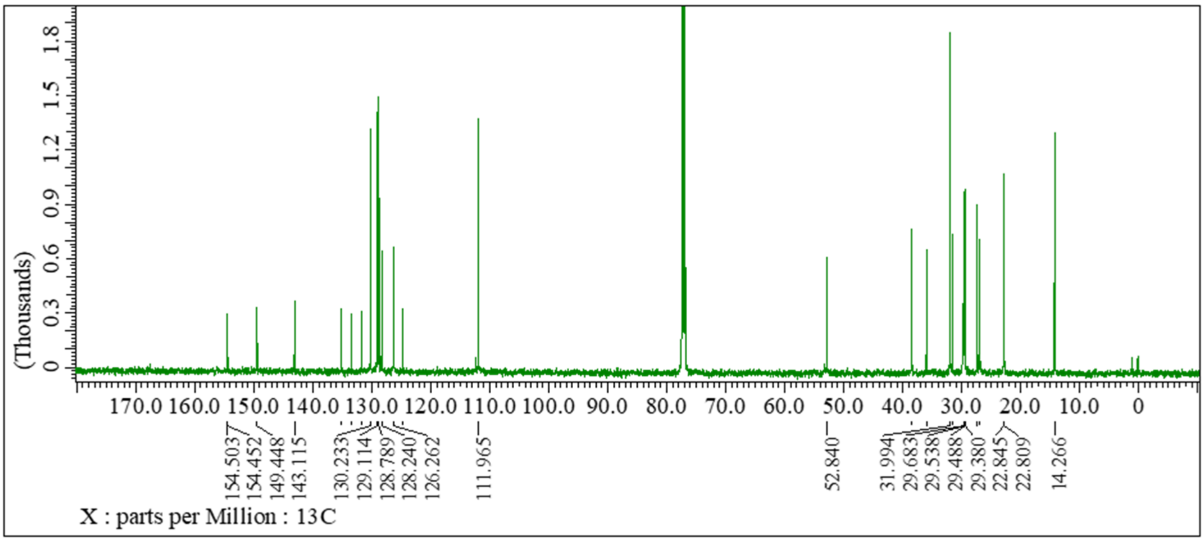

Figure S66. ^{13}C-NMR spectrum of **C8MeNC7** (126 MHz, $CDCl_3$).

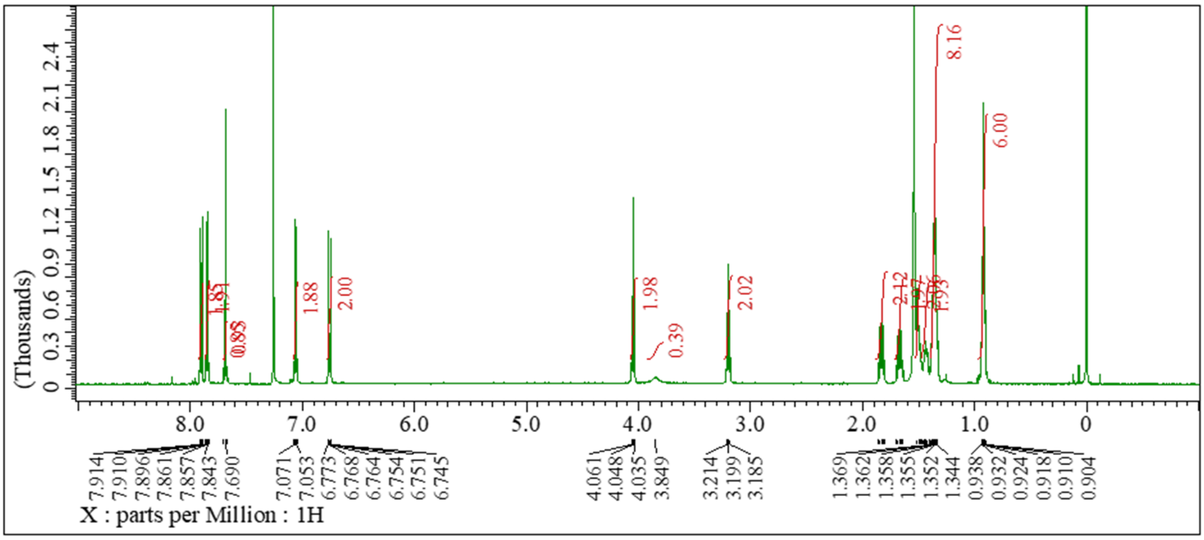

Figure S67. ^{1}H-NMR spectrum of **C6NOC6** (500 MHz, $CDCl_3$).

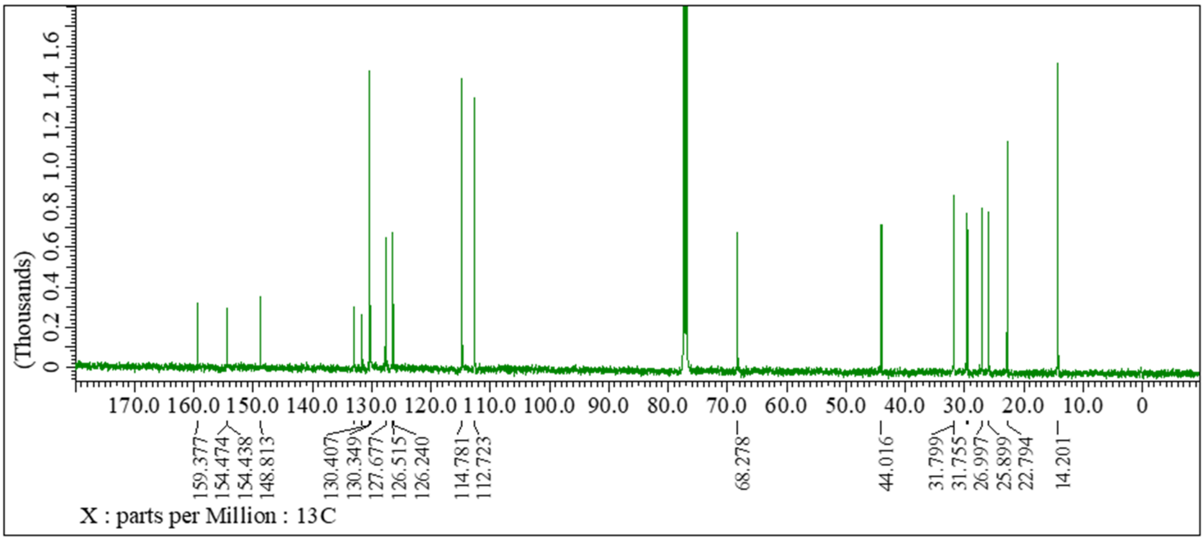

Figure S68. ^{13}C-NMR spectrum of **C6NOC6** (126 MHz, $CDCl_3$).

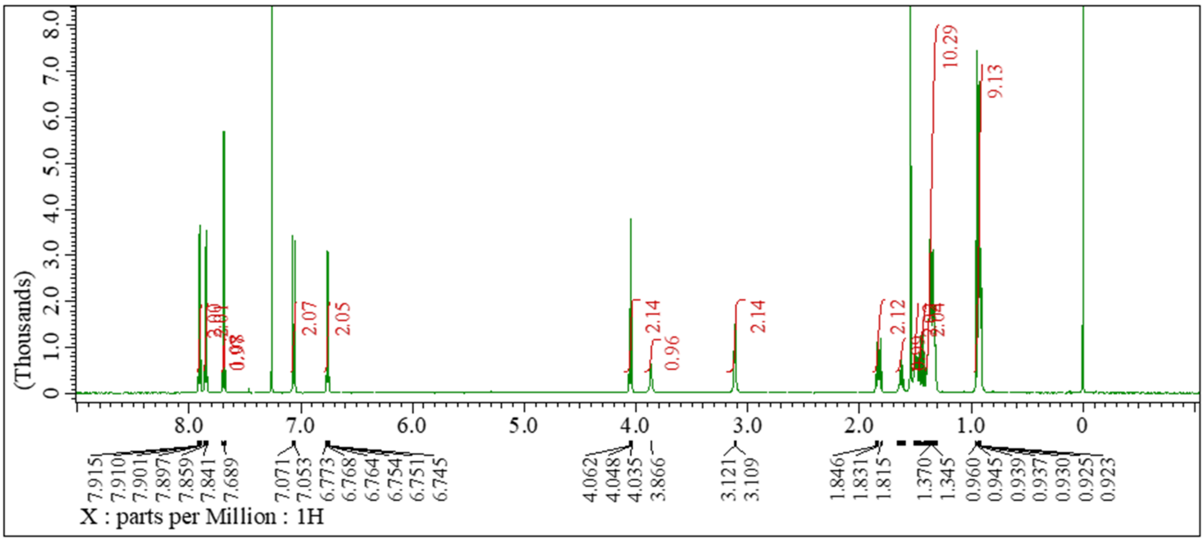

Figure S69. ^{1}H-NMR spectrum of **HENOC6** (500 MHz, $CDCl_3$).

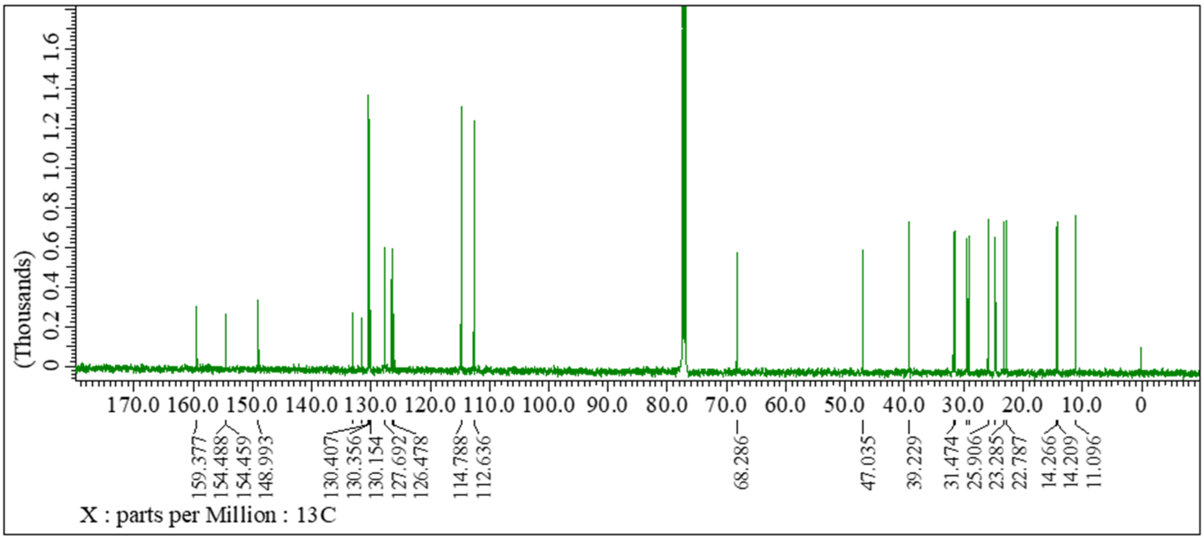

Figure S70. ^{13}C-NMR spectrum of **HENOC6** (126 MHz, $CDCl_3$).

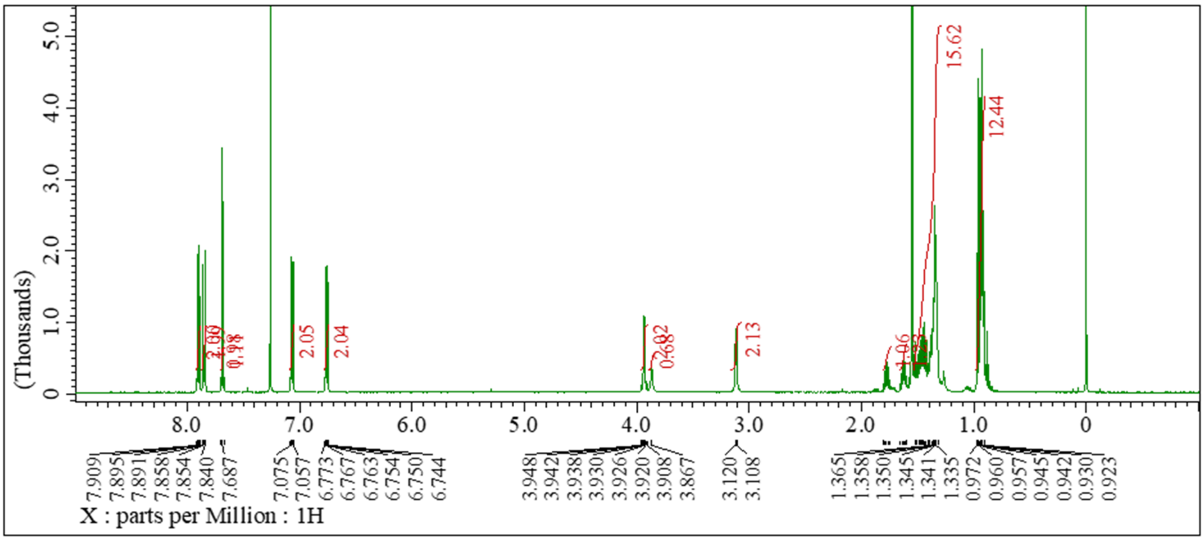

Figure S71. ^{1}H-NMR spectrum of **HENOEH** (500 MHz, $CDCl_3$).

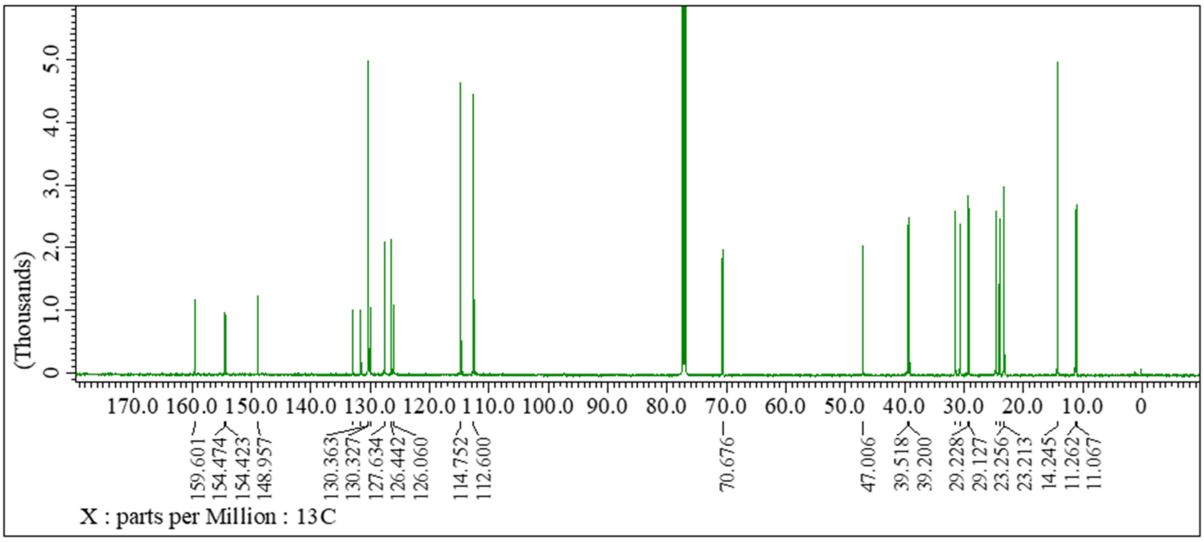

Figure S72. ^{13}C-NMR spectrum of **HENOEH** (126 MHz, $CDCl_3$).

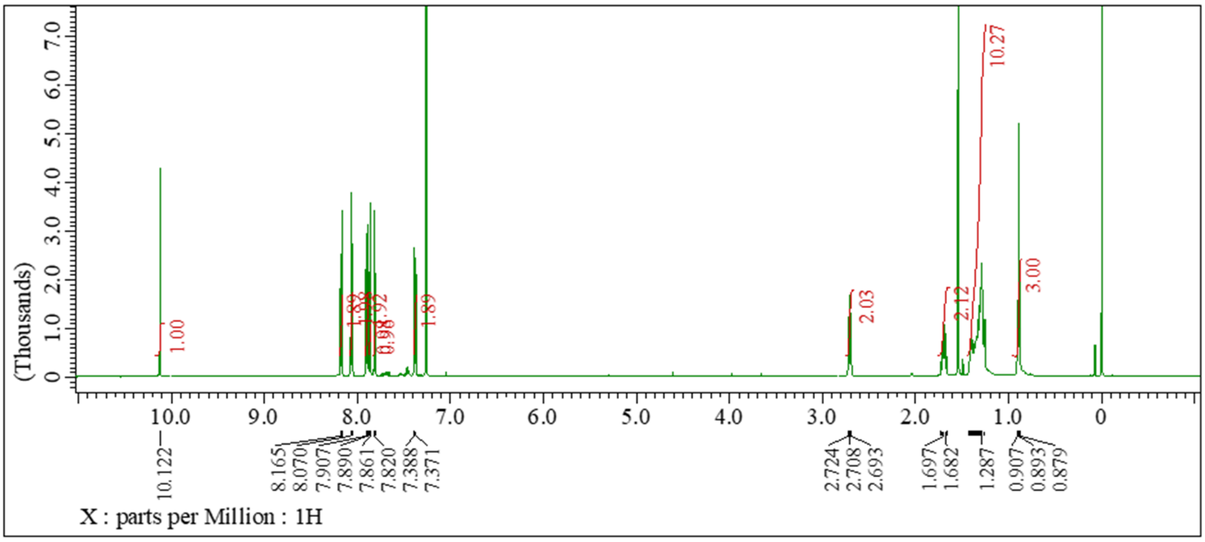

Figure S73. ^{1}H-NMR spectrum of **AldC8** (500 MHz, $CDCl_3$).

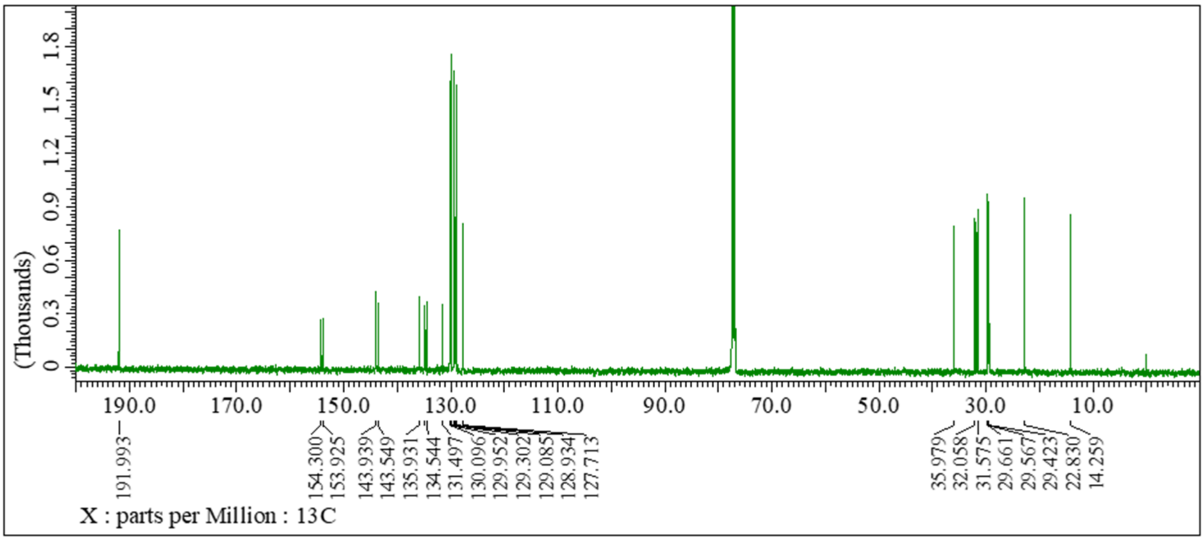

Figure S74. ^{13}C-NMR spectrum of **AldC8** (500 MHz, $CDCl_3$).

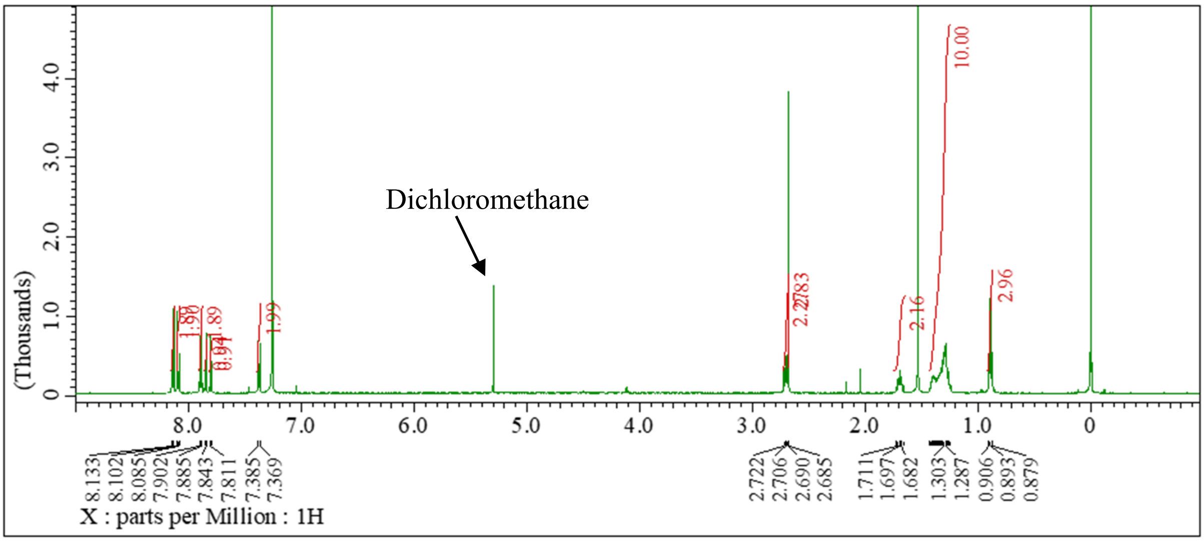

Figure S75. ^{1}H-NMR spectrum of **ActC8** (500 MHz, $CDCl_3$).

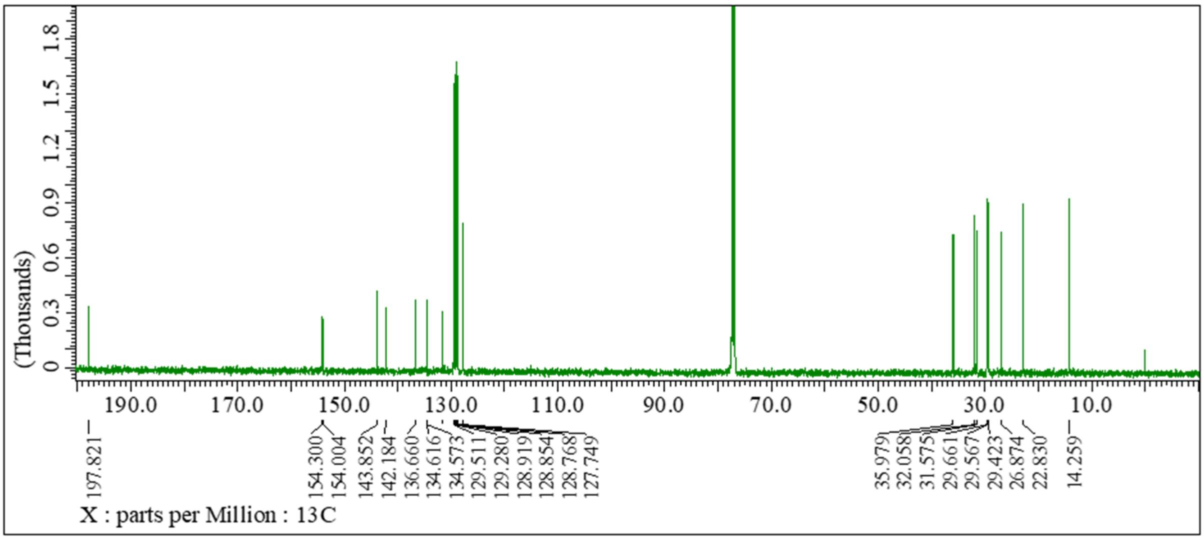

Figure S76. ^{13}C-NMR spectrum of **ActC8** (500 MHz, $CDCl_3$).

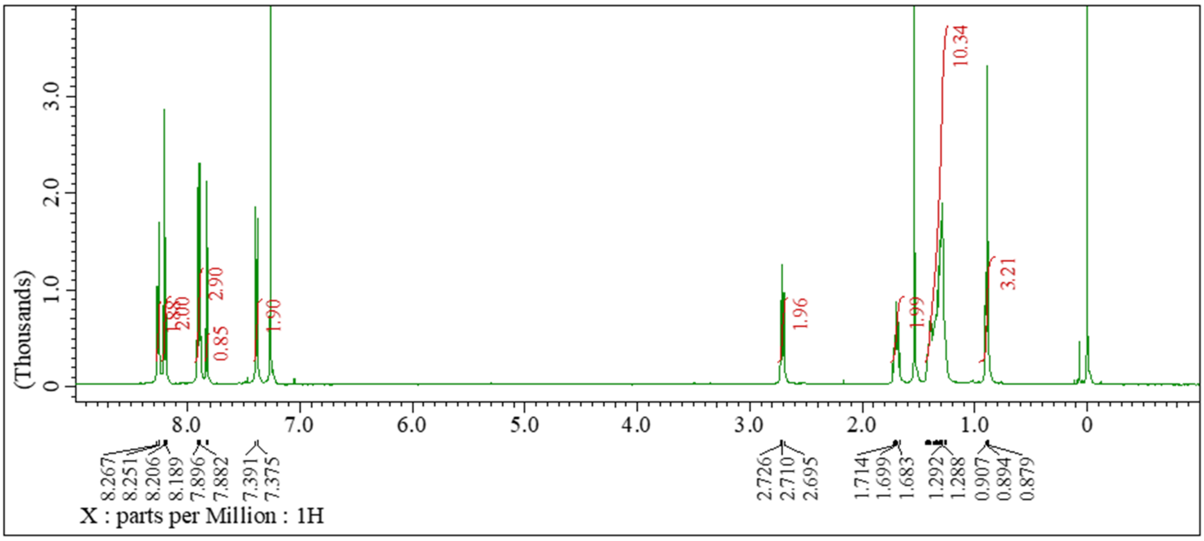

Figure S77. ^{1}H-NMR spectrum of **TFActC8** (500 MHz, $CDCl_3$).

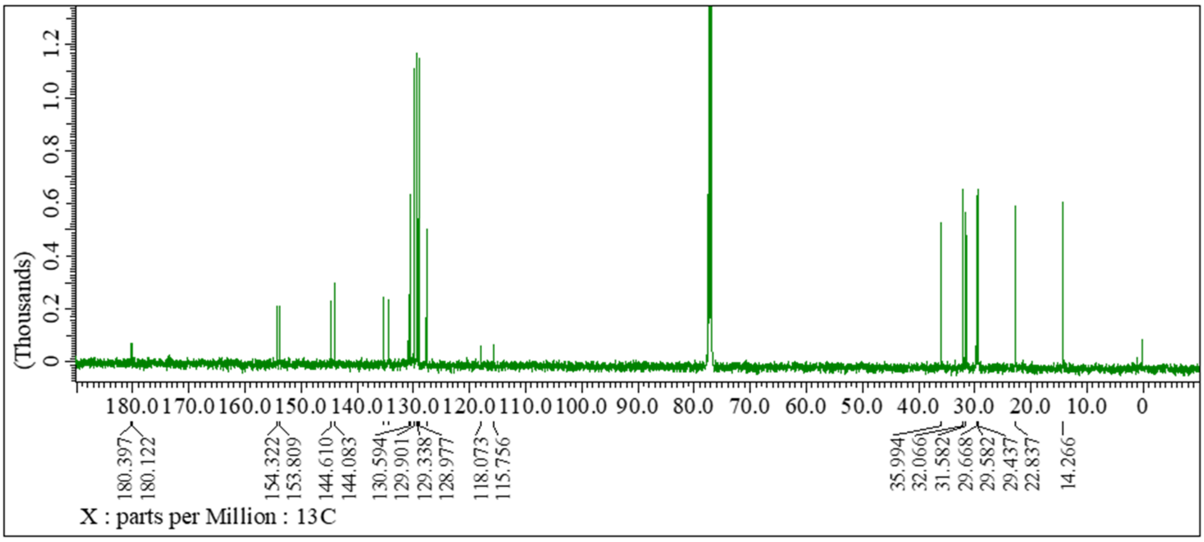

Figure S78. ^{13}C-NMR spectrum of **TFActC8** (126 MHz, $CDCl_3$).

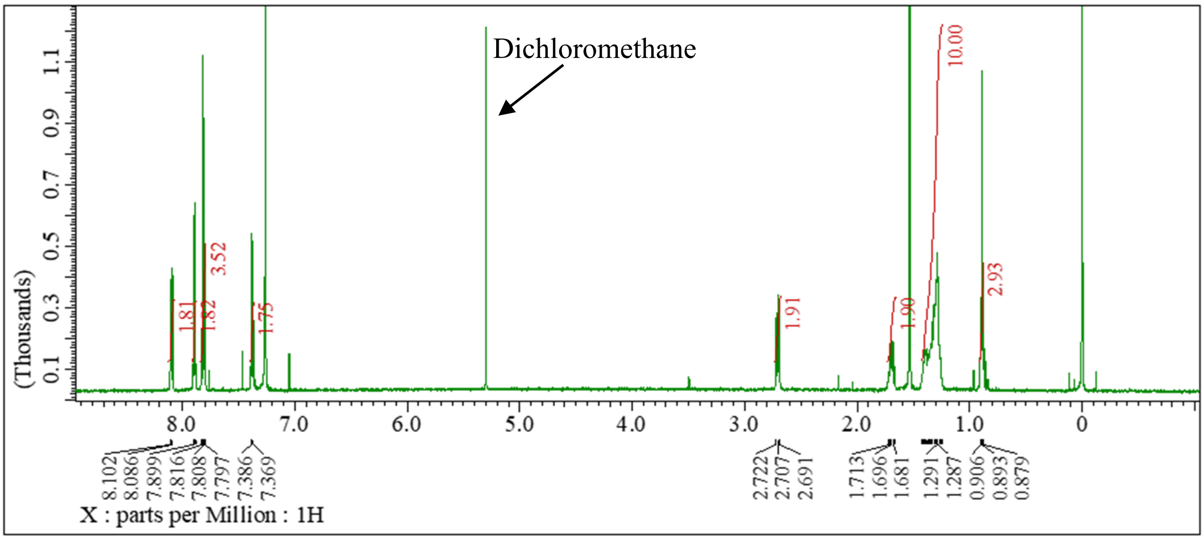

Figure S79. ^{1}H-NMR spectrum of **TFMeC8** (500 MHz, $CDCl_3$).

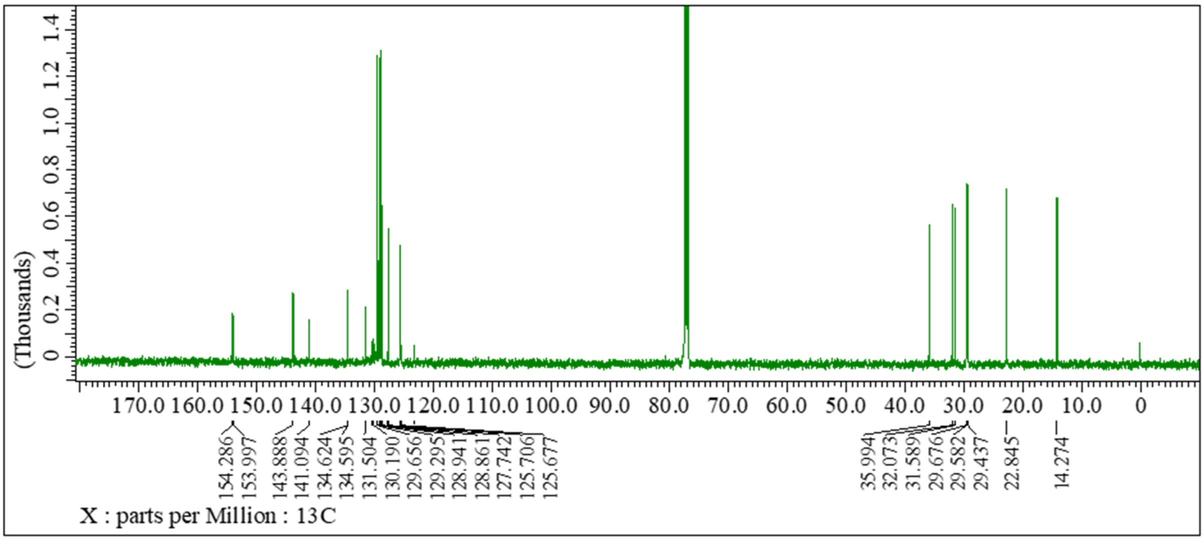

Figure S80. ^{13}C-NMR spectrum of **TFMeC8** (126 MHz, $CDCl_3$).

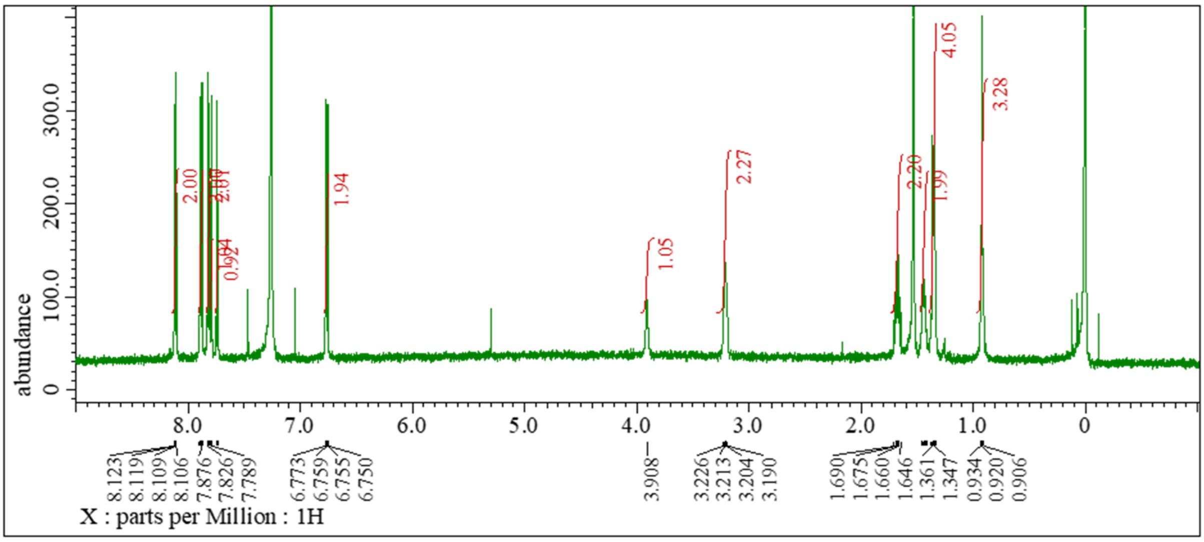

Figure S81. ^{1}H-NMR spectrum of **C6NCN** (500 MHz, $CDCl_3$).

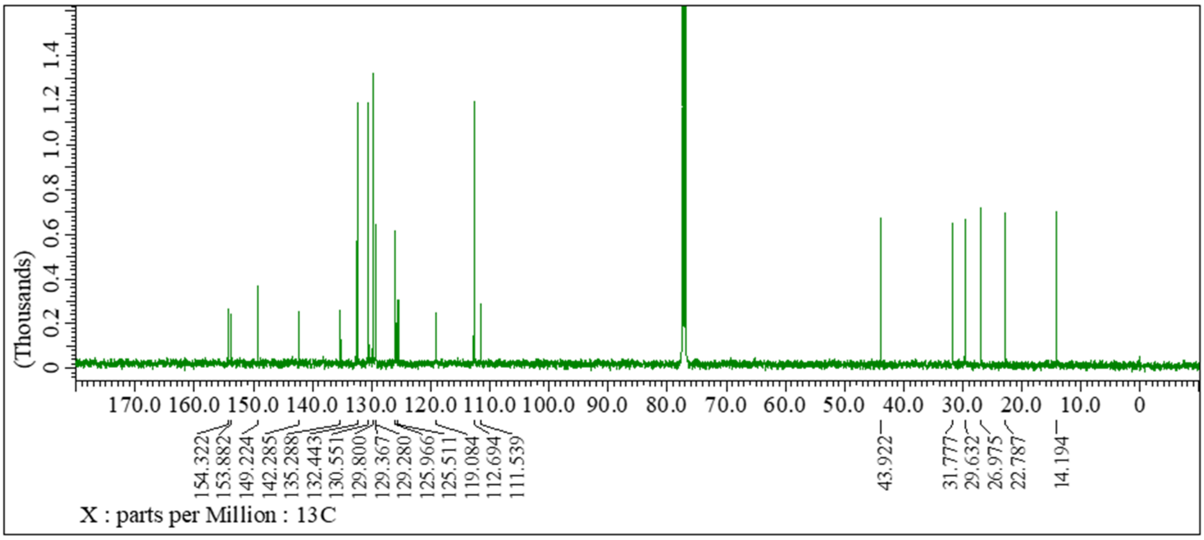

Figure S82. ^{13}C-NMR spectrum of **C6NCN** (126 MHz, $CDCl_3$).

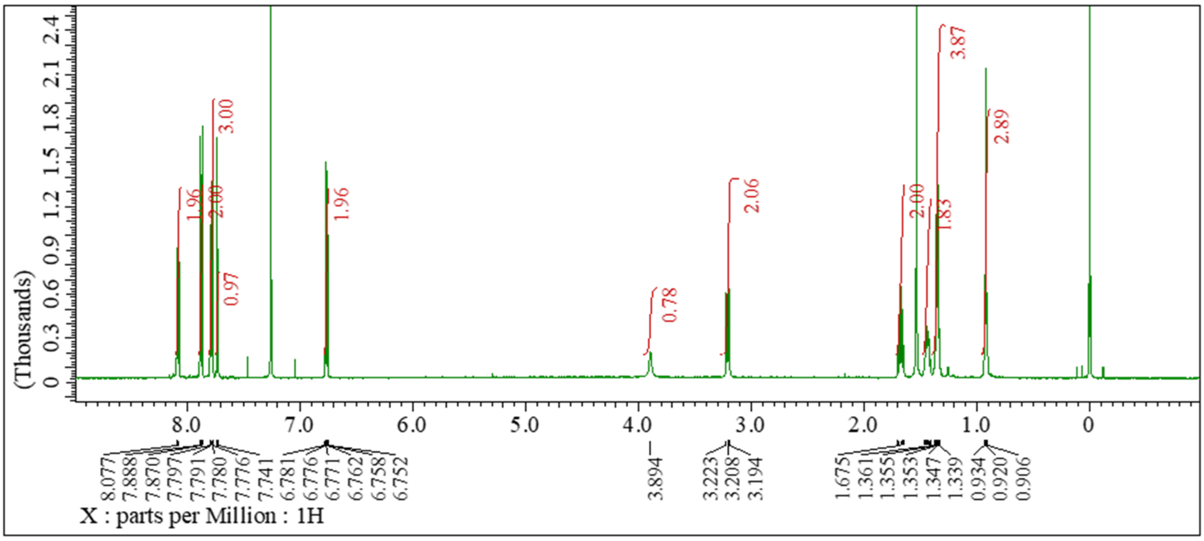

Figure S83. ^{1}H-NMR spectrum of **C6NTFMe** (500 MHz, $CDCl_3$).

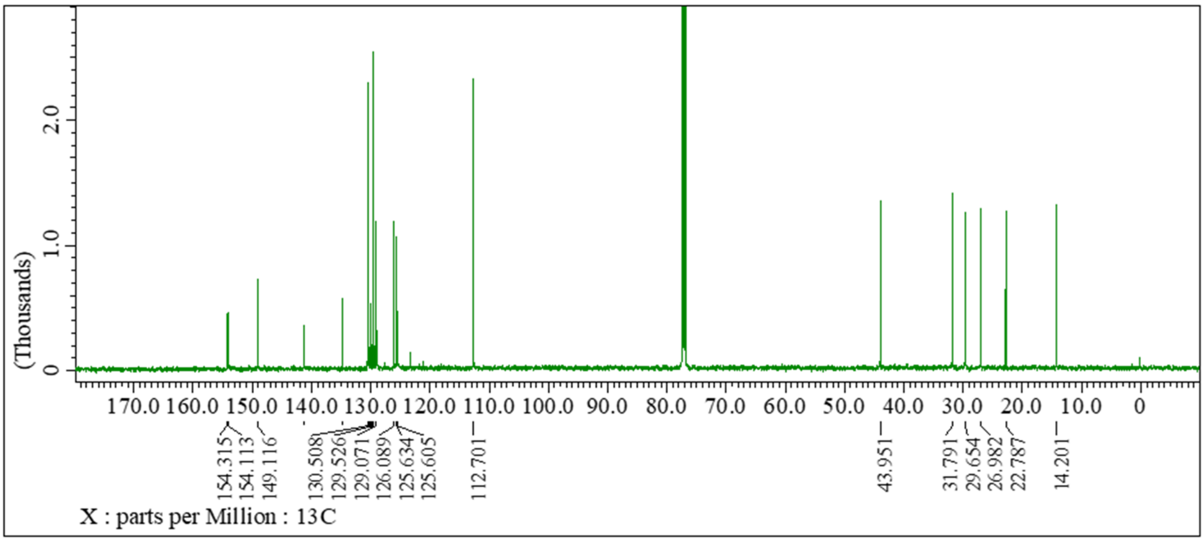

Figure S84. ^{13}C-NMR spectrum of **C6NTFMe** (126 MHz, $CDCl_3$).

FT-IR spectra

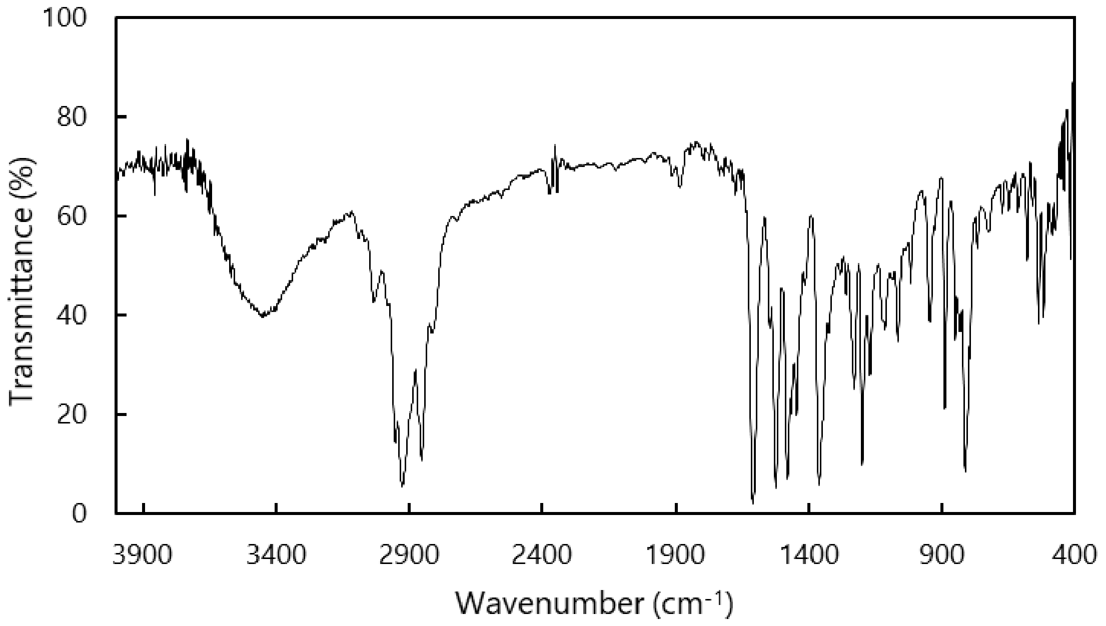

Figure S85. FT-IR spectrum of **Me$_2$NC7**

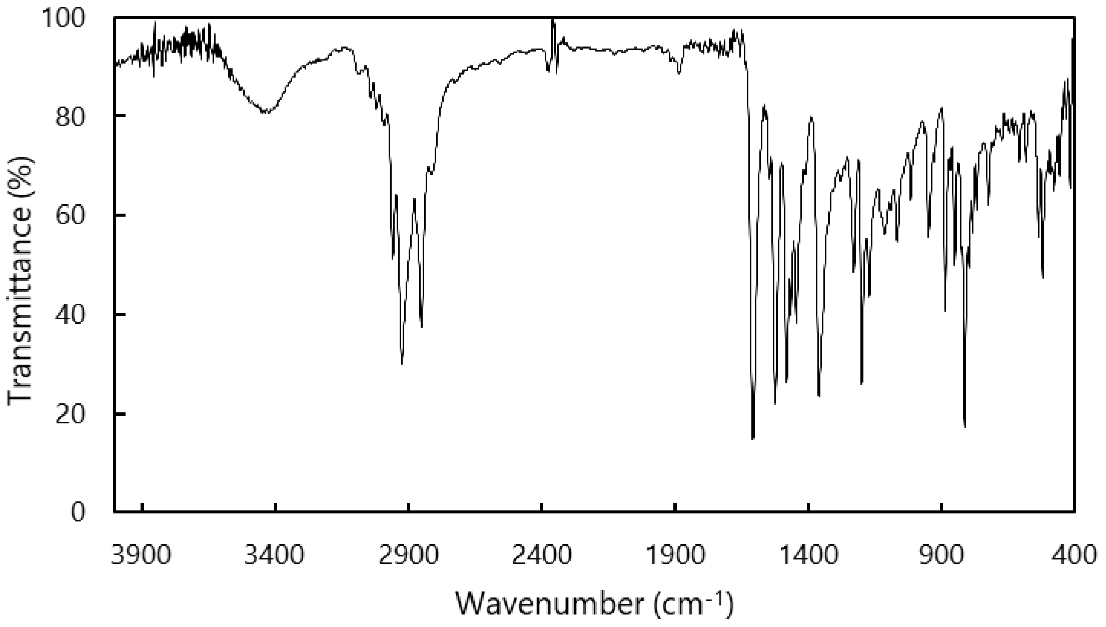

Figure S86. FT-IR spectrum of **Me$_2$NC8**

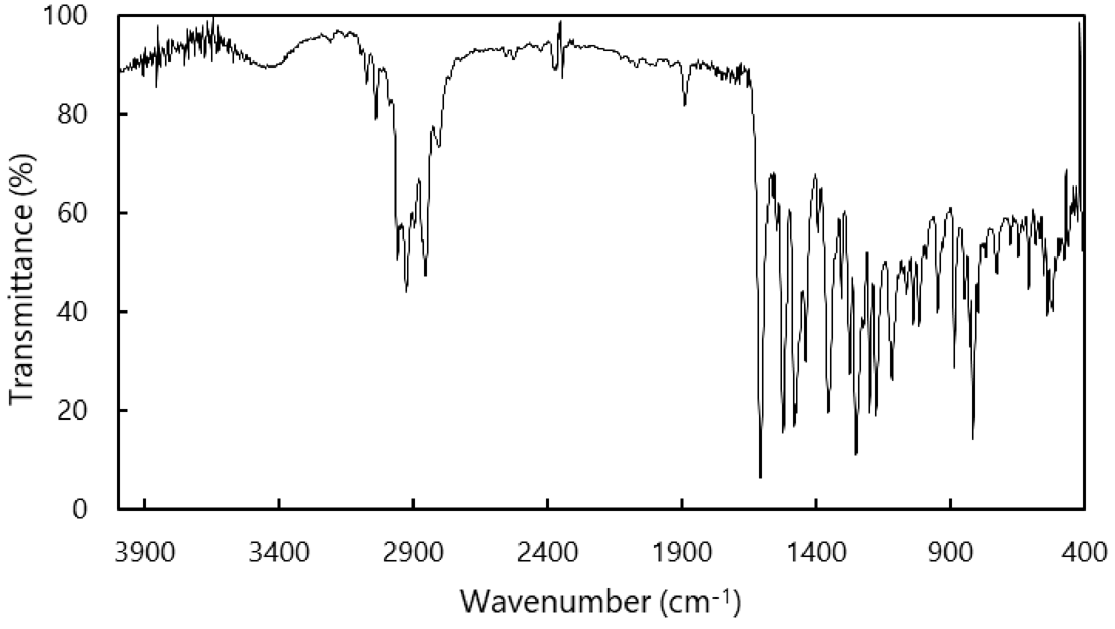

Figure S87. FT-IR spectrum of **Me_2NOC7**

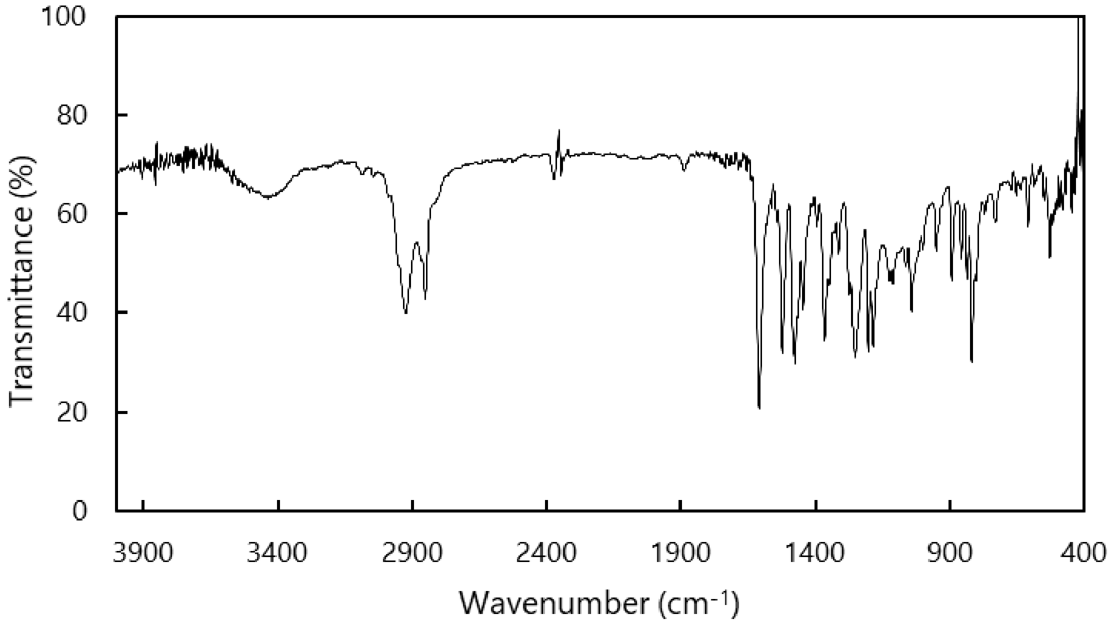

Figure S88. FT-IR spectrum of **Me_2NOC8**

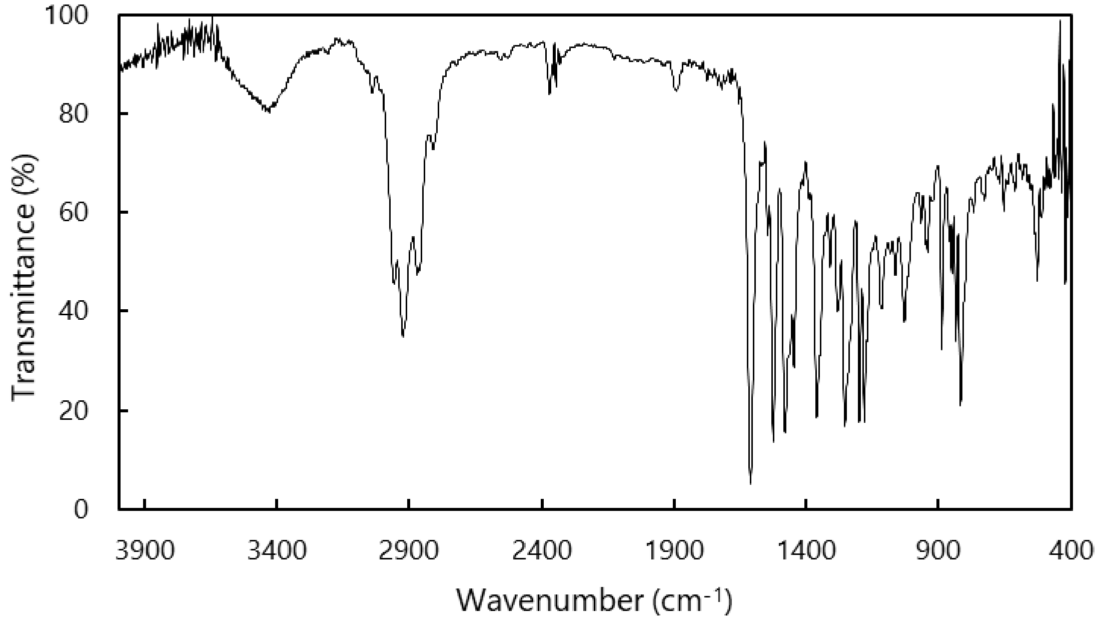

Figure S89. FT-IR spectrum of **Me$_2$NOEH**

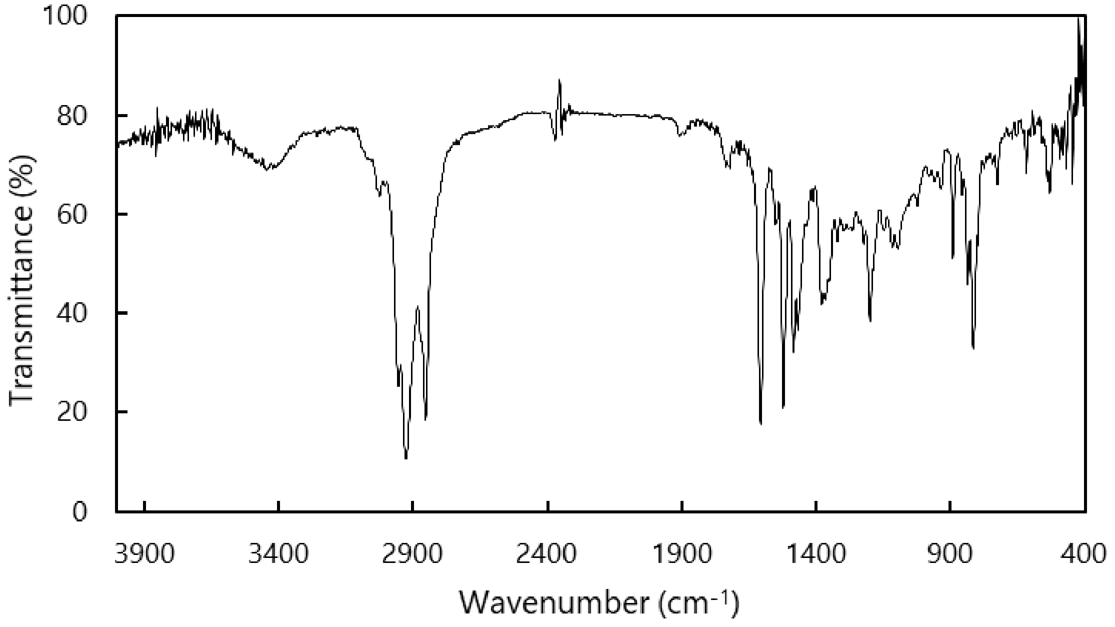

Figure S90. FT-IR spectrum of **C8MeNC7**

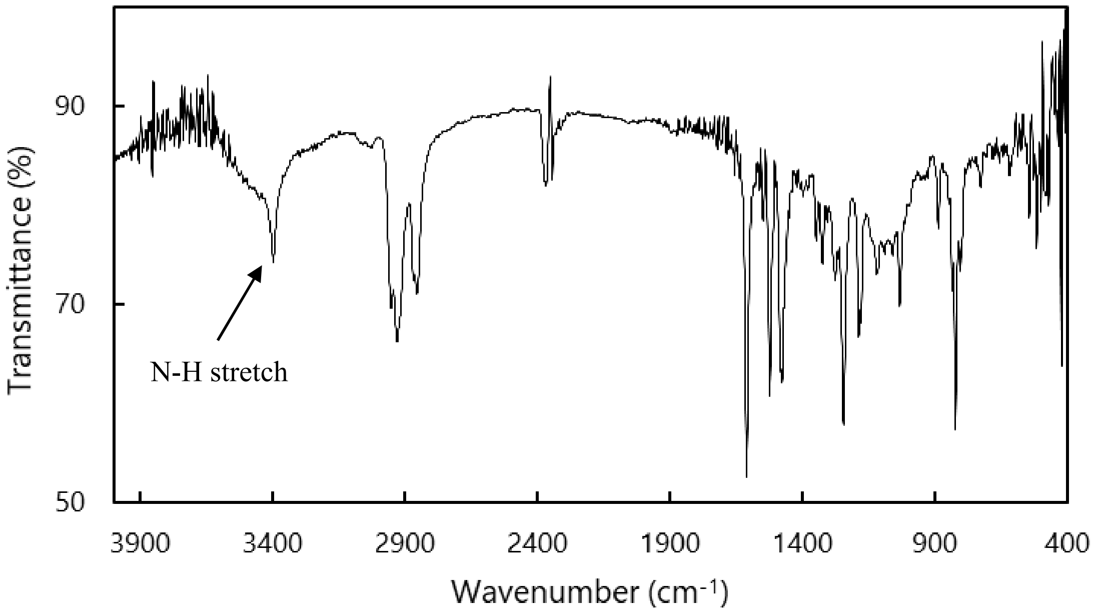

Figure S91. FT-IR spectrum of **C6NOC6**

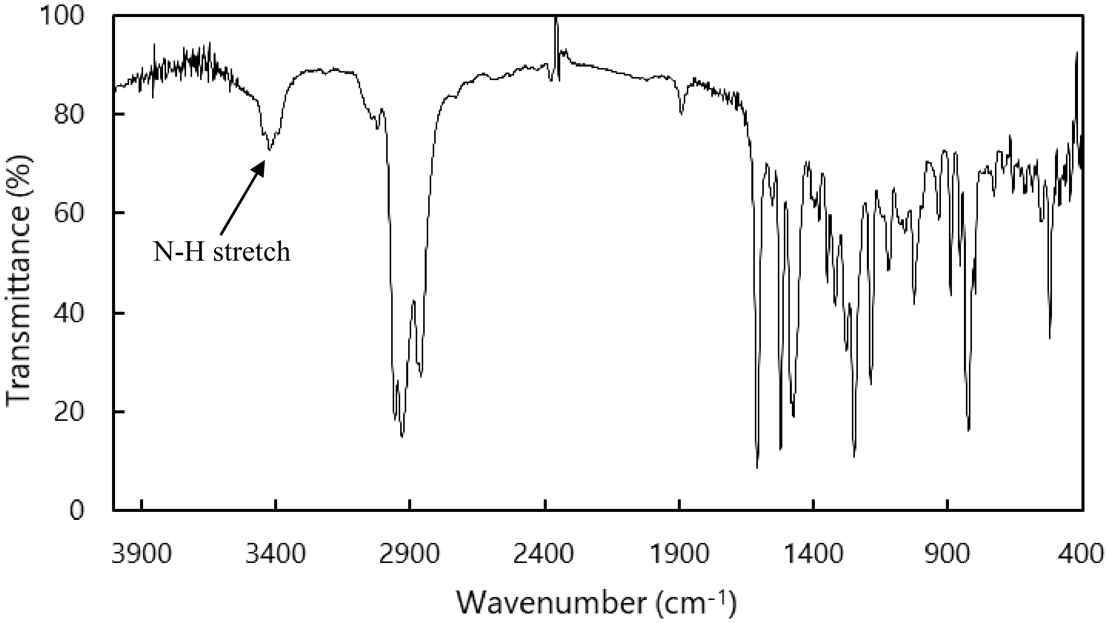

Figure S92. FT-IR spectrum of **EHNOC6**

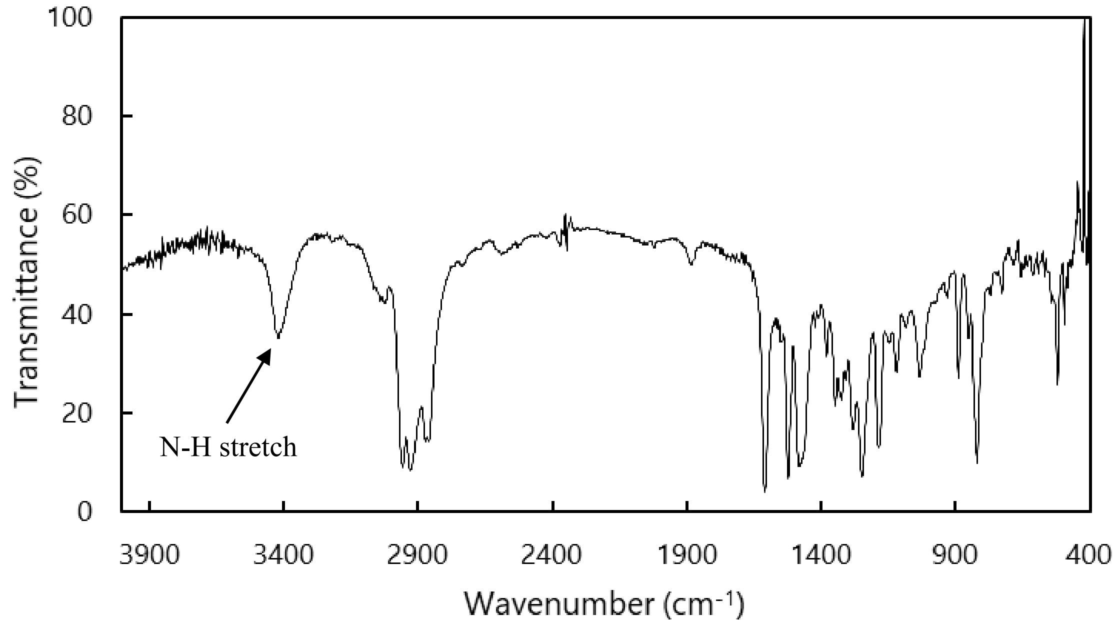

Figure S93. FT-IR spectrum of **EHNOEH**

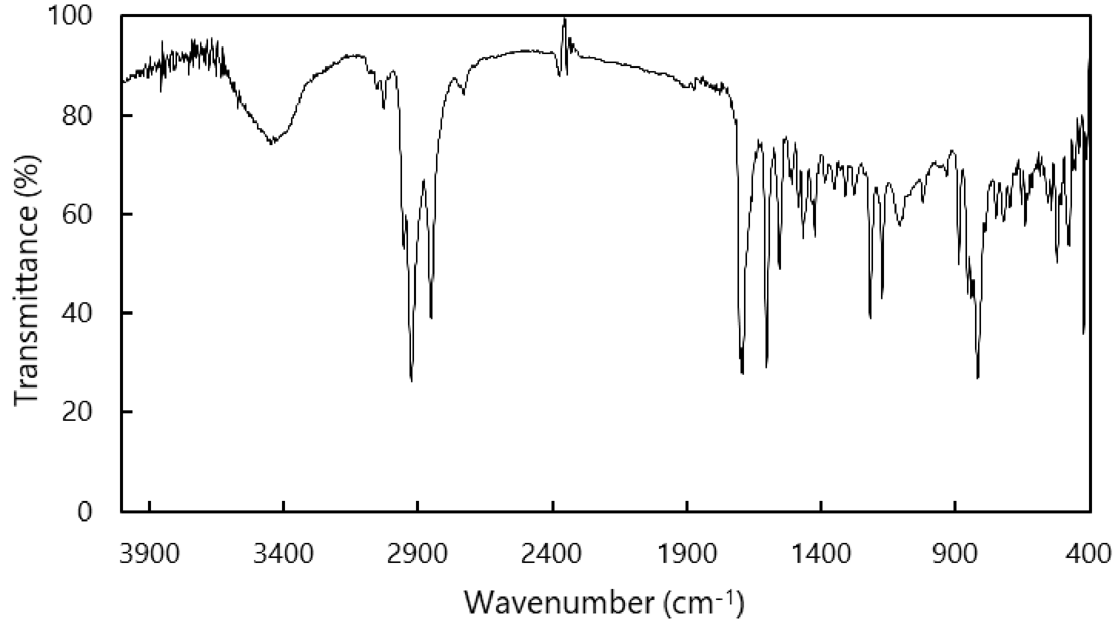

Figure S94. FT-IR spectrum of **AldC8**

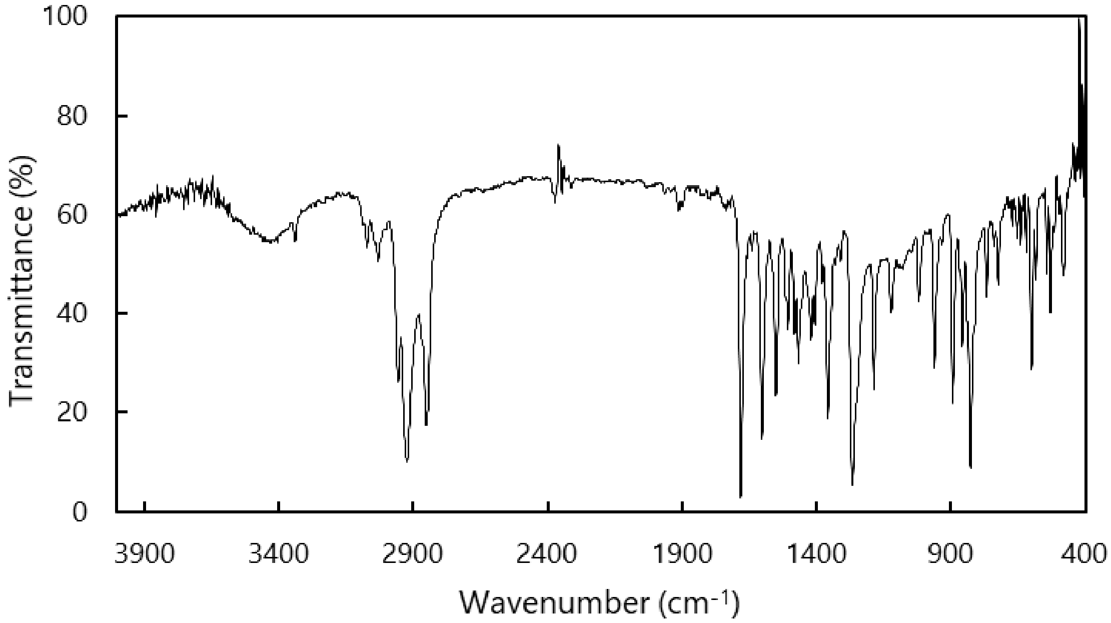

Figure S95. FT-IR spectrum of **ActC8**

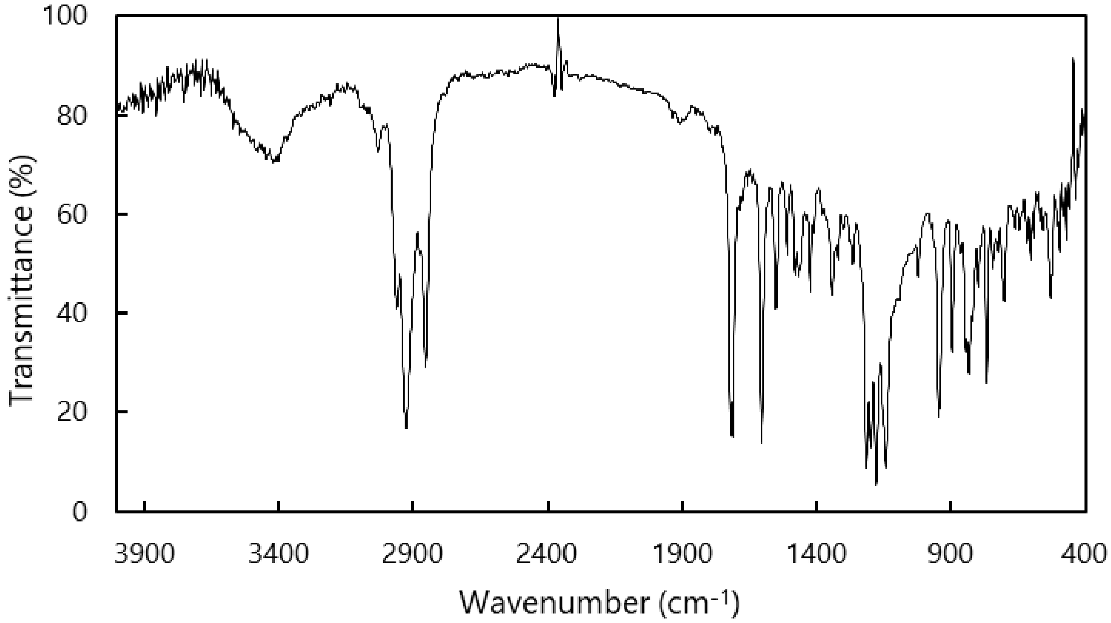

Figure S96. FT-IR spectrum of **TFActC8**

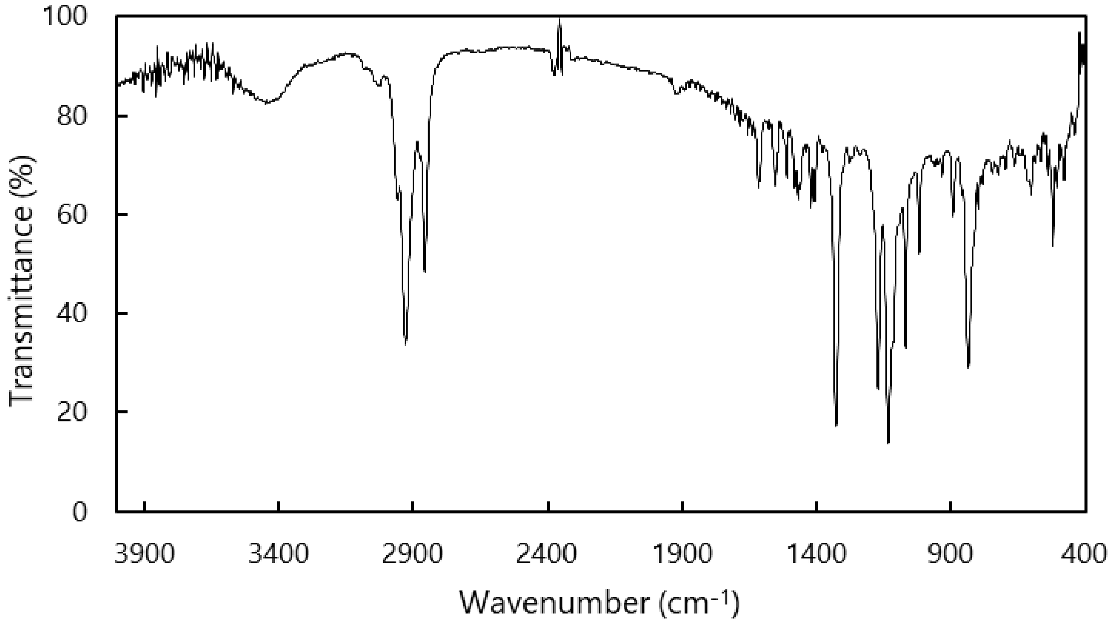

Figure S97. FT-IR spectrum of **TFMeC8**

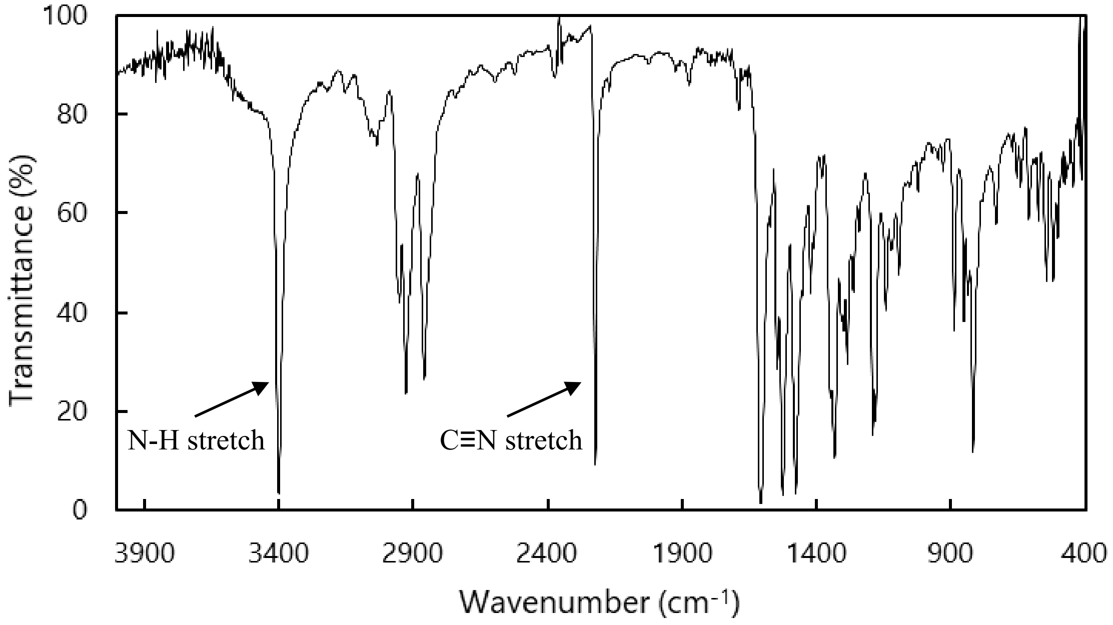

Figure S98. FT-IR spectrum of **C6NCN**

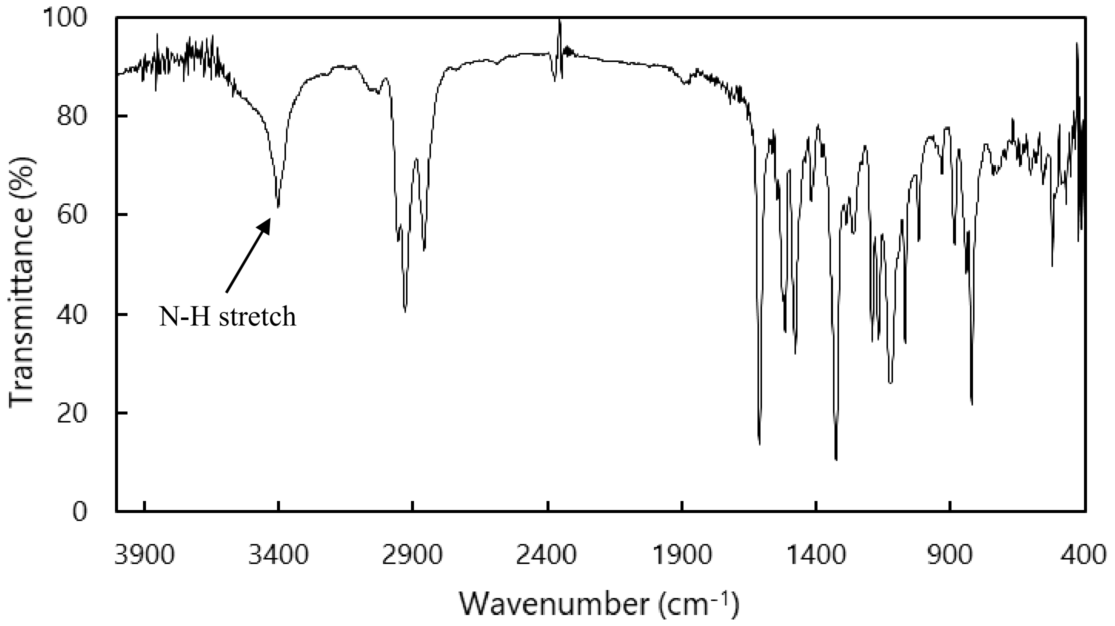

Figure S99. FT-IR spectrum of **C6NTFMe**

High-resolution mass spectrometry (HRMS) chart

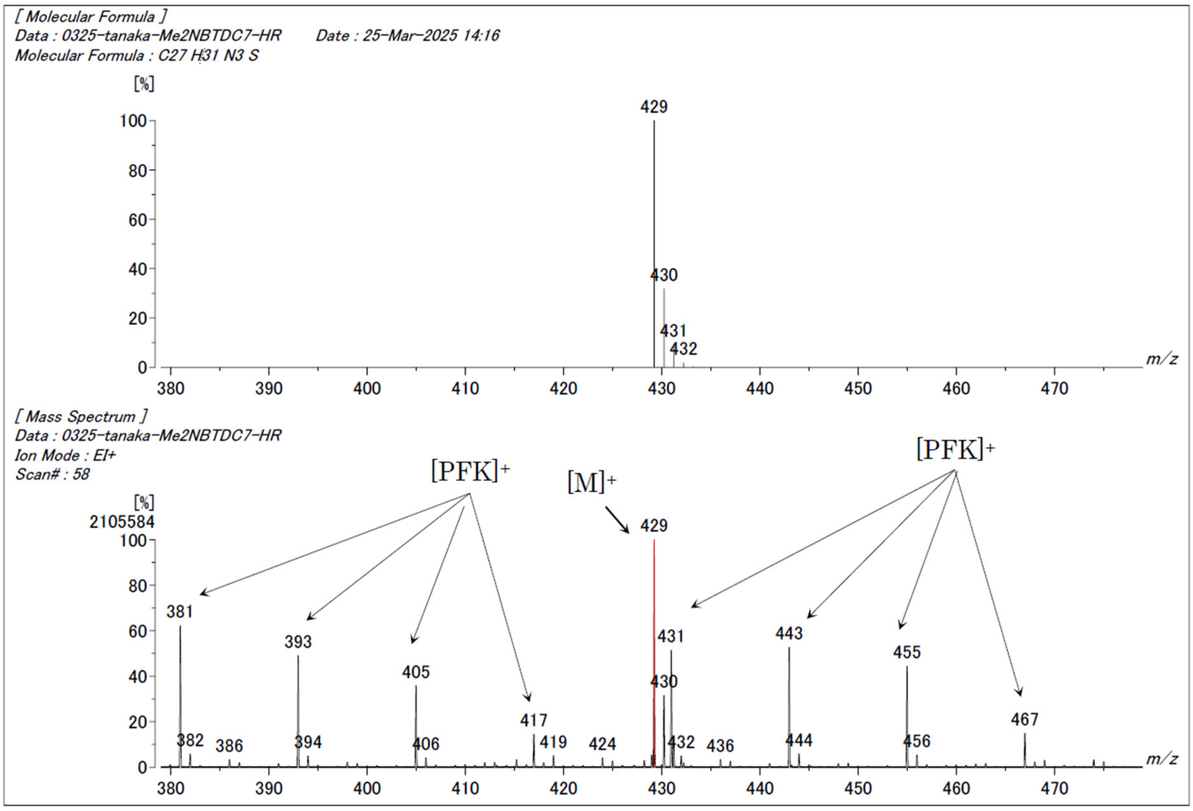

Figure S100. HR-MS spectra of **Me$_2$NC7** (above: calculated data; below: experimental data).

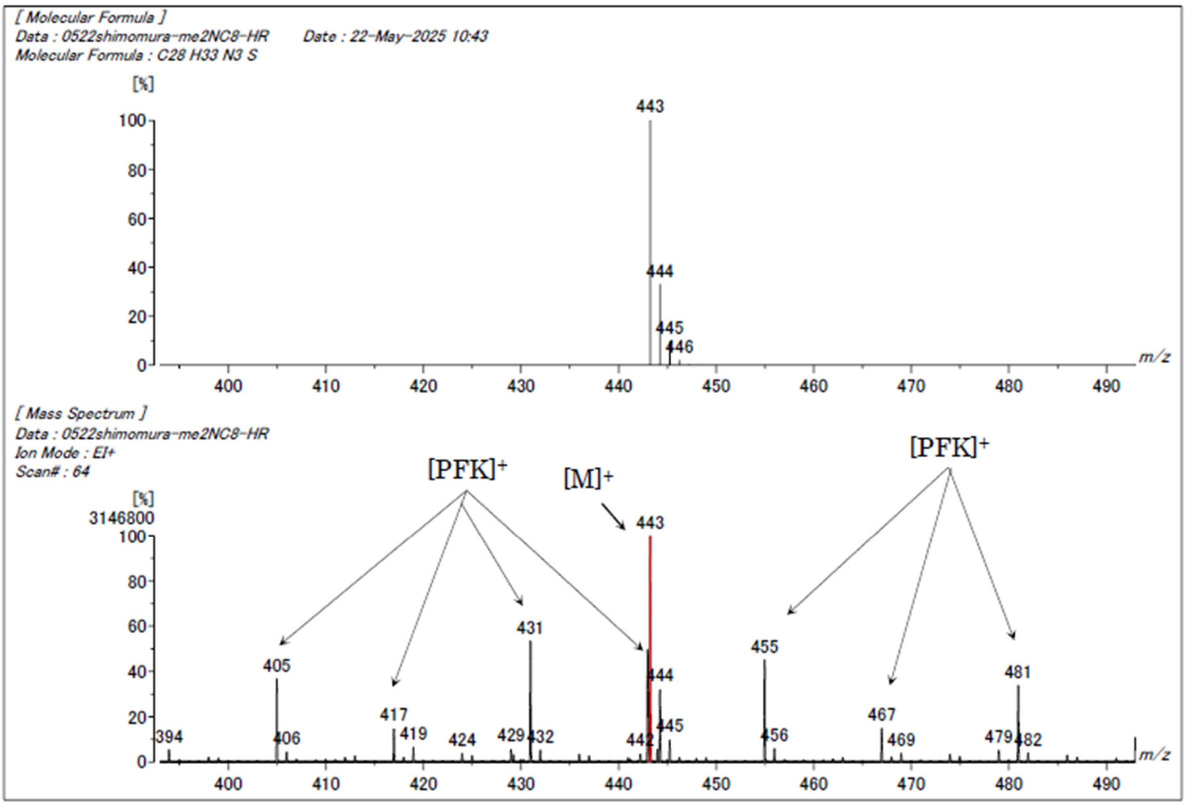

Figure S101. HR-MS spectra of **Me$_2$NC8** (above: calculated data; below: experimental data).

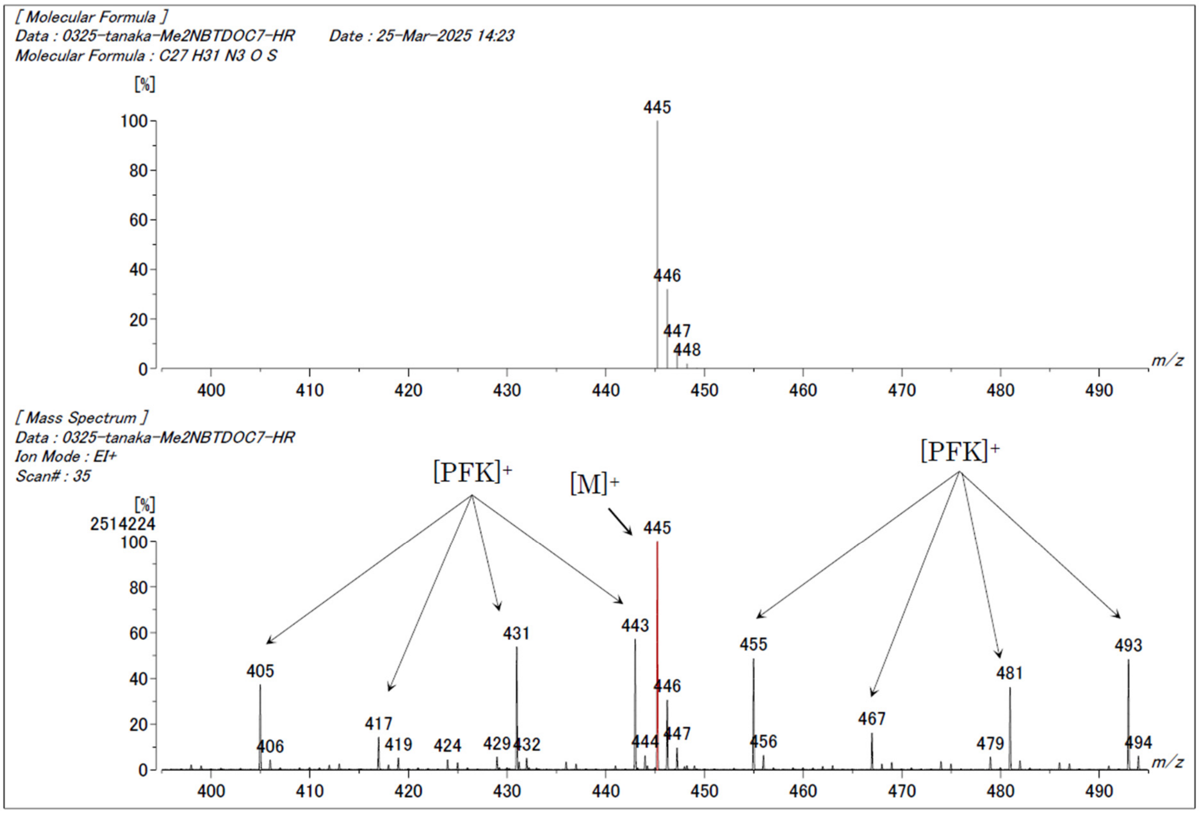

Figure S102. HR-MS spectra of **Me_2NOC7** (above: calculated data; below: experimental data).

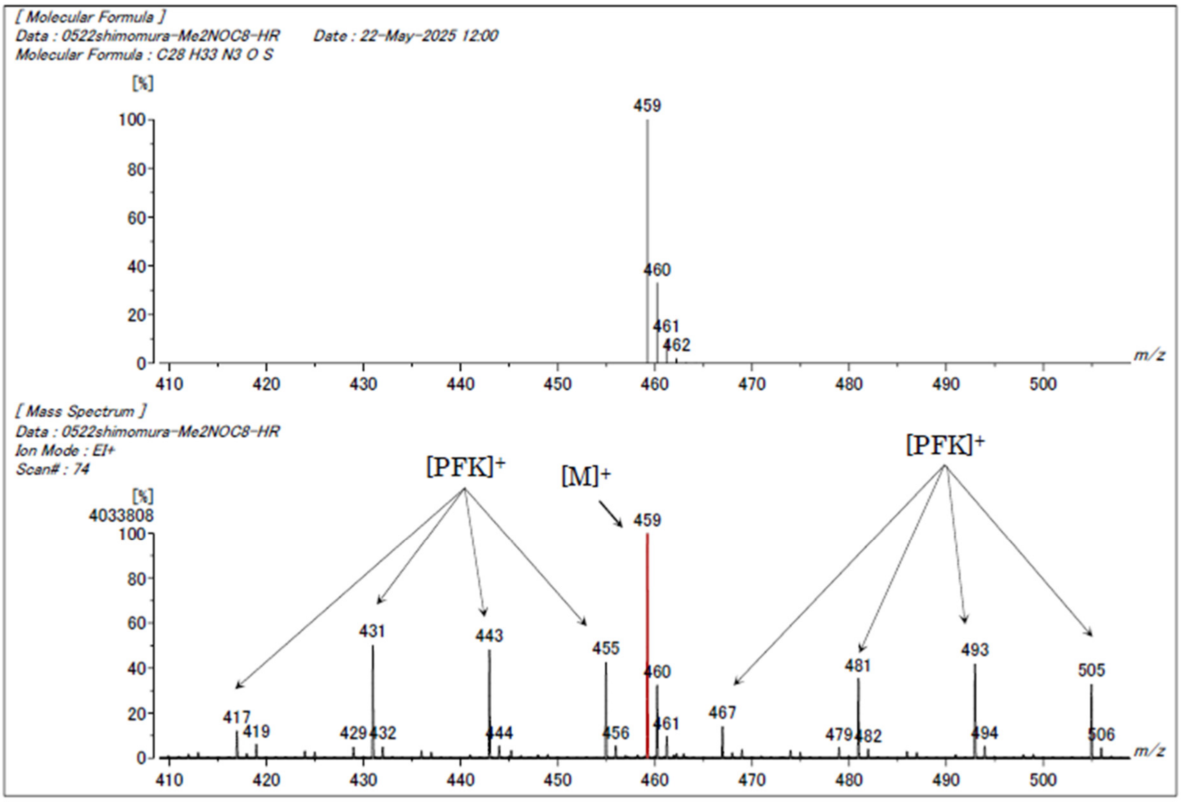

Figure S103. HR-MS spectra of **Me_2NOC8** (above: calculated data; below: experimental data).

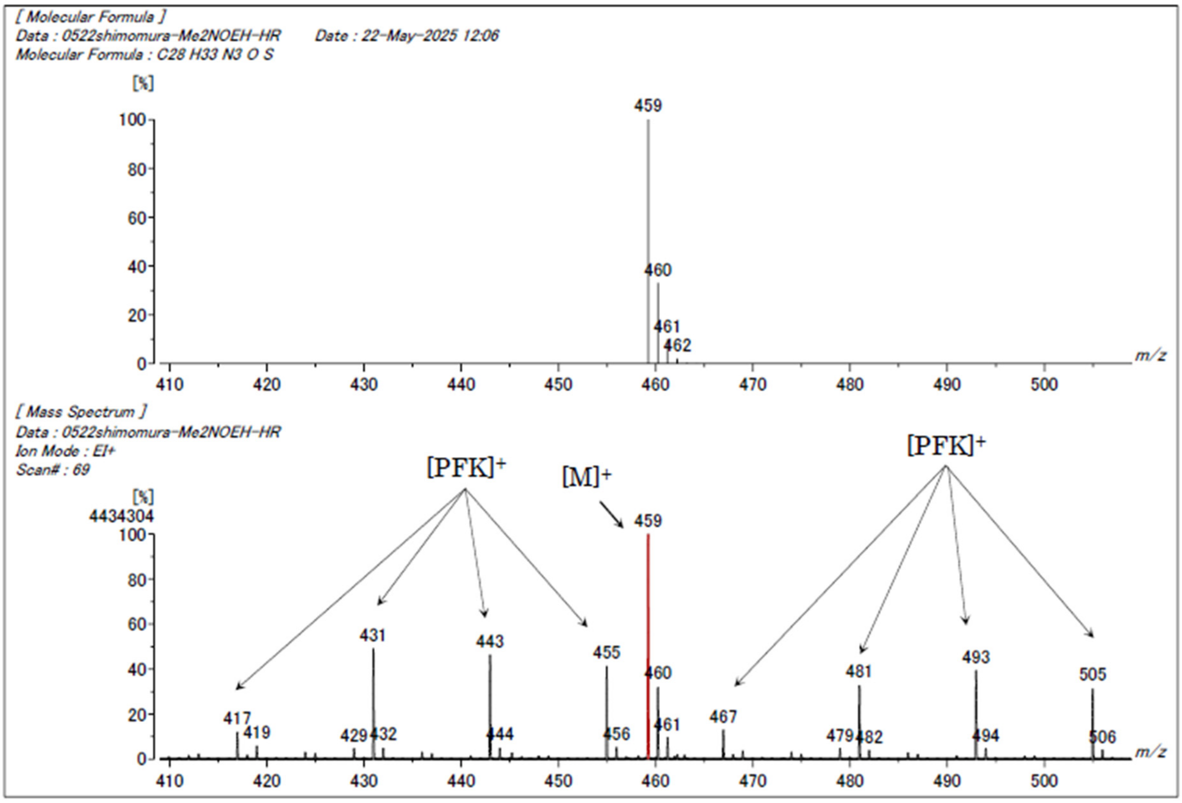

Figure S104. HR-MS spectra of **Me_2NOCEH** (above: calculated data; below: experimental data).

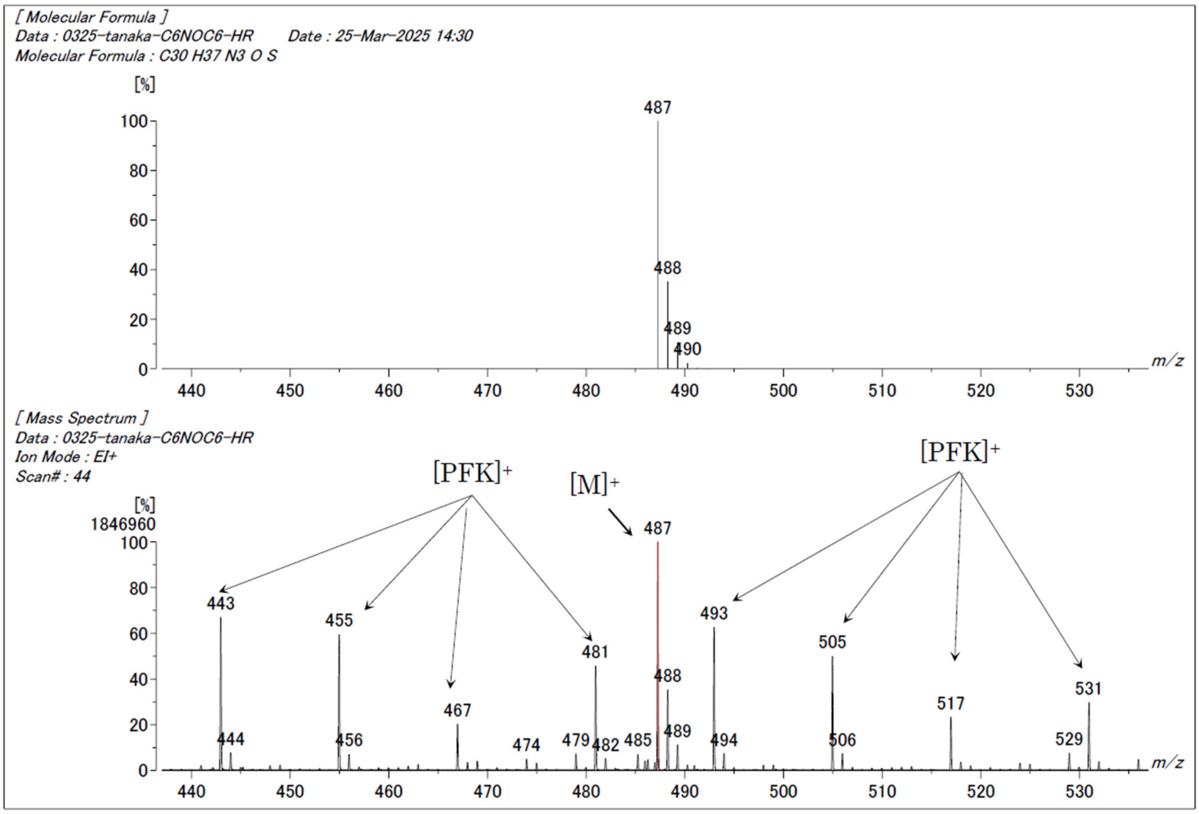

Figure S105. HR-MS spectra of **C6NOC6** (above: calculated data; below: experimental data).

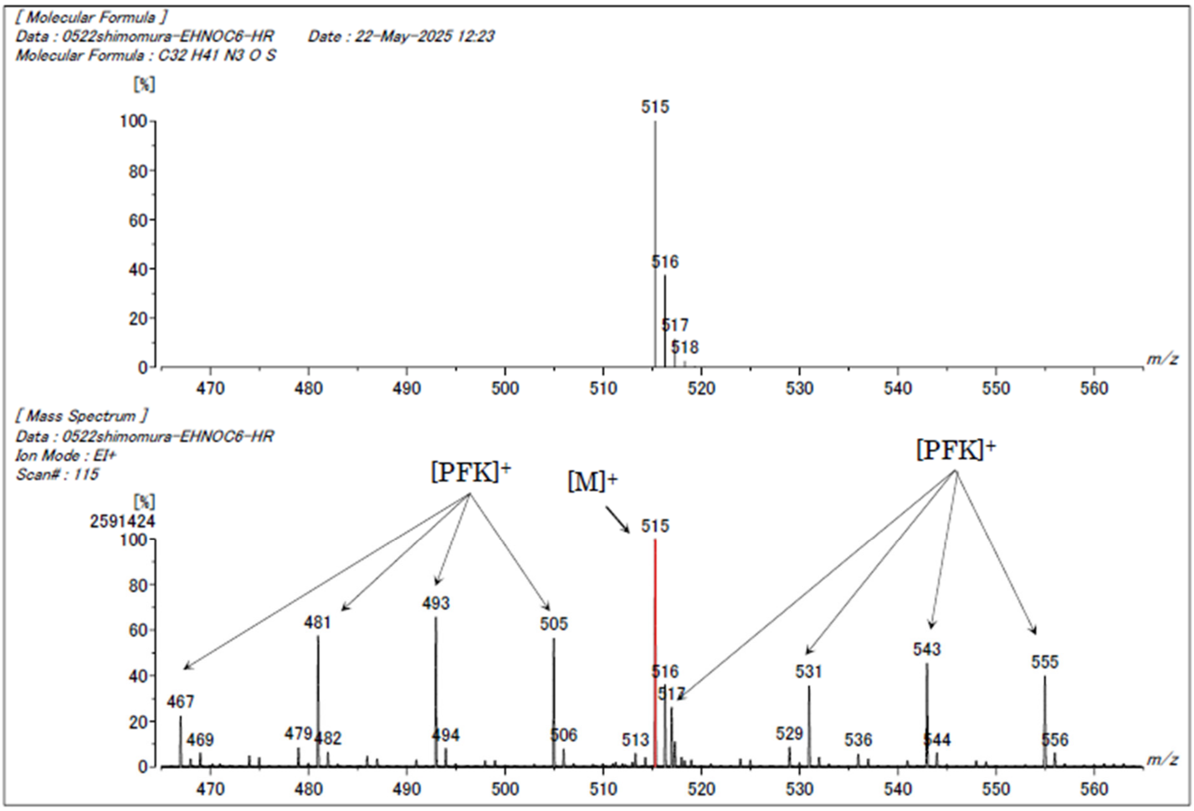

Figure S106. HR-MS spectra of **EHNOC6** (above: calculated data; below: experimental data).

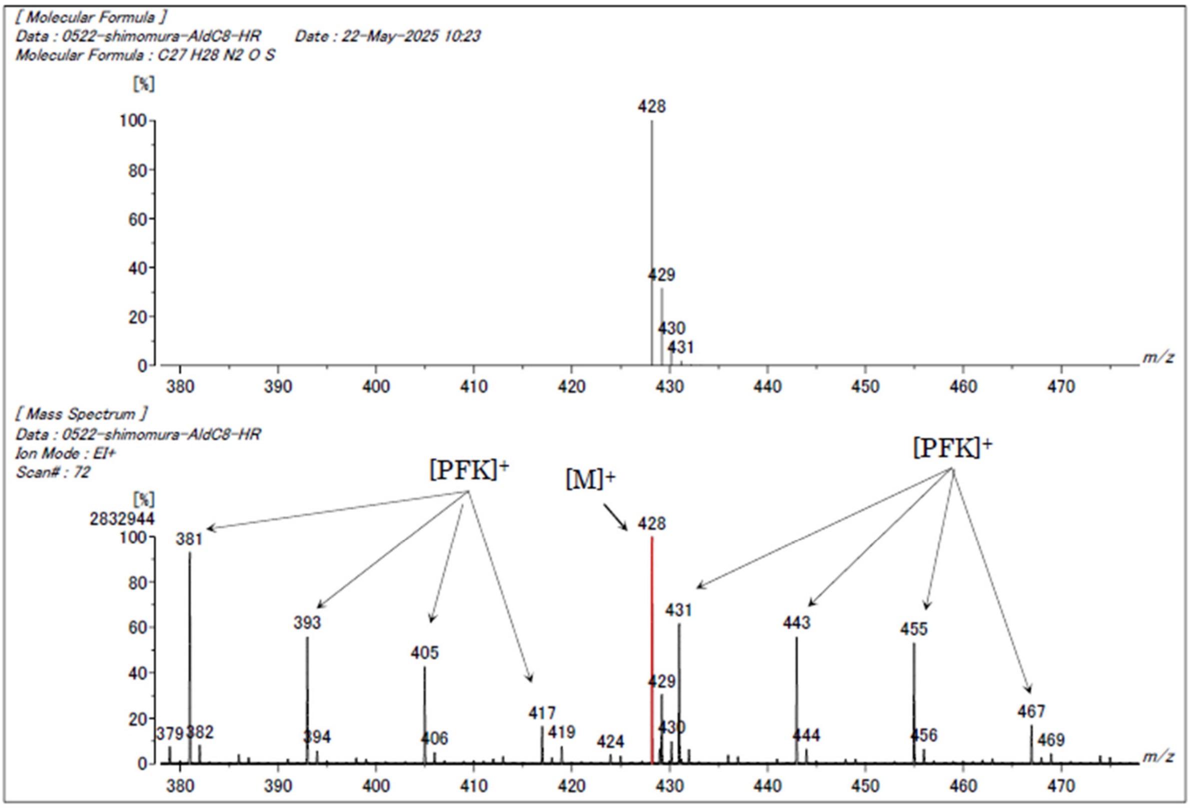

Figure S107. HR-MS spectra of **AldC8** (above: calculated data; below: experimental data).

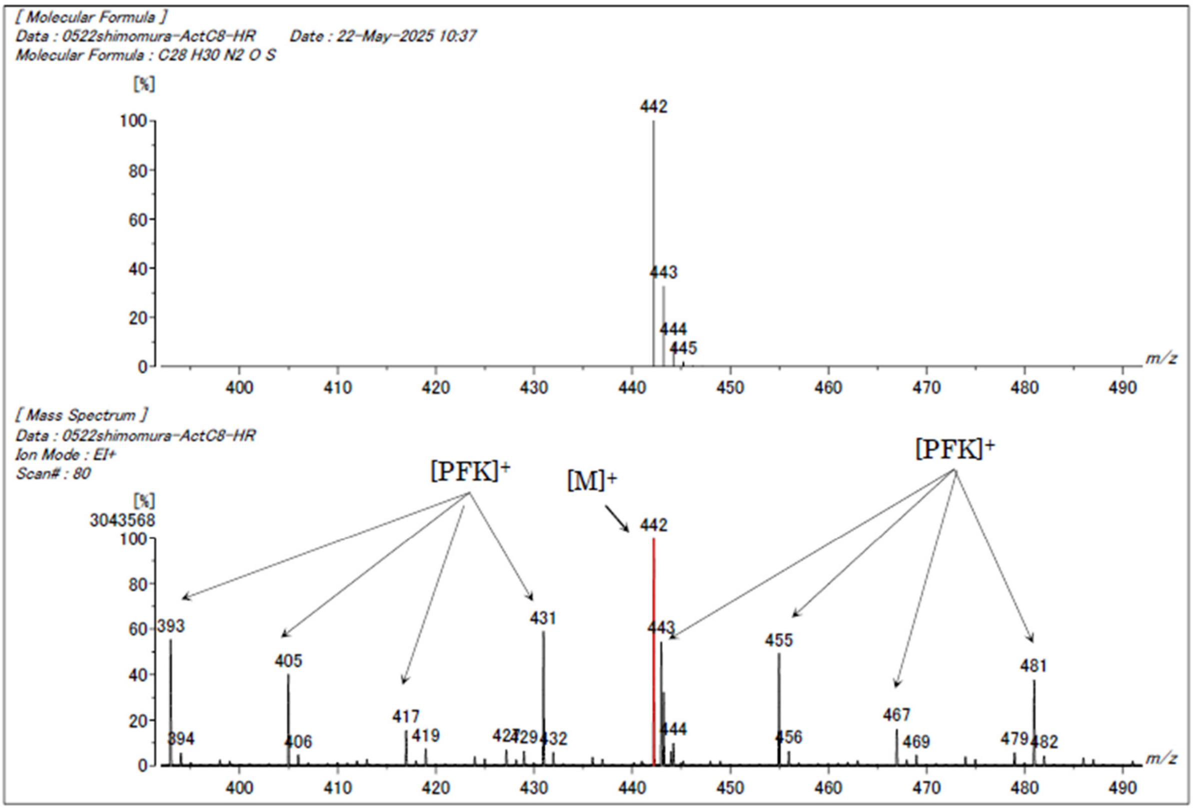

Figure S108. HR-MS spectra of **ActC8** (above: calculated data; below: experimental data).

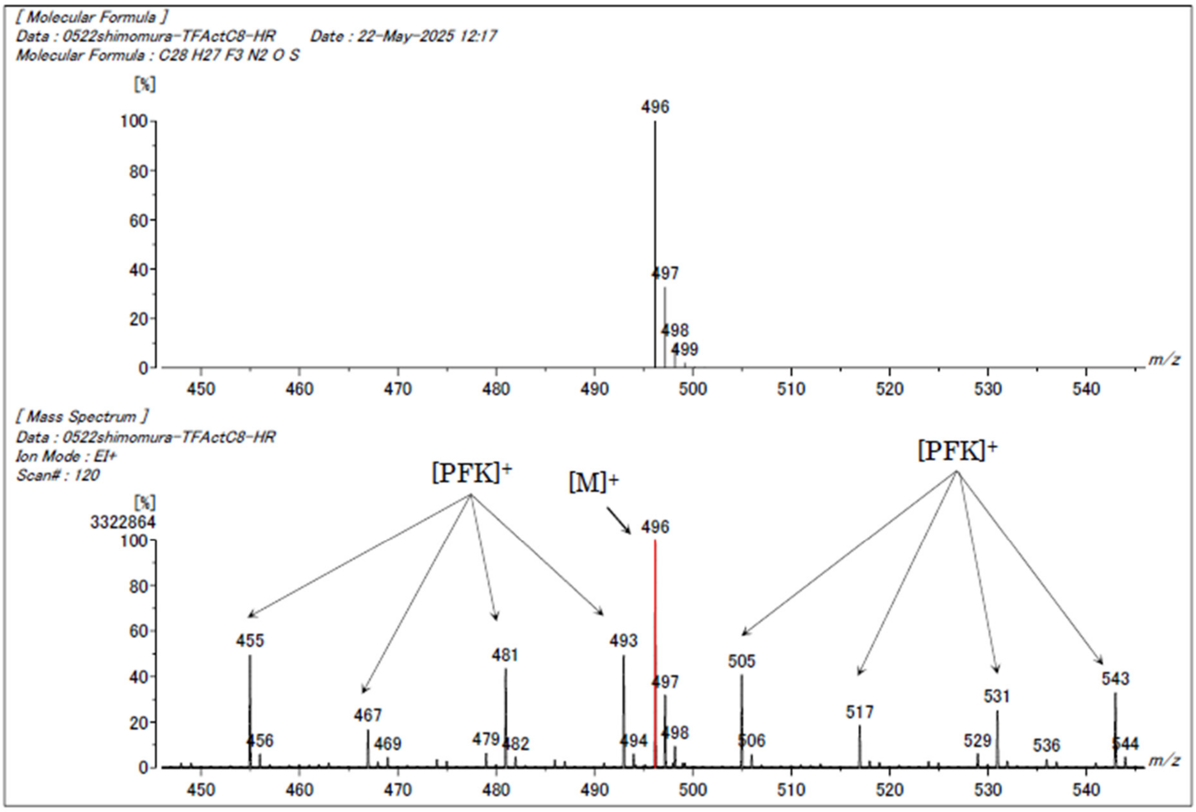

Figure S109. HR-MS spectra of **TFActC8** (above: calculated data; below: experimental data).

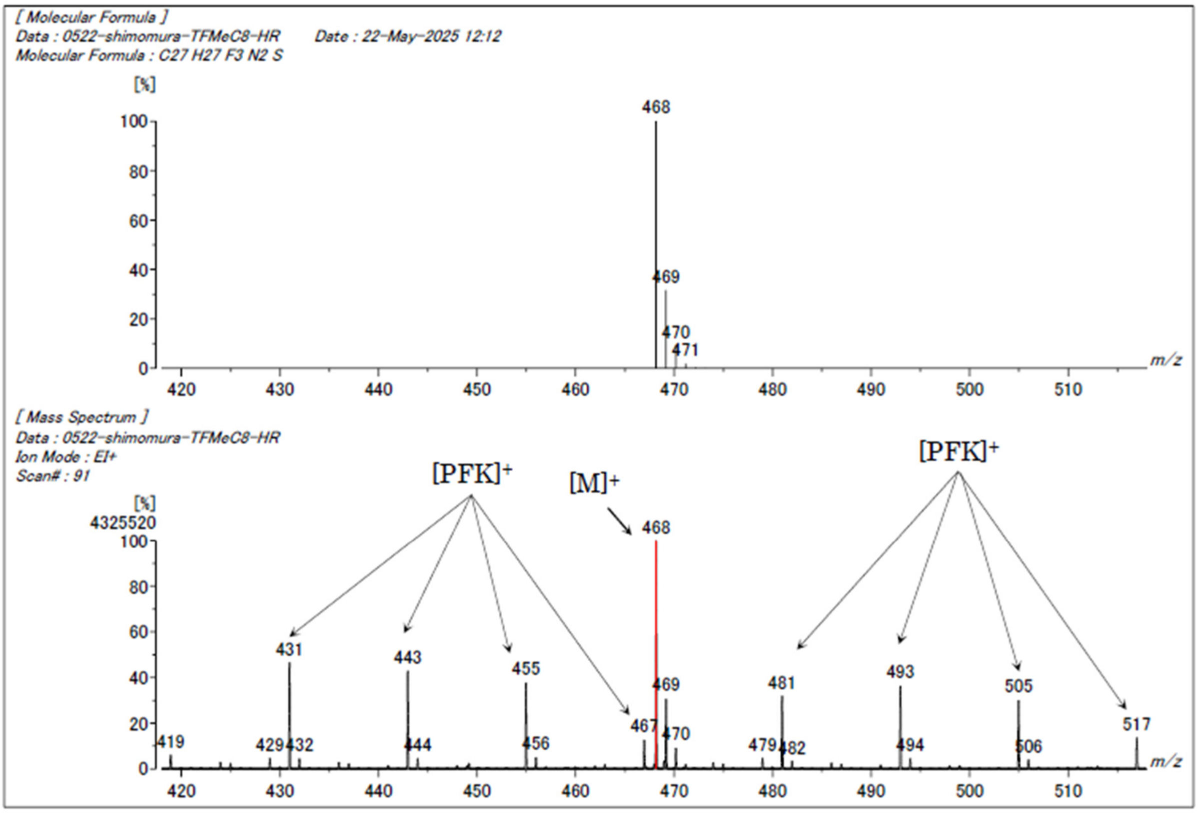

Figure S110. HR-MS spectra of **TFMeC8** (above: calculated data; below: experimental data).

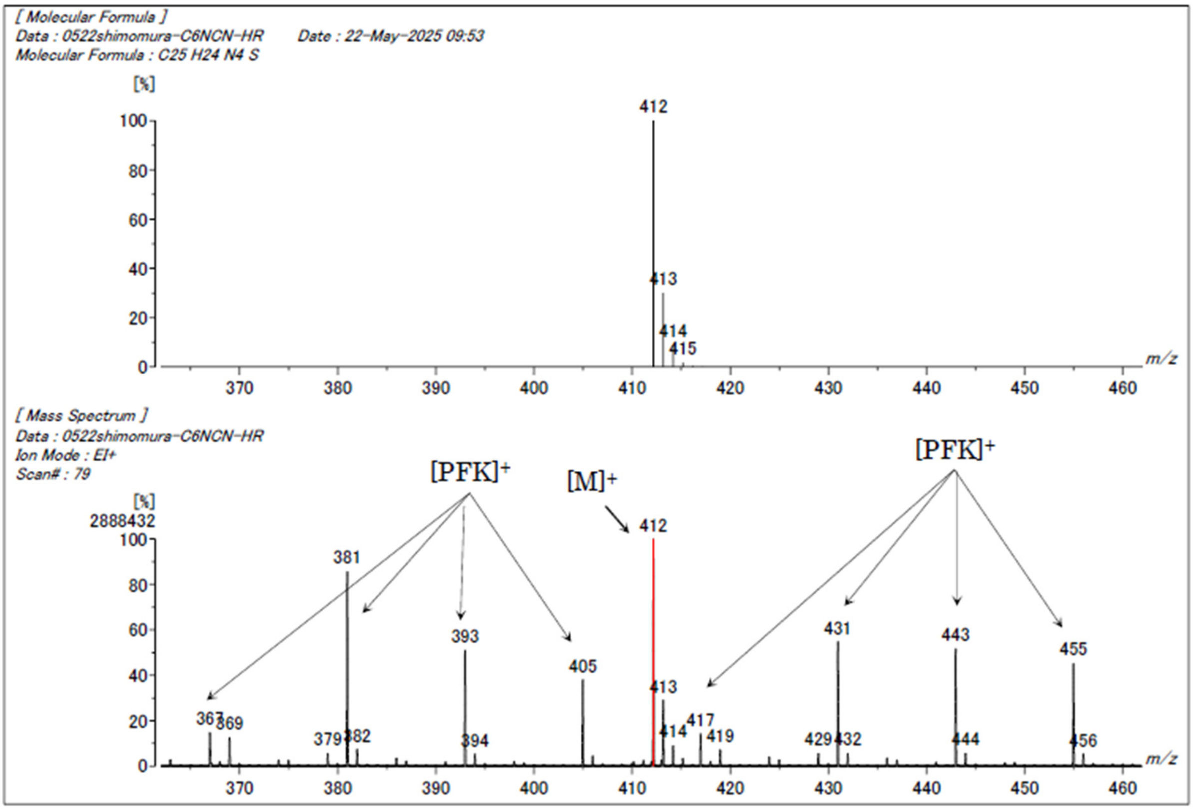

Figure S111. HR-MS spectra of **C6NCN** (above: calculated data; below: experimental data).

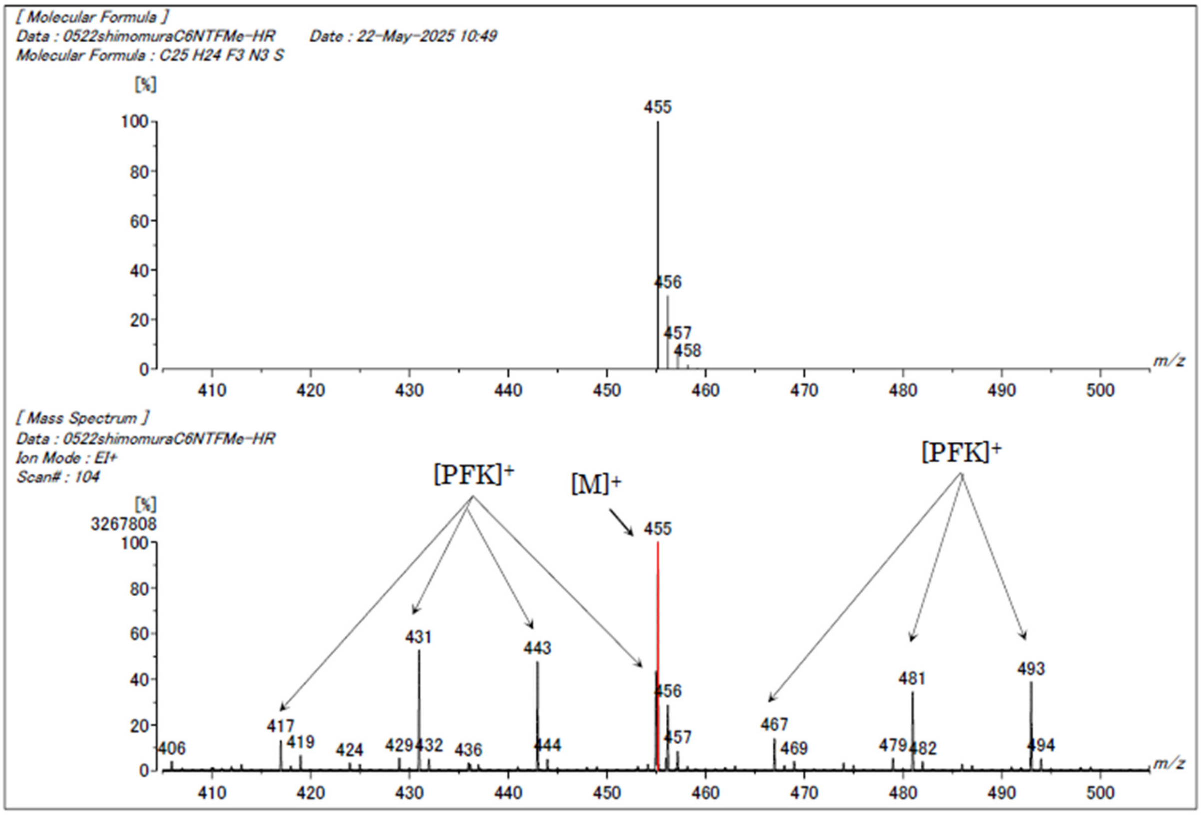

Figure S112. HR-MS spectra of **C6NTFMe** (above: calculated data; below: experimental data).

Additional experiment (WAXD)

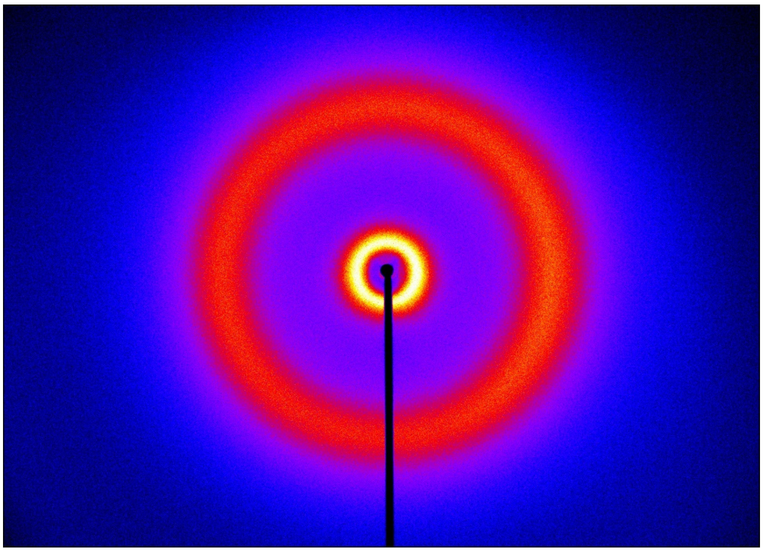

Figure S113. A WAXD image of **EHNOC6** at 38 °C on cooling.

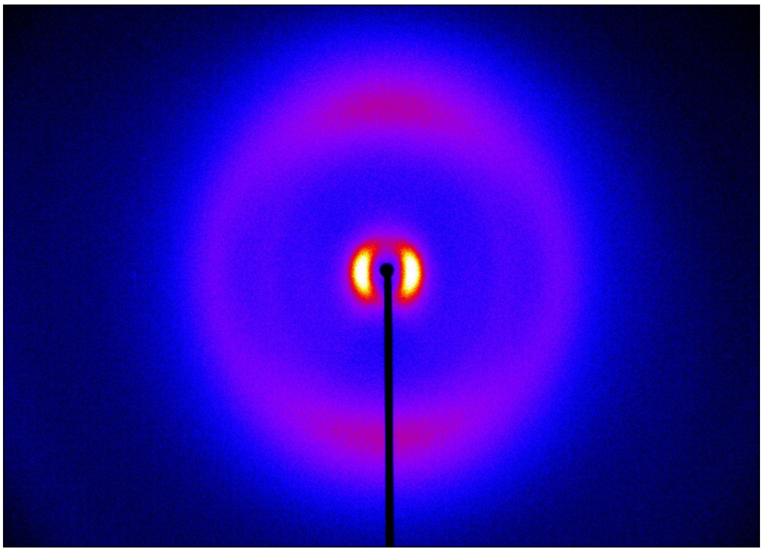

Figure S114. A WAXD image of **TFActC8** at 33 °C on cooling.
